# Opioid Antagonists from the Orvinol Series as Potential Reversal Agents for Opioid Overdose

**DOI:** 10.1021/acschemneuro.2c00464

**Published:** 2022-10-12

**Authors:** Alex Disney, Keith M. Olson, Amanda M. Shafer, Sierra C. Moore, Jessica P. Anand, John R. Traynor, Stephen M. Husbands

**Affiliations:** † Medicinal Chemistry Section, Department of Life Sciences, University of Bath, Bath BA2 7AY, U.K.; ‡ Department of Pharmacology and Edward F Domino Research Center, University of Michigan, Ann Arbor, Michigan 48109 United States; § Department of Medicinal Chemistry, University of Michigan, Ann Arbor, Michigan 48109 United States

**Keywords:** opioid overdose, opioid antagonist, mu-opioid antagonist, kappa-opioid antagonist, GPCR, diprenorphine

## Abstract

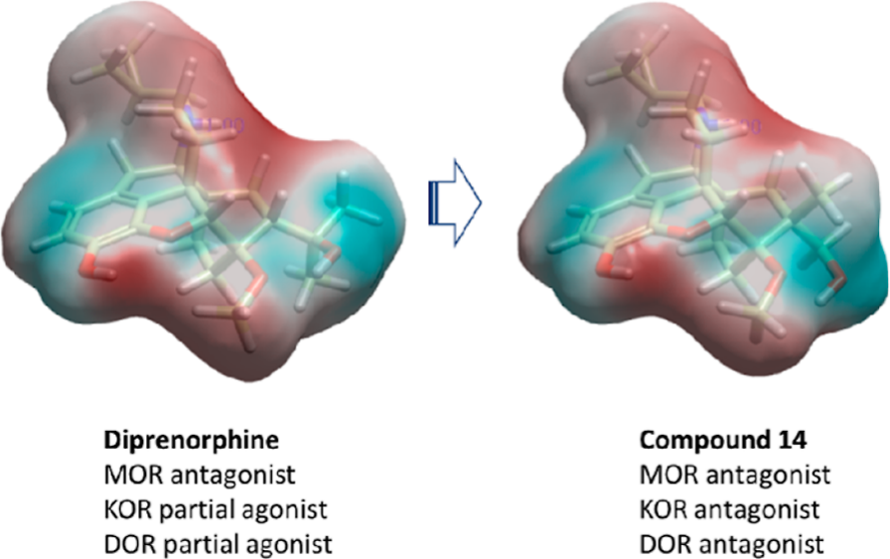

The opioid crisis continues to claim many lives, with a particular issue being the ready availability and use (whether intentional or accidental) of fentanyl and fentanyl analogues. Fentanyl is both potent and longer-acting than naloxone, the standard of care for overdose reversal, making it especially deadly. Consequently, there is interest in opioid reversal agents that are better able to counter its effects. The orvinol series of ligands are known for their high-affinity binding to opioid receptors and often extended duration of action; generally, compounds on this scaffold show agonist activity at the kappa and the mu-opioid receptor. Diprenorphine is an unusual member of this series being an antagonist at mu and only a partial agonist at kappa-opioid receptors. In this study, an orvinol antagonist, **14**, was designed and synthesized that shows no agonist activity in vitro and is at least as good as naloxone at reversing the effects of mu-opioid receptor agonists in vivo.

## Introduction

The ongoing worldwide opioid epidemic has been exacerbated by the arrival of fentanyl (and fentanyl analogues) on the illicit market. Fentanyl is a potent mu-opioid receptor (MOR) agonist and significantly contributes to the death toll resulting from the use of opioids.^1^ Fentanyl is more resistant to reversal than many standard opioids^2^ and may require multiple doses of naloxone after overdose.^3,4^ For this reason, a higher dose formulation has recently been approved by the FDA (https://www.fda.gov/news-events/press-announcements/fda-approves-higher-dosage-naloxone-nasal-spray-treat-opioid-overdose).
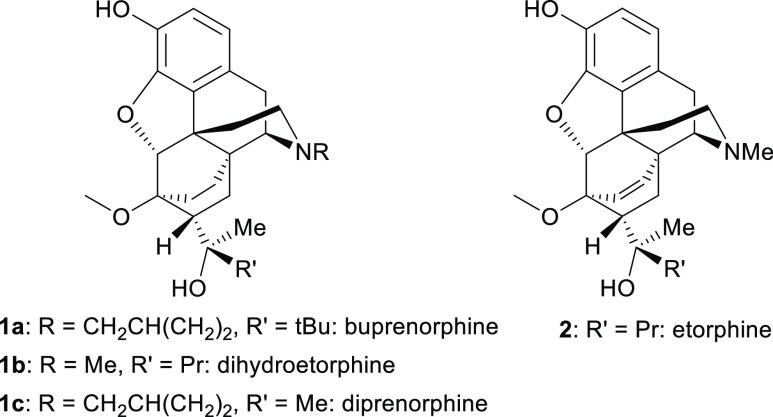


The orvinols are a series of compounds targeting opioid receptors, initially synthesized as analgesics without the undesirable side effects associated with morphine and other typical opioid analgesics.^5^ Generally, orvinols bind equally well to each of the three classical opioid receptors, MOR, kappa (KOR), and delta (DOR), with analogues showing different abilities to activate each opioid receptor type: for example, buprenorphine (**1a**) (MOR partial agonist and KOR/DOR antagonist), etorphine, and dihydroetorphine (**2**, **1b**) (full agonists at MOR, KOR, and DOR) and diprenorphine (**1c**) (MOR antagonist and DOR/KOR partial agonist).^6^ In veterinary practice, diprenorphine is a reversal agent (Revivon) for etorphine-induced immobilization, and ^11^C-diprenorphine is utilized as a positron emission tomography imaging ligand for labeling opioid receptors in both preclinical and human clinical studies. Diprenorphine could be used as an improved overdose reversal agent due to its quick onset and its effectiveness in reversing fentanyl- and morphine-induced respiratory depression.^2^ However, while often described as a MOR, KOR, and DOR opioid antagonist, diprenorphine shows partial agonist activity at both KOR and DOR;^6^ the former is substantial enough to cause psychotomimetic effects in humans,^7^ limiting its utility. Therefore, an improved rescue medication should retain diprenorphine’s MOR antagonism, rapid onset, and potency versus fentanyl but with reduced KOR agonist activity. To that end, we synthesized and evaluated C7β-methyl analogues of diprenorphine as it has previously been found that adding a C7β-methyl group to the orvinols reduces KOR efficacy without significantly affecting MOR activity.^8^

## Results and Discussion

### Chemistry

We have previously reported on the synthesis of C7β-methyl analogues of the orvinols using a Lewis acid-catalyzed reaction between *N*-cyclopropylcarbonylnorthebaine and methacrolein.^8^ There were two key issues with this approach, the first being that the scope was limited to *N*-acylated analogues of thebaine and the second being that while the overall yield of the Diels–Alder step was high, the desired 7β-methyl isomer was obtained only in a ratio of 2:3 with the undesired 7α-methyl epimer. While we and others^8,9^ have determined that thebaine (**3**) reacts poorly with methacrolein under standard conditions (organic solvent and heat), Maat et al.^10^ had reported the successful reaction of thebaine with a closely related dienophile, methyl methacrylate, simply using an extended reaction time (2 weeks) and heat (100 °C). In our hands, attempting this method led to extensive polymerization and very low yields, but it did, in the case of methacrolein, provide some products with a favorable ratio of diastereomeric products. As the use of aqueous systems to promote Diels–Alder reactions is well known,^11^ there seemed a reasonable possibility of improving these low yields. We have now found that there is a powerful aqueous solvent effect for this particular Diels–Alder reaction and that good yields (93% overall) and a far more favorable ratio of isomers can be obtained by carrying out the reaction in saturated brine at 80 °C (66% of C7β-Me **4b**/33% C7α-Me **4a** as determined by the ratio of aldehyde protons in ^1^H NMR). Thus, a much higher yield of the desired epimer is achieved, and by retaining the N–Me group, this method allows for manipulation of this N-substituent by standard methods later in the synthesis. Importantly, the reaction with methacrolein has proven to be reproducible in our hand, although not so with other dienophiles.

**Scheme 1:**
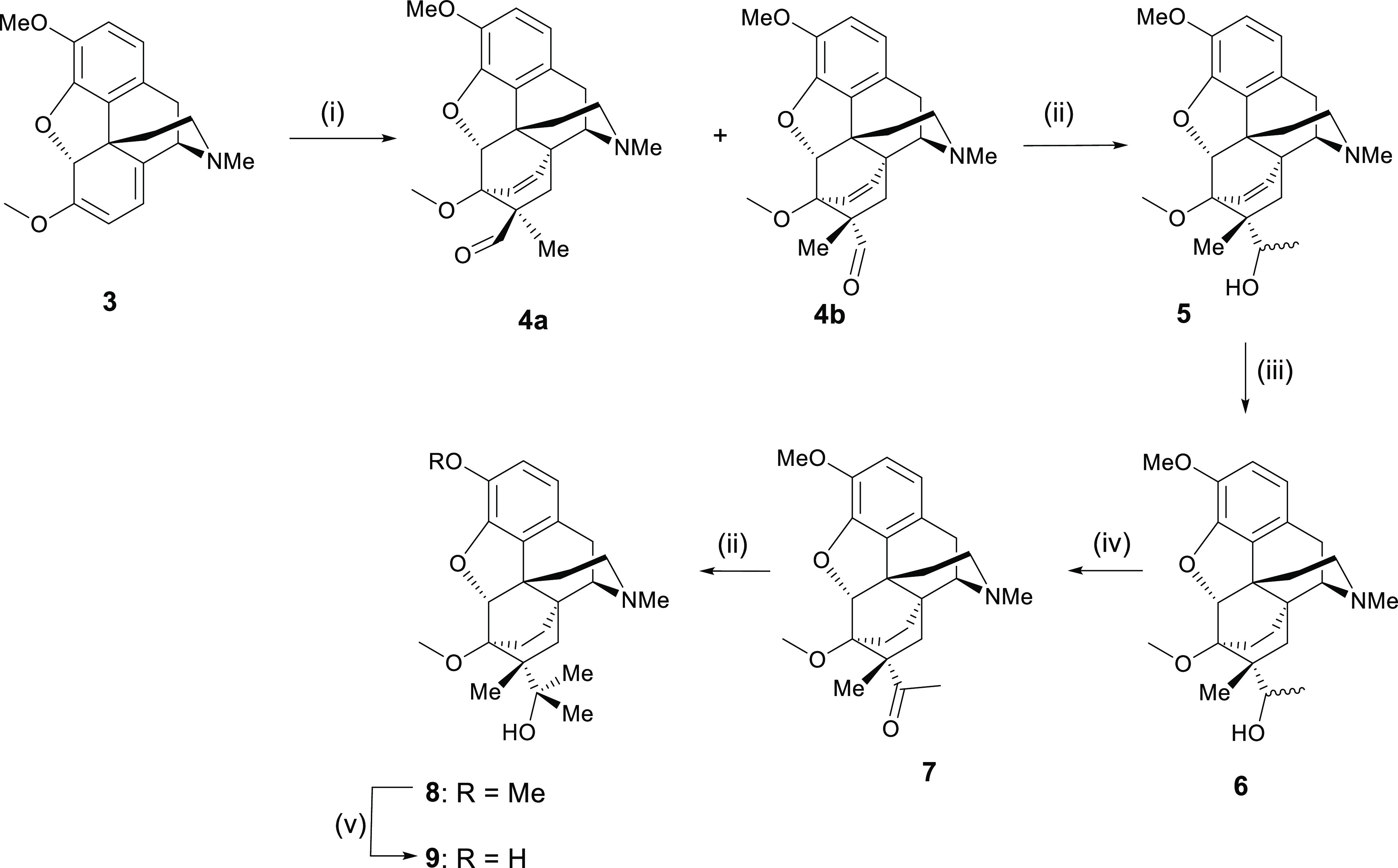
(i) Methacrolein, Brine, 80 °C; (ii) MeLi, Toluene, −78 °C—RT; (iii) H_2_, 10% Pd/C, EtOH; (iv) DMPI, CH_2_Cl_2_; (v) NaH, PrSH, HMPA, 110 °C

After isolation of the desired C7β-Me isomer **4b**, the addition of MeLi gave a mixture of diastereomeric 2° alcohols (**5**, in a 2:1 ratio by NMR) that were hydrogenated over Pd/C to reduce the bridge (**6**) and then oxidized to methyl ketone (**7**). Further treatment with MeLi and subsequent 3-O-demethylation furnished the C7β-Me tertiary alcohol (**9**) (Scheme 1).

**Scheme 2:**
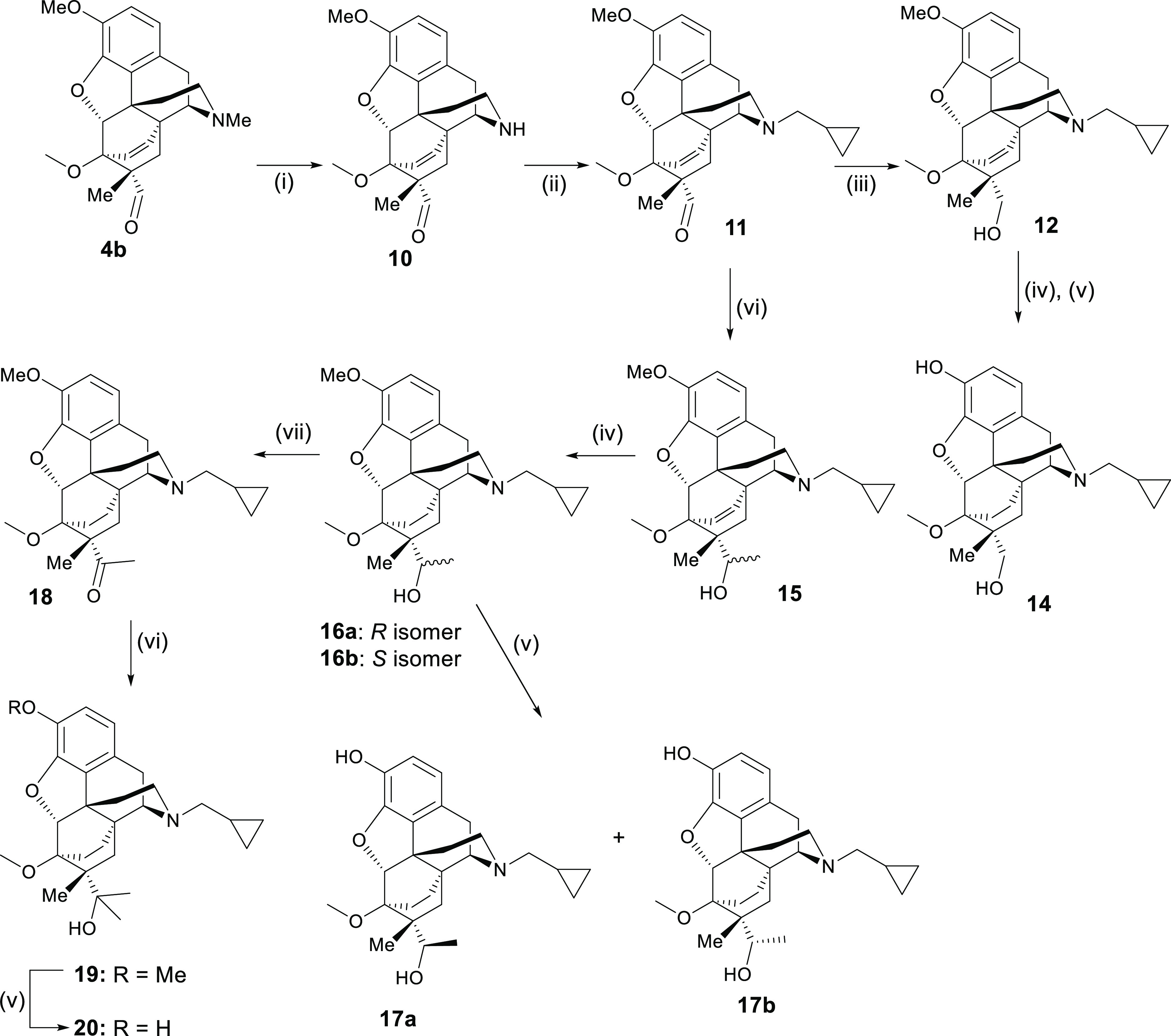
(i) DIAD, Pyridine HCl, MeCN, Reflux; (ii) KHCO_3_, Cyclopropylmethyl bromide, DMF, 50 °C; (iii) NaBH_4_, EtOH; (iv) H_2_, 10% Pd/C, EtOH; (v) NaH, PrSH, HMPA, 110 °C; (vi) MeLi, Toluene, −78 °C—RT; (vii) DMPI, K_2_CO_3_, CH_2_Cl_2_

To access the *N*-cyclopropylmethyl series (Scheme 2), *N*-demethylation of the Diels–Alder adduct (**4b**) followed by alkylation with cyclopropylmethyl bromide gave the key intermediate (**11**). Reduction afforded the 1° alcohol (**12**) with subsequent hydrogenation of the bridge and 3-O-demethylation, yielding **14**. Alternatively, treatment of **11** with MeLi gave diastereomeric 2° alcohols **15** (in a 3:1 ratio by ^1^H NMR), subsequently hydrogenated to **16a** and **16b**, which could be separated by column chromatography and then converted into their phenolic counterparts **17a** and **17b**. Otherwise, the diastereomeric mixture of **16a** and **16b** could be O-demethylated with the products **17a** and **17b** and then separated by silica gel chromatography. The stereochemistry of the secondary alcohols was assigned based on the crystal structure of the individual C20-diastereoisomer **16a** (Supporting Information). Oxidation of 2° alcohols (**16**) provided the methyl ketone (**18**) with Grignard addition and subsequent 3-O-demethylation, providing **20**, the C7β-Me analogue of diprenorphine.

### Biological Evaluation

Binding affinities of the new compounds to opioid receptors were determined by the displacement of [^3^H]-diprenorphine binding from CHO membrane homogenates expressing human (h) MOR, KOR, or DOR. As expected, diprenorphine (**1c**) and the synthesized analogues—**9**, **14**, **17a**, **17b**, and **20**—showed high-affinity binding to MOR, KOR, and DOR (Table 1) with minimal selectivity. In fact, compounds **14**, **17a**, and **17b**, where R^1^ = cyclopropylmethyl, showed subnanomolar affinity at all three opioid receptors. The N–Me analogue (**9**) maintained the lack of selectivity but with a somewhat lower affinity for all three receptors. This lack of selectivity is consistent with the previous reports on the C7β-Me series^8^ and with standard orvinols.^5,12,13^

**Table 1:**
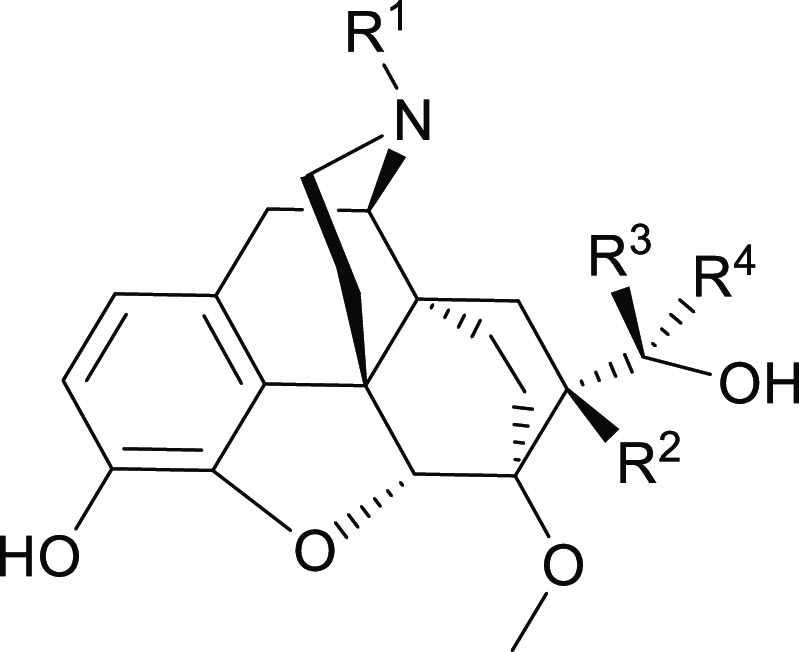
Binding Affinities (Ki/nM) to MOR, KOR, and DOR

					Ki (nM)^a^		
	R^1^	R^2^	R^3^	R^4^	MOR	KOR	DOR
**9**	Me	Me	Me	Me	9.7 ± 2.5	5.5 ± 2.0	17 ± 8
**14**	CPM	Me	H	H	0.27 ± 0.24	0.17 ± 0.10	0.47 ± 0.25
**17a**	CPM	Me	Me	H	0.14 ± 0.05	0.15 ± 0.05	0.45 ± 0.15
**17b**	CPM	Me	H	Me	0.03 ± 0.01	0.35 ± 0.19	0.33 ± 0.09
**20**	CPM	Me	Me	Me	0.08 ± 0.05	0.37 ± 0.20	0.13 ± 0.05
diprenorphine **1c**	CPM	H	Me	Me	0.31 ± 0.04	0.35 ± 0.09	1.10 ± 0.06
naloxone					2.1 ± 0.2	1.0 ± 0.1	22 ± 6

a Ki (nM) determined by the displacement of [^3^H]diprenorphine in membrane homogenates of CHO cells expressing hMOR, hDOR, or hKOR. Values are means ± SEM from three separate experiments, each performed in duplicate. CPM = cyclopropylmethyl.

The relative agonist activity of the compounds was determined using the [^35^S]GTPγS assay in the same membrane homogenates as employed for the binding assays. Table 2 shows the potency of the compounds as EC_50_ values and the maximal effect of each compound compared to standard agonists DAMGO (MOR), U69593 (KOR), and SNC80 (DOR). As expected from previous reports, diprenorphine did not stimulate [^35^S]GTPγS binding to cell membrane homogenates expressing MOR but showed partial agonist activity at KOR and DOR. Analogue **9**, which has a methyl substituent on the tertiary N atom, was a potent partial agonist at MOR (Table 2). At KOR, **9** showed no agonist activity but was a low-potency partial agonist at DOR.

**Table 2:** Agonist Stimulation of [^35^S]GTPγS Binding to MOR, DOR, and KOR^a^

	MOR		KOR		DOR	
	EC_50_, nM	Emax, %^b^	EC_50_, nM	Emax, %^b^	EC_50_, nM	Emax, %^b^
**9**	1.6 ± 0.1	38.5 ± 5.0	NS		414 ± 139	32.4 ± 4.9
**14**	NS		NS		NS	
**17a**	15.7 ± 15.3	23.5 ± 9.2	236 ± 233	32.5 ± 4.1	2.6 ± 1.7	16.3 ± 1.5
**17b**	12.2 ± 12.0	22.5 ± 7.5	46.4 ± 24.4	28.0 ± 10.4	NS	
**20**	99.3 ± 45.0	23.7 ± 10.3	NS		11.1 ± 6.7	17.0 ± 2.2
diprenorphine **1c**	NS		1.5 ± 0.2	30.9 ± 1.6	8.7 ± 4.0	41.6 ± 2.2
DAMGO	32.2 ± 7.7	102 ± 1.8	ND	ND	ND	ND
U69,593	ND	ND	6.2 ± 3.4	107 ± 4.7	ND	ND
SNC80	ND	ND	ND	ND	2.3 ± 0.4	103 ± 0.6

a Determined in membrane homogenates of CHO cells expressing hMOR, hDOR, or hKOR.

b Percent maximal stimulation with respect to the standard agonists DAMGO (MOR), U69, 593 (KOR), and SNC80 (DOR). Values are means ± SEM from three separate experiments, each performed in duplicate. NS—no stimulation observed up to 10 μM. N.D. not determined. For structures, see Table 1.

Compound **20** in which the N–Me group of **9** is replaced with *N*-cyclopropylmethyl retained partial agonist activity at MOR and DOR, with lower potency at MOR but improved potency at DOR. Like **9**, compound **20** did not activate KOR. In the previously reported C20-phenyl secondary alcohol series having a C7β-Me substituent,^8^ the most significant piece of structure–activity relationship (SAR) was the reduction in efficacy at KOR compared to C7β-H counterparts. Therefore, we did predict that **20** [the C7β-Me analogue of diprenorphine (**1c**)] would show lower KOR efficacy than the parent diprenorphine (**1c**). **20** is, in fact, a low efficacy partial agonist at MOR and DOR but a KOR antagonist, whereas diprenorphine (**1c)** is a DOR and KOR partial agonist and a MOR antagonist.
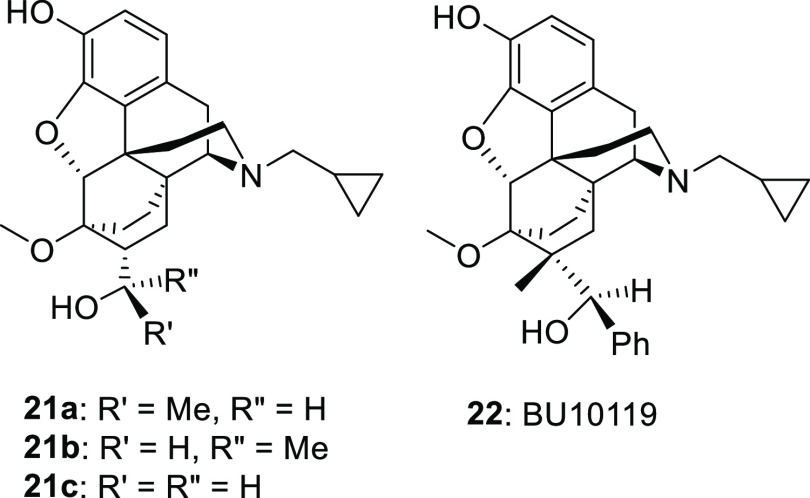


Knowing that the addition of any steric bulk to C20 would increase efficacy, particularly at MOR and KOR,^12^ we investigated the effect of moving between 1, 2, and 3° methyl alcohols at this position. **17a** and **17b**, the diastereomeric secondary alcohols, were partial agonists at MOR and KOR, with little-to-no observable agonist stimulation at DOR in the [^35^S]GTPγS assay. The corresponding secondary alcohol orvinols with C7β-H (**21a** and **21b**) and the primary alcohol **21c** have previously been described as potent antagonists with minimal efficacy at MOR, KOR, or DOR based on their lack of antinociceptive efficacy in the rat tail pressure and abdominal stretch assays.^14^

**Figure 1:**
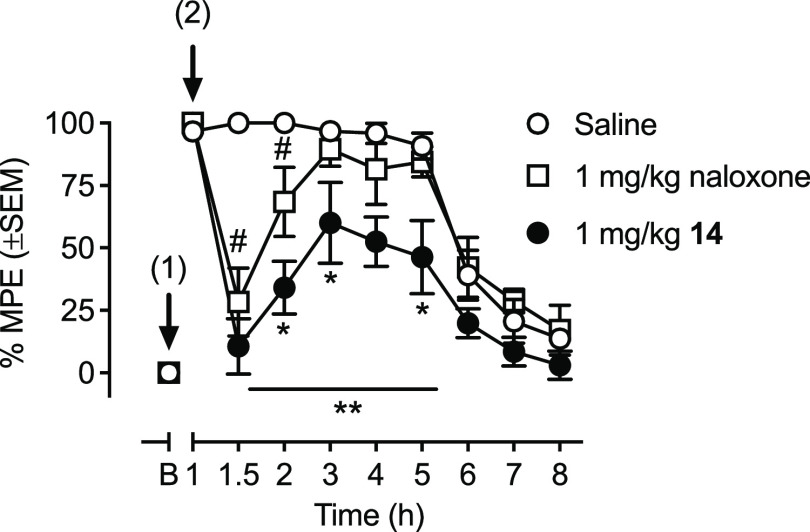
Effect of **14** or naloxone (1 mg/kg, i.p.) on antinociception produced by BU72 (0.32 mg/kg i.p.) in the tail-withdrawal assay in mice (*n* = 6 in each group). Arrow (1) shows the time of administration of BU72, and arrow (2) shows the time of administration of the antagonists or saline. B = predrug baseline response. # Naloxone significantly different from saline (*p*: 0.01); ** **14** significantly different from saline (*p* ≤0.005); * **14** significantly different from naloxone (*p* ≤ 0.05).

The only compound in the current 7β-Me series that showed no ability to stimulate [^35^S]GTPγS binding at MOR, KOR, or DOR was the 1° alcohol **14**. Yet, **14** bound with high affinity to each receptor, including an 8-fold higher affinity at MOR than naloxone, indicating nonspecific antagonist activity at the opioid receptors. The fact that **14** and naloxone are both MOR antagonists means that their affinities measured by ligand binding assay will match affinities measured by pharmacological assay, indicating that **14** is a more effective antagonist. We therefore examined the ability of **14** and naloxone to reverse MOR-mediated antinociception and respiratory depression in mice. For MOR-mediated antinociception, we chose to use the highly potent, long-lasting agonist BU72, which we have previously shown in the mouse warm-water tail-withdrawal (WWTW) assay is fully effective at a dose of 0.32 mg/kg and exhibits an antinociceptive action for at least 5 h^15^ (Figure 1), thus allowing us to readily differentiate the time course of naloxone and **14**. When given at the peak effect of BU72 (1 h), both naloxone and **14** (1 mg/kg) fully reversed the antinociceptive effects of BU72 (Figure 1). The antinociceptive effects completely returned 2 h after naloxone administration. In contrast, **14** continued to partially antagonize BU72 for as long as its antinociceptive action was evident.

**Figure 2:**
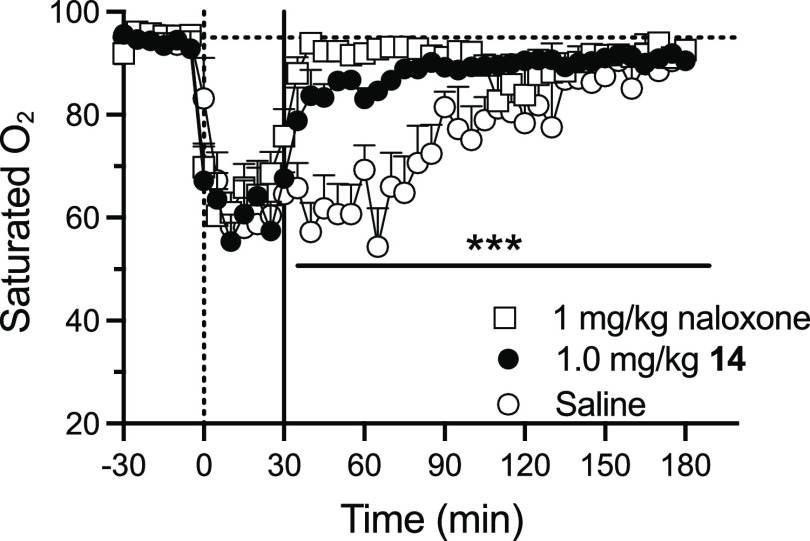
Fentanyl-induced respiratory depression and reversal with naloxone and compound **14**. Baseline measurements were taken for all groups (*n* = 7) for 30 min. At *t* = 0 min, 10 mg/kg i.p. fentanyl was administered. At *t* = 30 min, either saline, 1 mg/kg naloxone, or 1 mg/kg **14** was given i.p. *During the 2 h postantagonist administration, saline treatment was significantly different from **14** or naloxone (*p* < 0.001); **14** and naloxone were not different.

The success of **14** in reversing the antinociceptive effects of BU72 prompted us to evaluate its ability to reverse fentanyl (10 mg/kg, i.p.)-induced respiratory depression as measured by blood oxygen levels (Figure 2). Again, we compared **14** with the FDA-approved standard of care, naloxone; other compounds, in particular, diprenorphine are more potent than naloxone, but diprenorphine is not approved for opioid overdose reversal in humans and has kappa- and delta-opioid agonist activity which could confound our findings. We used fentanyl as the orthosteric agonist in this case due to its strong respiratory depressant action that contributes significantly to opioid overdose deaths. We found that **14** (1 mg/kg i.p.) reversed the fentanyl-induced decrease in oxygen saturation in a manner comparable to the same dose of naloxone (Figure 2). Both compounds had rapid onset. There was no significant difference in the effectiveness of the two compounds. However, since **14** was longer lasting than naloxone in the WWTW assay, it is possible that complete dose–response curves using lower doses of the antagonists might uncover differences. This is relevant since doses used in mice will not necessarily translate to humans where much lower doses are effective. For example, the dose of naloxone used to reverse opioid overdose in humans is 4–8 mg intranasally.^16^

In this report, we expand upon SAR around the C7β-Me orvinol series and identify **14** as a high-affinity pan opioid antagonist that reverses MOR-mediated antinociception and respiratory depression. Considering both the results reported here and those reported previously for **22** and analogues,^8^ the C7β-Me series as a whole has similar efficacy to the C7β-H series at MOR. Interestingly, the N–Me containing (**9**) had only ∼1.5 times higher efficacy at MOR than the *N*-CPM containing **17a**, **17b**, and **20**. Therefore, the change in efficacy between N–Me and *N*-CPM in this series appears to be more modest than found in the closely related 7,7-spiro series^17^ or in the standard orvinol^5,14^ or the morphinan series (e.g., compare oxymorphone with naltrexone). At KOR, the 7β-Me series overall has lower efficacy than standard orvinols (C7β-H) but similar SAR, in which efficacy is higher for 2° alcohols than for 3° alcohols; Greedy et al.^12^ showed that 2° alcohols in the standard series have higher efficacy than 3° alcohols. At DOR, the emerging evidence is that the 7β-Me ligands have lower efficacy than the standard orvinols.

## Conclusions

The new Diels–Alder conditions reported in this paper allow access to a greater range of *N*-substituents in the C7β-Me series due to easy access to the nor-intermediate **10**. The new conditions also improve the β-Me/α-Me ratio, with a resultant increase in the yield of the desired isomer. Of the compounds synthesized and evaluated, **14** stands out as a potent antagonist across all three receptors, which is unique among the orvinol series. In the mouse WWTW assay, **14** (1 mg/kg) was able to reverse the effects of the long-acting MOR agonist BU72 more effectively and for longer than the same dose of the FDA-approved reversal agent naloxone and was able to reverse fentanyl-induced respiratory depression. The improved profile of **14** over naloxone is likely due, at least in part, to its higher affinity for MOR. Whether the complex pharmacokinetics of the orvinol series that can result in long half-lives plays a role is an avenue for future investigation. Overall, our data suggests that **14** could be the starting point for the development of a novel rescue therapy.

## Methods

### Chemistry

#### Synthesis

All reactions were carried out under nitrogen unless otherwise stated. Reagents and solvents were purchased from Sigma-Aldrich or Alfa Aesar and used without further purification. ^1^H and ^13^C NMR spectra were obtained using a Bruker 400 MHz instrument (^1^H at 400 MHz, ^13^C at 100 MHz); δ is given in ppm, *J*, in Hz. Column chromatography was performed using RediSep prepacked columns with a Teledyne Isco CombiFlash instrument. Ligands were tested as their hydrochloride salts, prepared by adding 2.5 equiv of HCl (1 N solution in diethyl ether) to a solution of the compound in anhydrous diethyl ether.

#### HPLC–ESI–TOF Analysis

HPLC–ESI–TOF analysis was conducted using an electrospray time-of-flight (MicrOTOF) mass spectrometer (Bruker Daltonik GmbH, Bremen, Germany), which was coupled to an Agilent HPLC stack (Agilent, Santa Clara, CA, United States) consisting of an Agilent G1312A binary pump with a G1329A autosampler and G1316A column oven. Analyses were performed in ESI positive and negative modes. The capillary voltage was set to 4500 V, the nebulizing gas at 2.2 bar, and the drying gas at 10.2 L/min at 220 °C in each case. The TOF scan range was from 50 to 700 mass-to-charge ratio (*m*/*z*). The system was configured with a switching valve to perform flow injections or chromatography. In each case, 10 μL injections were made. The TOF was calibrated with a 10 μL sodium formate calibrant solution injection prior to the chromatographic/flow injection run. The calibrant solution consisted of three parts of 1 M NaOH to 97 parts of 50:50 water/isopropanol with 2% formic acid. Automated data processing was performed using the Compass Data Analysis software scripts (Bruker Daltonik GmbH, Bremen, Germany).

#### UHPLC Method

The UHPLC analysis was conducted using an Ultimate 3000 UHPLC (Thermo Fisher Scientific, California, USA). Liquid chromatography was performed using a Kinetex XB-C18 1.7 μM, 100 Å, 2.1 × 50 mm column (Phenomenex) with a flow rate of 0.4 mL/min at 25 °C. The Ultimate 3000 RS autosampler, fitted with a 100 μL loop, was used to make injections of 5 μL. Mobile phases A and B consisted of 0.1% trifluoroacetic acid (TFA, Sigma-Aldrich, protein sequencing grade) in H_2_O (MS grade, VWR) and 0.1% TFA in acetonitrile (HiPerSolv, HPLC grade, VWR). The longer chromatographic separation method was carried out with initial 10% mobile phase B conditions until 3 min, followed by a linear gradient to 100% B to 20 min, keeping 100% B up until 24 min, and thereafter returned to 10% B until a 28 min total run time. The shorter chromatographic separation method was carried out with initial 1% mobile phase B conditions up to 3 min, followed by a linear gradient to 100% B at 8 min, keeping 100% B up until 13 min, and then returned to 1% B until an 18 min total run time. An Ultimate 3000 variable wavelength detector was operated at 254 and 280 nm with a data collection rate of 2.5 Hz and a time constant of 0.6 s. Data processing was performed using the Data Analysis software version 4.3 (Bruker Daltonik GmbH, Bremen, Germany).

##### 4,5-Epoxy-3,6-dimethoxy-7β,17-dimethyl-6,14-ethenomorphinan-7α-carboxaldehyde (**4b**)

Thebaine (25.0 g, 8.029 × 10^–2^ M) was combined with methacrolein (50 mL, 0.6042 M, 7.5 equiv) and brine in equal volume. The mixture was stirred vigorously and heated to 80 °C for 14–21 days. The mixture was periodically checked by TLC. When complete, the brine layer was separated. The organic layer was combined with HCl (aq) [1.0] (50 mL) and washed with diethyl ether (4 × 25 mL), and the aqueous phase was retained. The pH of the aqueous phase was adjusted to 8.0–8.5 and then extracted with DCM (4 × 25 mL). The organic phase was dried with MgSO_4_ and concentrated under vacuum. The resultant waxy solid (29.34 g, 93% yield) consisted of a 1:2 ratio of C7α-methyl/C7β-methyl (**4a**:**4b**) as determined by integration of the C20 aldehyde proton. Purification by normal-phase silica flash chromatography, using hexane to EtOAc, afforded the β-methyl diastereomer **4b** 16.40 g, 52%. ^1^H NMR (CDCl_3_): δ = 9.50 (s, 1H), 6.63 (d, 1H, *J* = 8.2 Hz), 6.54 (d, 1H, *J* = 8.2 Hz), 6.08 (dd, 1H, *J* = 8.8, 1.3 Hz) 5.55, (d, 1H, *J* = 8.8 Hz), 4.93 (d, 1H, *J* = 1.5 Hz), 3.83 (s, 3H), 3.69 (s, 3H), 3.48 (d, 1H, *J* = 5.1 Hz), 3.24–3.20 (m, 1H), 2.61–2.57 (m, 1H), 2.50–2.41 (m, 3H), 2.40 (s, 3H), 2.38–2.31 (m, 1H), 1.85–1.80 (m, 2H), 1.35 (s, 3H) ppm; ^13^C NMR (CDCl_3_): δ = 204.2, 147.7, 142.1, 137.2, 134.6, 127.8, 126.6, 119.4, 114.0, 94.4, 81.5, 60.4, 56.8, 55.4, 54.6, 50.7, 47.0, 45.4, 43.5, 34.1, 31.1, 22.5, 16.1 ppm; HRMS calcd for C_23_H_28_NO_4_ [M + H]^+^, 382.2013; found, 382.2075.

##### (5α,6*R*,7*R*,14α)-1′-(4,5-Epoxy-7,8-dihydro-3,6-dimethoxy-7β,17-Dimethyl-6,14-etheno-morphinan-7-yl)-ethan-1′-ol (**5**)

Compound **4b** (3.184 g, 8.347 × 10^–3^ M) was dissolved in toluene and cooled to −78 °C, and to this was cautiously added MeLi [1.6 M] (10.4 mL, 16.693 × 10^–3^ M, 2.0 equiv); this was allowed to warm to RT and stirred for 4 h. Excess MeLi was destroyed by the careful addition of IPA followed by water. The pH was adjusted to 8.0–8.5, and the organic phase was separated. Then, it was dried with MgSO_4_ and concentrated under vacuum. This was used without further purification. (2.745 g, 83% yield). ^1^H NMR (CDCl_3_): δ = 6.63–6.61 (m, 1H), 6.52–6.49 (m, 1H), 6.07–6.04 (m, 1H), 5.45, (d, 0.7H, *J* = 8.9 Hz), 5.39, (d, 0.3H, *J* = 8.9 Hz), 5.03–5.02 (m, 1H), 4.94 (s, 0.6H), 3.82 (s, 3H), 3.75 (s, 2.2H), 3.71 (s, 0.8H), 3.67–3.64 (m, 0.4H), 3.21 (d, 1H, *J* = 18.6 Hz), 3.15 (br s, 0.3H), 3.08 (d, 0.7H, *J* = 5.3 Hz), 2.53 (br s 1H), 2.45–2.31 (m, 7H), 1.77 (d, 1.3H, *J* = 10.5 Hz), 1.43 (d, 0.6H, *J* = 13.2), 1.30 (s, 1H), 1.27 (s, 2H), 1.07 (d, 1H, *J* = 6.6 Hz), 1.02 (d, 2.0H, *J* = 6.6 Hz) ppm; ^13^C NMR (CDCl_3_): δ = 148.1, 147.7, 142.1, 135.8, 135.2, 128.1, 127.6, 126.8, 119.3, 119.2, 114.0, 95.2, 94.8, 86.5, 83.5, 74.1, 73.4, 60.6, 56.9, 56.9, 55.4, 55.2, 46.7, 46.3, 45.8, 45.6, 43.6, 43.4, 43.1, 37.1, 35.1, 31.1, 22.4, 20.8, 20.5, 16.8, 13.8 ppm; HRMS calcd for C_24_H_32_NO_4_ [M + H]^+^, 398.2326; found, 398.2396.

##### (5α,6*R*,7*R*,14α)-1′-(4,5-Epoxy-7,8-dihydro-3,6-dimethoxy-7β,17-dimethyl-6,14-ethano-morphinan-7-yl)-ethan-1′-ol (**6**)

Compound **5** (3.00 g, 7.547 × 10^–3^ M) was dissolved in EtOH and purged with nitrogen, and 10% Pd/C was added. The mixture was degassed under vacuum; then, a hydrogen balloon was applied and stirred vigorously. The reaction was checked for completion by MS. When complete, the mixture was filtered concentrated under vacuum, and used without further purification. The reaction yielded 2.77 g, 92% yield. ^1^H NMR (CDCl_3_): δ = 6.73–6.71 (d, 1H, *J* = 8.2 Hz), 6.59–6.56 (m, 1H), 5.25 (s, 0.7H), 4.93 (d, 0.7H, *J* = 2.3 Hz), 4.86 (d, 0.3H, *J* = 1.8 Hz), 4.09 (q, 0.7H, *J* = 6.1 Hz), 3.98 (quin., 0.3H, *J* = 6.3 Hz), 3.89 (s, 1H), 3.88 (s, 2H), 3.55 (s, 2H), 3.45 (s, 1H), 3.09 (d, 1H, *J* = 18.5 Hz), 2.64 (br s, 1H), 2.48–2.24 (m, 7H), 1.83–1.70 (m, 1.3H), 1.65–1.51 (m, 2.7H), 1.25–1.20 (m, 4H), 1.16–1.14 (m, 3H), 1.12–1.03 (m, 1H), 0.87–0.78 (m, 1H) ppm; ^13^C NMR (CDCl_3_): δ = 146.6, 141.9, 119.2, 119.0, 114.5, 93.3, 81.5, 77.8, 73.2, 62.0, 57.1, 57.0, 53.0, 52.4, 45.3, 44.6, 44.2, 43.6, 42.4, 40.8, 36.1, 35.9, 29.2, 29.2, 20.9, 18.1, 17.9, 17.6, 17.4, 15.3 ppm; HRMS calcd for C_24_H_34_NO_4_ [M + H]^+^, 400.2482; found, 400.2514.

##### (5α,6*R*,7*R*,14α)-1′-(4,5-Epoxy-7,8-dihydro-3,6-dimethoxy-7β,17-dimethyl-6,14-ethano-morphinan-7-yl)-ethanone (**7**)

**6** (1.0 g, 2.505 × 10^–3^ M) was dissolved in DCM, and to this was added DMPI (1.49 g, 3.507 × 10^–3^ M, 1.4 equiv). The mixture was stirred for 24 h and checked for progress by TLC. When complete, the mixture’s pH was adjusted to 8.0–8.5 and it was washed with water. The organic layer was retained and dried with MgSO_4_; then, it was filtered and concentrated under vacuum. The resultant waxy solid (1.184 g) was purified by flash chromatography (hexanes to EtOAc), affording 0.825 g, with an 83% yield. ^1^H NMR (CDCl_3_): δ = 6.68 (d, 1H, *J* = 8.0 Hz), 6.55 (d, 1H, *J* = 8.0 Hz), 4.81 (d, 1H, *J* = 2.2 Hz), 3.86 (s, 3H), 3.44 (s, 3H), 3.05 (d, 1H, *J* = 18.4 Hz), 2.74 (d, 1H, *J* = 6.3 Hz), 2.45–2.39 (m, 2H), 2.33–2.26 (m, 5H), 2.33 (s, 3H), 2.05 (dd, 1H, *J* = 13.3, 3.3 Hz), 1.63–1.51 (m, 2H), 1.47 (s, 3H), 1.37–1.29 (m, 1H), 0.95 (t, 1H, *J* = 12.3 Hz), 0.67 (t, 1H, *J* = 12.3 Hz) ppm; ^13^C NMR (CDCl_3_): δ = 212.9, 146.4, 141.8, 133.2, 128.5, 119.1, 114.4, 93.8, 76.7, 61.9, 56.9, 54.4, 52.7, 45.1, 45.0, 43.5, 36.9, 35.8, 33.4, 29.0, 27.7, 22.0, 21.5, 18.3 ppm; HRMS calcd for C_24_H_32_NO_4_ [M + H]^+^, 398.2326; found, 398.2376.

##### (5α,6*R*,7*R*,14α)-2′-(4,5-Epoxy-7,8-dihydro-3,6-dimethoxy-7β,17-dimethyl-6,14-ethano-morphinan-7-yl)-propan-2′-ol (**8**)

**7** (0.532 g, 1.338 × 10^–3^ M) was dissolved in toluene and cooled to −78 °C; to this was cautiously added MeLi [1.6 M] (1.7 mL, 2.676 × 10^–3^ M, 2.0 equiv); this was allowed to warm to RT and stirred for 24 h. The excess MeLi was destroyed by the careful addition of IPA followed by water. The pH was adjusted to 8.0–8.5, and the organic phase was separated, dried with MgSO_4_, and concentrated under vacuum. This was used without further purification, affording 0.410 g, with a 74% yield. ^1^H NMR (CDCl_3_): δ = 6.71 (d, 1H, *J* = 8.0 Hz), 6.57 (d, 1H, *J* = 8.0 Hz), 5.03 (d, 1H, *J* = 2.2 Hz), 3.88 (s, 3H), 3.75 (br s, 1H), 3.51 (s, 3H), 3.10 (d, 1H, *J* = 18.5 Hz), 2.67 (br s, 1H), 2.46–2.44 (m, 2H), 2.32–2.25 (m, 6H), 1.89 (t, 1H, *J* = 12.5 Hz), 1.77–1.69 (m, 1H), 1.62–1.50 (m, 2H), 1.47 (s, 3H), 1.39 (s, 3H), 1.28–1.20 (m, 4H), 0.81 (t, *J* = 12.3 Hz, 1H) ppm; ^13^C NMR (CDCl_3_): δ = 146.8, 141.8, 133.5, 128.7, 119.0, 114.3, 94.2, 82.0, 78.4, 62.2, 57.0, 52.6, 46.4, 45.3, 45.1, 43.6, 39.1, 36.6, 33.3, 30.3, 29.2, 27.9, 22.1, 21.2, 18.8 ppm; HRMS calcd for C_25_H_36_NO_4_ [M + H]^+^, 414.2639; found, 414.2683.

##### (5α,6*R*,7*R*,14α)-2′-(4,5-Epoxy-7,8-dihydro-3-hydroxy-6-methoxy-7β,17-dimethyl-6,14-ethano-morphinan-7-yl)-propan-2′-ol (**9**)

Compound **8** (0.130 g, 3.143 × 10^–4^ M) was dissolved in HMPA (2 mL); to this was added NaH (95%) (0.028 g, 1.167 × 10^–3^ M, 3.5 equiv) followed by propanethiol (0.1 mL, 0.84 g, 11.029 × 10^–3^ M, 3.5 equiv). The mixture was heated to 110 °C for 2 h, and the reaction progress was checked by TLC. When complete, the reaction was cooled to RT and the pH was adjusted to 8.0–8.5. The mixture was extracted with diethyl ether (4 × 25 mL), which was in turn washed with deionized water (4 × 25 mL). The organic phase was dried with MgSO_4_, filtered, and concentrated under vacuum. The resultant solids were purified by flash chromatography (hexanes to EtOAc). 0.049 g 39% yield. **9·HCl** was prepared as described above. ^1^H NMR (DMSO-*d*_6_): δ = 9.21 (s, 1H), 9.11 (br s, 1H), 6.68 (d, 1H, *J* = 8.1 Hz), 6.53 (d, 1H, *J* = 8.1 Hz), 4.88 (s, 1H), 4.38 (s, 1H), 3.56 (d, 1H, *J* = 6.8 Hz), 3.33 (s, 3H), 3.11–3.08 (m, 1H), 2.92–2.88 (m, 1H), 2.83 (d, 3H, *J* = 4.4 Hz), 2.73 (dd, 1H, ^2^*J* = 19.5, ^3^*J* = 7.4 Hz), 2.40–2.33 (m, 1H), 2.21–2.15 (m, 1H), 2.08 (s, 1H), 2.05–2.02 (m, 1H), 1.74 (d, 1H, *J* = 13.8 Hz), 1.58–1.51 (m, 1H), 1.35–1.30 (m, 1H), 1.28 (s, 3H), 1.12 (d, 6H, *J* = 10.2 Hz), 0.50 (t, 1H, *J* = 12.1 Hz) ppm; ^13^C NMR (DMSO-*d*_6_): δ = 145.9, 139.2, 131.3, 123.2, 119.3, 117.5, 92.7, 83.6, 78.8, 75.9, 62.8, 51.2, 45.9, 45.9, 42.6, 41.5, 35.8, 29.9, 28.4, 27.8, 24.2, 21.3, 17.7 ppm; HRMS calcd for C_24_H_34_NO_4_ [M + H]^+^, 400.2482; found, 400.2501; purity: 254 nm 95.80% by HPLC, *t*_R_ = 8.5 min, 280 nm 96.76% by HPLC, *t*_R_ = 8.5 min.

##### 4,5-Epoxy-3,6-dimethoxy-7β-methyl-6,14-ethenomorphinan-7α-carboxaldehyde (**10**)

**4b** (2.0 g, 5.247 × 10^–3^ M) was dissolved in MeCN (25 mL); to this was added DIAD (1.14 mL, 5.790 × 10^–3^ M, 1.1 equiv). The mixture was refluxed for 5 h; then, pyridine HCl (0.910 g, 7.870 × 10^–3^ M, 1.5 equiv) was added and the mixture was allowed to cool to RT. When cool, the mixture was concentrated under vacuum and redissolved in EtOH (25 mL); this was then allowed to stand at RT for 12 h. The resultant crystals were isolated by filtration and washed with cold fresh EtOH, then dried under vacuum to constant mass, and used without further purification, 1.4 g, 73% yield. ^1^H NMR (DMSO-*d*_6_): δ = 10.07 (br s, 1H), 9.39 (s, 1H), 8.84 (br s, 1H), 6.73 (d, 1H, *J* = 8.3 Hz), 6.62 (d, 1H, *J* = 8.3 Hz), 6.20 (d, 1H, *J* = 9.0 Hz), 5.63 (d, 1H, 8.9 Hz), 5.08 (s, 1H), 4.14 (d, 1H, 6.5 Hz), 3.74 (s, 3H), 3.59 (s, 3H), 3.27 (d, 19.5 Hz), 3.17–3.11 (m, 2H), 2.91 (br t, 1H, *J* = 11.8 Hz), 2.33 (dt, 1H, *J* = 13.9, 5.2 Hz), 1.95 (dd, 1H, *J* = 14.4, 3.1 Hz), 1.81 (d, *J* = 13.6 Hz), 1.25 (s, 3H) ppm; ^13^C NMR (101 MHz, DMSO-*d*_6_): δ = 203.1, 147.4, 142.0, 135.5, 132.6, 127.0, 125.3, 120.1, 114.9, 92.6, 80.9, 56.4, 54.9, 54.3, 51.6, 46.0, 40.8, 35.5, 32.9, 28.8, 27.0, 15.8 ppm; HRMS calcd for C_22_H_26_NO_4_ [M + H]^+^, 368.1856; found, 368.1910.

##### 17-Cyclopropylmethyl-4,5-epoxy-3,6-dimethoxy-7β-methyl-6,14-ethenomorphinan-7α-carboxaldehyde (**11**)

**10**·HCl (1.0 g, 2.721 × 10^–3^ M) was dissolved in *N*,*N*-DMF; to this were added KHCO_3_ (1.239 g, 0.123 M, 5.0 equiv) and CPMBr (0.50 g, 0.36 mL, 3.714 M, 1.5 equiv). The mixture was heated to 50 °C for 8 h, and completion was checked for by TLC. Upon completion, the mixture was concentrated to dryness under vacuum and then purified by flash chromatography (hexanes to EtOAc), affording 1.1 g, with a 96% yield. ^1^H NMR (CDCl_3_): δ = 9.47 (s, 1H), 6.63 (d, 1H, *J* = 8.2 Hz), 6.52 (d, 1H, *J* = 8.2 Hz), 6.09 (dd, 1H, ^2^*J* = 8.8, ^3^*J* = 1.3 Hz), 5.57 (d, 1H, *J* = 8.9 Hz), 4.94 (d, 1H, *J* = 1.4 Hz), 3.84 (s, 3H), 3.70 (s, 3H), 3.57 (d, 1H, *J* = 6.5 Hz), 3.10 (d, 1H, *J* = 18.4 Hz), 2.73–2.69 (m, 1H), 2.52 (d, 1H, *J* = 13.6 Hz), 2.47–2.42 (m, 2H), 2.40–2.26 (m, 3H), 1.84–1.79 (m 2H), 1.37 (s, 3H), 0.84–0.81 (m, 1H), 0.52 (t, 2H, *J* = 8.9 Hz), 0.13 (d, 2H, *J* = 4.9 Hz) ppm; ^13^C NMR (CDCl_3_): δ = 145.8, 139.2, 131.0, 123.0, 119.3, 117.6, 92.2, 76.8, 69.5, 58.6, 57.6, 51.9, 44.7, 43.8, 43.4, 36.8, 35.3, 29.8, 28.5, 24.0, 20.8, 17.5, 17.1, 5.6, 4.8, 2.8 ppm; HRMS calcd for C_26_H_31_NO_4_ [M + H]^+^, 422.2326; found, 422.2357.

##### (5α,6*R*,7*R*,14α)-(17-Cyclopropylmethyl-4,5-epoxy-7,8-dihydro-3,6-dimethoxy-7β-methyl-6,14-etheno-morphinan-7-yl)-methanol (**12**)

**11** (0.750 g, 1.78 × 10^–3^ M) was dissolved in ethanol and cooled to 0 °C; to this was cautiously added NaBH_4_ (1.25 equiv, 84.2 mg); this was allowed to warm to RT and stirred for 4 h. The excess NaBH_4_ was destroyed by the cautious addition of HCl (aq) [0.1 M]. The pH was adjusted to 8.0–8.5, and the organic phase was separated, dried with MgSO_4_, and concentrated under vacuum, affording 0.712 g, with a 94% yield. This was used without further purification. ^1^H NMR (CDCl_3_): δ = 6.63 (d, 1H, *J* = 8.1 Hz), 6.50 (d, 1H, *J* = 8.1 Hz), 6.04 (d, 1H, *J* = 8.9 Hz), 5.46 (d, 1H, *J* = 8.9 Hz), 4.98 (s, 1H), 3.84 (s, 3H), 3.74 (s, 3H), 3.66–3.59 (m, 2H) 3.44 (d, 1H, *J* = 6.5 Hz) 3.09 (d, 1H, *J* = 18.5 Hz), 2.89 (t, 1H, *J* = 10.7 Hz), 2.73–2.67 (m, 1H), 2.56 (d, 1H, *J* = 13.2, Hz), 2.44–2.33 (m, 4H), 2.32–2.27 (m, 1H), 1.78–1.76 (m, 1H), 1.42 (s, 3H), 0.86–0.79 (m, 1H), 0.67 (d, 1H, *J* = 13.3 Hz), 0.56–0.48 (m, 2H), 0.16–0.11 (m, 2H) ppm; ^13^C NMR (CDCl_3_): δ = 147.7, 142.0, 136.0, 135.5, 128.3, 126.9, 119.3, 114.0, 95.1, 85.9, 72.5, 62.8, 59.9, 57.3, 57.0, 55.4, 47.3, 44.1, 43.2, 42.6, 36.8, 31.4, 29.8, 23.1, 19.4, 9.4, 4.3, 3.3 ppm; HRMS calcd for C_25_H_34_NO_4_ [M + H]^+^, 424.2482; found, 424.2454.

##### (5α,6*R*,7*R*,14α)-(17-Cyclopropylmethyl-4,5-epoxy-7,8-dihydro-3,6-dimethoxy-7β-methyl-6,14-ethano-morphinan-7-yl)-methanol (**13**)

**12** (0.635 g, 1.499 × 10^–3^ M) was dissolved in EtOH and purged with nitrogen, and Pd (10%) on C was added. The mixture was degassed under vacuum; then, a hydrogen balloon was applied and it was stirred vigorously. The reaction was checked for completion by MS. When complete, the charcoal and palladium were filtered off, and the solution was concentrated to dryness, affording 0.528 g, with an 82% yield. ^1^H NMR (CDCl_3_): δ = 6.71 (d, 1H, *J* = 8.1 Hz), 6.55 (d, 1H, *J* = 8.1 Hz), 4.87 (d, 1H, *J* = 1.9 Hz), 3.99–3.96 (m, 2H), 3.91–3.90 (m, 1H), 3.89 (s, 3H) 3.53 (s, 3H), 3.16 (t, 1H, 10.1 Hz), 2.99–2.94 (m, 2H), 2.64–2.60 (m, 1H), 2.48 (dd, 1H,^2^ = 13.6, 3.8 Hz), 2.37–2.19 (m, 5H), 1.81–1.73 (m, 1H), 1.60–1.57 (m, 2H), 1.53–1.47 (m, 2H), 1.38 (s, 3H), 1.11–1.03 (m, 1H), 0.87 (d, 2H, *J* = 13.7 Hz), 0.80–0.73 (m, 1H), 0.48 (t, 2H, *J* = 7.2 Hz), 0.11–0.07 (m, 2H) ppm; ^13^C NMR (CDCl_3_): δ = 146.5, 141.8, 133.3, 128.6, 119.1, 114.5, 93.7, 80.9, 72.4, 59.9, 58.7, 57.0, 53.0, 45.4, 43.8, 40.5, 40.0, 35.8, 33.7, 29.1, 22.7, 21.3, 18.1, 9.4, 4.2, 3.3 ppm; HRMS calcd for C_26_H_36_NO_4_ [M + H]^+^, 426.2639; found, 426.2665.

##### (5α,6*R*,7*R*,14α)-(17-Cyclopropylmethyl-4,5-epoxy-7,8-dihydro-3-hydroxy-6-methoxy-7β-methyl-6,14-ethano-morphinan-7-yl)-methanol (**14**)

**13** (0.518 g, 1.217 × 10^–3^ M) was dissolved in HMPA; to this was added NaH (0.102 g, 4.260 × 10^–3^ M, 3.5 equiv) followed by propanethiol (0.386 mL, 0.324 g, 4.260 × 10^–3^ M, 3.5 equiv). The mixture was heated to 110 °C for 1–2 h, and the reaction progress was checked by TLC. When complete, the reaction was cooled to RT, and the pH was adjusted to 8.0–8.5. The mixture was extracted with diethyl ether (4 × 25 mL), which was in turn washed with deionized water (4 × 25 mL). The organic phase was dried with MgSO_4_, filtered, and concentrated under vacuum. The resultant solids were purified by flash chromatography (hexanes to EtOAc), affording 0.298 g, with a 59% yield; ^1^H NMR (CDCl_3_): δ = 6.70 (d, 1H, *J* = 7.9 Hz), 6.51 (d, 1H, *J* = 7.9 Hz), 5.29 (br s, 1H), 4.89 (s, 1H), 4.05 (d, 1H, *J* = 8.8 Hz), 3.97 (d, 1H, *J* = 11.1 Hz), 3.52 (s, 3H), 3.17 (t, 1H, *J* = 10.2 Hz), 2.89–2.92 (m, 2H), 2.63 (d, 1H, *J* = 7.1 Hz), 2.48 (dd, 1H, *J* = 13.6, 3.3 Hz), 2.37–2.19 (m, 5H), 1.79–1.68 (m, 2H), 1.58 (d, 1H, *J* = 10.1), 1.50 (t, 1H, 12.5), 1.38 (s, 3H), 1.11–1.03 (m, 1H), 0.89–0.83 (m, 2H), 0.78–0.75 (m, 1H), 0.48 (t, 2H, *J* = 7.0 Hz), 0.09 (d, 2H, *J* = 4.1 Hz) ppm; ^13^C NMR (CDCl_3_): δ = 145.0, 137.4, 132.9, 128.0, 119.5, 116.4, 94.2, 80.9, 72.3, 59.9, 58.7, 53.0, 45.8, 43.8, 40.5, 40.0, 35.8, 33.6, 29.0, 22.8, 21.3, 18.1, 9.4, 4.2, 3.3 ppm.

**14**·HCl: ^1^H NMR (DMSO-*d*_6_): δ = 9.28 (s, 1H), 9.01 (br s, 1H), 6.70 (d, 1H, *J* = 7.9 Hz), 6.54 (d, 1H, *J* = 7.9 Hz), 4.85 (s, 1H), 3.81 (d, 1H, *J* = 6.7 Hz), 3.46–3.37 (m, 2H), 3.36 (s, 3H), 3.31–3.24 (m, 2H), 3.12 (d, 1H, *J* = 11.6 Hz), 2.90 (sept. 1H, *J* = 4.3 Hz), 2.81 (dd, 1H, ^2^*J* = 19.9, ^3^*J* = 6.9 Hz), 2.45–2.42 (m, 2H), 1.74 (t, 2H, *J* = 14.4 Hz), 1.51–1.23 (m, 3H), 1.09 (s, 3H), 1.07 (br s, 1H), 0.75–0.54 (m, 2H), 0.61–0.54 (m, 2H), 0.37 (sex. 1H, *J* = 4.7 Hz); ^13^C NMR (DMSO-*d*_6_): δ = 145.7, 139.2, 130.7, 123.0, 119.3, 117.7, 91.7, 76.6, 66.4, 58.4, 57.5, 52.5, 44.7, 43.8, 41.2, 36.6, 35.4, 29.9, 28.5, 23.9, 20.3, 16.8, 5.6, 4.9, 2.8 ppm; HRMS calcd for C_25_H_34_NO_4_ [M + H]^+^, 412.2482; found, 412.2556; purity: 254 nm 100.00% by HPLC, *t*_R_ = 6.9 min, 280 nm 98.73% by HPLC, *t*_R_ = 6.9 min.

##### (5α,6*R*,7*R*,14α)-1′-(17-Cyclopropylmethyl-4,5-epoxy-7,8-dihydro-3,6-dimethoxy-7β-methyl-6,14-etheno-morphinan-7-yl)-ethan-1′-ol (**15**)

**11** (1.0 g, 2.372 × 10^–3^ M) was dissolved in toluene and cooled to −78 °C; to this was cautiously added MeLi [1.6 M] (2.96 mL, 4.744 × 10^–3^ M, 2.0 equiv); this was allowed to warm to RT and stirred for 4 h. The excess MeLi was destroyed by the careful addition of IPA followed by water. The pH was adjusted to 8.0–8.5, and the organic phase was separated, dried with MgSO_4_, and concentrated under vacuum. This was used without further purification, 0.927 g, 89% yield. ^1^H NMR (CDCl_3_): δ = 6.64–6.60 (m, 1H), 6.51–6.47 (m, 1H), 6.07–6.04 (m, 1H), 5.46, (d, 0.75H, *J* = 8.9 Hz), 5.41, (d, 0.25H, *J* = 8.9 Hz), 5.04 (d, 0.70H, *J* = 0.7 Hz), 5.02 (br s, 0.26H), 3.82 (s, 3H), 3.76 (s, 2.32H), 3.71 (s, 0.77H), 3.52 (d, 0.3H, *J* = 6.4 Hz), 3.44 (d, 0.8H, *J* = 6.5 Hz), 3.08 (d, 1H, *J* = 18.4 Hz), 2.72–2.68 (m, 1H), 2.47–2.33 (m, 5H), 2.31–2.25 (m, 1H), 1.77 (d, 1H, *J* = 10.5 Hz), 1.62–1.59 (m, 1H), 1.09 (d, 1H, *J* = 6.4 Hz), 1.03 (d, 2H, *J* = 6.0 Hz), 0.98 (d, 1H, *J* = 13.4 Hz), 0.87–0.79 (m, 1H), 0.56–0.48 (m, 2H), 0.16–0.10 (m, 2H) ppm; ^13^C NMR (CDCl_3_): δ = 147.7, 142.0, 136.3, 135.9, 135.6, 128.4, 128.3, 127.2, 126.6, 119.3, 119.1, 113.9, 95.0, 86.7, 73.4, 59.9, 57.3, 56.9, 56.9, 55.4, 55.2, 47.1, 46.7, 45.8, 44.2, 44.1, 43.1, 37.1, 31.3, 23.0, 20.9, 20.6, 16.9, 13.8, 9.4, 4.4, 4.3, 3.3, 3.2 ppm; HRMS calcd for C_27_H_36_NO_4_ [M + H]^+^, 438.2639; found, 438.2679.

##### (5α,6*R*,7*R*,14α)-1′-(17-Cyclopropylmethyl-4,5-epoxy-7,8-dihydro-3,6-dimethoxy-7β-methyl-6,14-ethano-morphinan-7-yl)-ethan-1′-ol (**16**)

**15** (0.900 g, 2.057 × 10^–3^ M) was dissolved in EtOH and purged with nitrogen, and Pd (10%) on C (10 mol %) was added. The mixture was degassed under vacuum; then, a hydrogen balloon was applied and it was stirred vigorously. The reaction was checked for completion by MS. When complete, the reaction mixture was filtered, concentrated under vacuum, and could be used without further purification: 0.800 g, 88% yield for the synthesis of **18**. ^1^H NMR (CDCl_3_): δ = 6.71 (d, 1H, *J* = 8.0 Hz), 6.55 (d, 1H, *J* = 8.0 Hz), 5.32 (s, 0.7H), 4.93 (d, 0.7H, *J* = 1.6 Hz), 4.85 (br s, 0.3H), 4.10 (q, 1H, *J* = 6.1 Hz), 3.38–3.88 (m, 3H), 3.89–3.87 (m, 3H), 3.55 (s, 2.2H), 3.45 (s, 0.8H), 3.05–2.94 (m, 2H), 2.66–2.60 (m, 1H), 2.41–2.17 (m, 6H), 1.78–1.70 (m, 1.3H), 1.27–1.23 (m, 4H), 1.21–1.16 (m, 4H), 1.12–1.04 (m, 1H), 0.85–0.76 (m, 2H), 0.49 (t, 2H, *J* = 8.2 Hz), 0.09 (d, 2H, *J* = 4.4 Hz) ppm; ^13^C NMR (CDCl_3_): δ = 146.5, 141.8, 133.3, 128.6, 119.1, 114.3, 93.5, 81.7, 73.3, 59.9, 58.6, 57.0, 53.0, 45.4, 43.8, 42.4, 40.9, 35.7, 33.5, 29.3, 22.6, 17.7, 17.4, 15.3, 9.4, 4.3, 3.2 ppm; HRMS calcd for C_27_H_38_NO_4_ [M + H]^+^, 440.2795; found, 440.2831.

Alternatively, **16a** and **16b** could be separated at this point, giving a 2:1 ratio of the diastereoisomers.

**16a (*R*)**: ^1^H NMR (CDCl_3_): δ = 6.71 (d, 1H, *J* = 8.1 Hz), 6.55 (d, 1H, *J* = 8.1 Hz), 5.29 (s, 1H), 4.94 (d, 1H, *J* = 2.4 Hz), 4.11 (q, 1H, *J* = 6.2 Hz), 3.88 (br s, 3H), 3.55 (s, 3H), 2.99–2.94 (m, 2H), 2.65–2.61 (m, 1H), 2.42–2.35 (m, 2H), 2.31–2.18 (m, 4H), 1.77–1.70 (m, 1H), 1.65–1.58 (m, 3H), 1.33 (br s, 1H) 1.23 (s, 3H), 1.19–1.16 (m, 4H), 1.12–1.04 (m, 1H), 0.85–0.76 (m, 2H), 0.49 (t, 2H, *J* = 7.7 Hz), 0.09 (d, 2H, *J* = 4.8 Hz) ppm; ^13^C NMR (CDCl_3_): δ = 146.5, 141.8, 133.4, 128.6, 119.1, 114.4, 93.5, 81.7, 73.3, 60.0, 58.7, 57.0, 52.9, 45.4, 43.8, 42.5, 40.9, 35.7, 33.6, 29.3, 22.7, 17.7, 17.4, 15.3, 9.4, 4.3, 3.2 ppm.

**16b (*S*)**: ^1^H NMR (CDCl_3_): δ = 6.71 (d, 1H, *J* = 7.5 Hz), 6.55 (d, 1H, *J* = 7.5 Hz), 4.86 (s, 1H), 3.98 (br s, 1H), 3.91–3.86 (m, 3H), 3.46 (s, 3H), 3.04–2.94 (m, 2H), 2.63–2.62 (m, 1H), 2.37–2.23 (m, 4H), 1.76–1.72 (m, 1H), 1.64–1.62 (m, 1H), 1.60–1.57 (m, 3H), 1.50 (d, 1H, *J* = 5.6 Hz), 1.26 (d, 3H, *J* = 6.2 Hz), 1.23–1.21 (m, 1H), 1.16 (s, 3H), 0.82–0.77 (m, 2H), 0.51–0.47 (m, 2H), 0.10–0.07 (m, 2H); ^13^C NMR (CDCl_3_): δ = 145.6, 139.1, 130.3, 123.1, 119.5, 117.8, 91.9, 79.5, 79.0, 64.9, 58.7, 51.8, 43.5, 40.2, 35.4, 26.3, 24.5, 20.4, 18.8, 16.9, 12.8, 7.7, 2.9 ppm.

##### (5α,6*R*,7*R*,14α)-1′-(17-Cyclopropylmethyl-4,5-epoxy-7,8-dihydro-3-hydroxy-6-methoxy-7β-methyl-6,14-ethano-morphinan-7-yl)-ethan-1′-ol (**17**)

**16** (0.700 g, 1.592 × 10^–3^ M) was dissolved in HMPA; to this was added NaH (0.134 g, 5.573 × 10^–3^ M, 3.5 equiv) followed by the addition of propanethiol (0.505 mL, 0.424 g, 5.573 × 10^–3^ M, 3.5 equiv). The mixture was heated to 110 °C for 1–2 h, and the reaction progress was checked by TLC. When complete, the reaction was cooled to RT and the pH was adjusted to 8.0–8.5. The mixture was extracted with diethyl ether (4 × 25 mL), which was in turn washed with deionized water (4 × 25 mL). The organic phase was dried with MgSO_4_, filtered, and concentrated under vacuum. The resultant solids were purified by flash chromatography hexanes to EtOAc, affording 0.381 g, with a 56% yield.

Compound **17b** (from **16b**): ^1^H NMR (CDCl_3_): δ = 6.69 (d, 1H, *J* = 8.1 Hz), 6.50 (d, 1H, *J* = 8.1 Hz), 4.87 (s, 1H), 3.97 (q, 1H, *J* = 6.5 Hz), 3.43 (s, 3H), 3.07 (br s, 1H), 2.94 (d, 1H, *J* = 18.5 Hz), 2.85 (br s, 1H), 2.38–2.25 (m, 6H), 1.76–1.70 (m, 2H), 1.56 (t, 3H, *J* = 9.0 Hz), 1.25 (d, 3H, *J* = 6.4 Hz), 1.22–1.1.16 (m, 1H), 1.14 (s, 3H), 0.86–0.74 (m, 1H), 0.49 (t, 2H, *J* = 6.5 Hz), 0.10 (br s, 2H); ^13^C NMR (CDCl_3_): δ = 145.3, 137.4, 133.4, 128.1, 119.4, 116.4, 95.4, 78.2, 73.1, 60.1, 58.9, 52.4, 45.6, 44.3, 43.8, 39.2, 35.9, 33.4, 29.2, 22.8, 20.9, 17.9, 17.9, 9.4, 4.3, 3.3 ppm; HRMS calcd for C_26_H_35_NO_4_ [M + H]^+^, 426.2639; found, 426.2677.

**17b·HCl**: ^1^H NMR (DMSO-*d*_6_): δ = 9.27 (s, 1H), 8.94 (br s, 1H), 6.70 (d, 1H, *J* = 8.1 Hz), 6.53 (d, 1H, *J* = 8.1 Hz), 4.84 (s, 1H), 4.56 (br s, 1H), 3.83–3.78 (m, 2H), 3.35 (s, 3H), 3.26 (d, 1H, *J* = 19.3 Hz), 3.16–3.13 (m, 1H), 2.95–2.89 (m, 1H), 2.83–2.77 (m, 2H), 2.45–2.40 (m, 1H), 2.35 (d, 1H, *J* = 16.8 Hz), 1.95 (d, 1H, *J* = 13.4 Hz), 1.76 (d, 1H, *J* = 12 Hz), 1.65–1.58 (m, 1H), 1.40–1.31 (m, 1H), 1.08 (d, 3H, *J* = 6.13 Hz), 1.03 (s, 3H), 0.71–0.65 (m, 2H), 0.62–0.54 (m, 2H), 0.37 (sex, 1H, *J* = 4.3 Hz) ppm; ^13^C NMR (DMSO-*d*_6_): δ = 145.8, 139.2, 131.0, 123.0, 119.3, 117.6, 92.2, 76.8, 69.5, 58.6, 57.6, 51.9, 44.7, 43.8, 43.4, 36.8, 35.3, 29.8, 28.5, 24.0, 20.8, 17.5, 17.1, 5.6, 4.8, 2.8 ppm. Purity: 254 nm 96.70% by HPLC, *t*_R_ = 8.5 min, 280 nm 96.90% by HPLC, *t*_R_ = 8.5 min.

Compound **17a** (from **16**a): ^1^H NMR (CDCl_3_): δ = 6.70 (d, 1H, *J* = 7.8 Hz), 6.51 (d, 1H, *J* = 7.8 Hz), 5.34 (br s, 1H), 5.30 (s, 1H), 4.96 (s, 1H), 4.11 (q, 1H, *J* = 6.0 Hz), 3.54 (s, 3H), 3.02–2.93 (m, 2H), 2.63 (d, 1H, *J* = 5.9 Hz), 2.40–2.34 (m, 2H), 2.32–2.18 (m, 4 H), 1.76–1.58 (m, 4H), 1.23 (s, 3H), 1.19–1.16 (m, 3H), 1.12–1.03 (m, 1H), 0.84–0.78 (m, 2H), 0.49 (t, 2H, *J* = 7.7 Hz), 0.90 (d, 1H, *J* = 4.3) ppm; ^13^C NMR (CDCl_3_): δ = 146.6, 141.8, 133.4, 128.6, 119.1, 114.4, 93.5, 81.7, 73.3, 59.9, 58.7, 57.0, 53.0, 45.4, 43.8, 42.4, 40.9, 35.7, 33.6, 29.3, 22.6, 17.7, 17.4, 15.3, 9.4, 4.3, 3.2 ppm. HRMS calcd for C_26_H_35_NO_4_ [M + H]^+^, 426.2639; found, 426.2698.

**17a·HCl**: ^1^H NMR (DMSO-*d*_6_): δ = 9.31 (s, 1H), 9.07 (br s, 1H), 6.72 (d, 1H, *J* = 8.1 Hz), 6.55 (d, 1H, *J* = 8.1 Hz), 5.01 (s, 1H), 3.97 (q, 1H, *J* = 6.2 Hz), 3.81 (d, 1H, *J* = 6.7 Hz), 3.46 (s, 3H), 3.32–3.23 (m, 2H), 3.15 (d, 1H, *J* = 11.6 Hz), 2.99–2.93 (m, 1H), 2.85–2.77 (m, 2H), 2.60 (br s, 1H), 2.41 (t, 1H, *J* = 13.3 Hz), 1.80 (d, 1H, *J* = 12.3 Hz), 1.64–1.47 (m, 2H), 1.36 (d, 1H, *J* = 13.4 Hz), 1.32–1.23 (m, 1H), 1.15 (s, 3H), 1.11–1.09 (m, 1H), 1.06 (d, 3H, *J* = 6.0 Hz), 0.73–0.63 (m, 2H), 0.62–0.52 (m, 2H), 0.36 (sex, 1H, *J* = 4.5 Hz) ppm; ^13^C NMR (DMSO-*d*_6_): δ = 145.5, 139.3, 132.0, 130.6, 123.0, 119.6, 117.8, 90.5, 80.39, 72.2, 64.8, 58.3, 57.6, 52.5, 44.5, 43.5, 35.3, 29.9, 28.5, 24.0, 17.6, 17.2, 15.6, 15.1, 5.6, 4.8, 2.9 ppm; purity: 254 nm 98.30% by HPLC, *t*_R_ = 8.4 min, 280 nm 99.24% by HPLC, *t*_R_ = 8.4 min.

##### (5α,6*R*,7*R*,14α)-1′-(1-Cyclopropylmethyl-4,5-epoxy-7,8-dihydro-3,6-dimethoxy-7β-methyl-6,14-ethano-morphinan-7-yl)-ethanone (**18**)

**16** (0.732 g, 1.665 × 10^–3^ M) was dissolved in DCM; to this were added potassium carbonate (0.230 g, 1.665 × 10^–3^ M 1.0 equiv) and then DMPI (1.059 g, 2.498 × 10^–3^ M, 1.5 equiv). The mixture was stirred for 24 h and checked for progress by TLC. When complete, the mixture’s pH was adjusted to 8.0–8.5 and it was washed with water. The organic layer was retained and dried with MgSO_4_, then filtered, and concentrated under vacuum. The resultant waxy solid was purified by flash chromatography hexanes to EtOAc, affording 0.550 g, with a 75% yield. ^1^H NMR (CDCl_3_): δ = 6.70 (d, 1H, *J* = 8.0 Hz), 6.55 (d, 1H, *J* = 8.0 Hz), 4.84 (d, 1H, *J* = 1.9 Hz), 3.88 (s, 3H), 3.46 (s, 3H), 3.14 (d, 1H, *J* = 6.3 Hz), 2.95 (d, 1H, *J* = 18.3 Hz), 2.59 (d, 1H, *J* = 7.0 Hz), 2.45–2.37 (m, 2H), 2.32–2.24 (m, 6H), 2.21–2.16 (m, 1H), 2.12 (dd, *J* = 13.5, *J* = 3.7 Hz), 1.64–1.54 (m, 3H), 1.51 (s, 3H), 1.39–1.31 (m, 1H), 0.98 (t, 1H, *J* = 12.3 Hz), 0.81–0.74 (m, 1H), 0.68 (t, 1H, *J* = 12.0 Hz), 0.53–0.44 (m, 2H), 0.09 (d, 2H, *J* = 4.5 Hz) ppm; ^13^C NMR (CDCl_3_): δ = 213.3, 146.5, 141.8, 133.5, 128.8, 119.1, 114.3, 94.0, 60.0, 58.5, 57.0, 54.5, 52.8, 45.8, 43.9, 37.0, 35.7, 33.6, 29.1, 27.9, 22.7, 21.7, 18.4, 9.4, 4.5, 3.0 ppm; HRMS calcd for C_27_H_36_NO_4_ [M + H]^+^, 438.2639; found, 438.2686.

##### (5α,6*R*,7*R*,14α)-2′-(17-Cyclopropylmethyl-4,5-epoxy-7,8-dihydro-3,6-dimethoxy-7β-methyl-6,14-ethano-morphinan-7-yl)-propan-2′-ol (**19**)

**18** (0.550 g, 1.257 × 10^–3^ M) was dissolved in toluene and cooled to −78 °C; to this was cautiously added MeLi [1.6 M] (1.6 mL, 2.514 × 10^–3^ M, 2.0 equiv); this was allowed to warm to RT and stirred for 24 h. The excess MeLi was destroyed by the careful addition of IPA followed by water. The pH was adjusted to 8.0–8.5, and the organic phase was separated, dried with MgSO_4_, and concentrated under vacuum. This was used without further purification, affording 0.448 g, with a 79% yield. ^1^H NMR (CDCl_3_): δ = 6.70 (d, 1H, *J* = 8.0 Hz), 6.54 (d, 1H, *J* = 8.0 Hz), 5.03 (d, 1H, *J* = 2.3 Hz), 3.89 (s, 3H), 3.52 (s, 3H), 3.50–3.48 (m, 1H), 3.03 (d, 1H, *J* = 6.3 Hz), 2.97 (d, 1H, *J* = 18.3 Hz), 2.61 (dd, 1H, *J* = 11.6 Hz, *J* = 5.5 Hz), 2.50–2.38 (m, 3H), 2.32–2.19 (m, 3H), 1.89 (t, 1H, *J* = 12.7 Hz), 1.79–1.70 (m, 1H), 1.59–1.51 (m, 3H), 1.49 (s, 3H), 1.41 (s, 3H), 1.41–1.38 (m, 1H), 1.27 (s, 3H), 1.25–1.16 (m, 1H), 0.84–0.78 (m, 2H), 0.50 (t, 2H, *J* = 8.7 Hz), 0.10 (dd, 2H, *J* = 5.0, *J* = 1.6 Hz) ppm; ^13^C NMR (CDCl_3_): δ = 146.8, 141.7, 133.9, 128.9, 118.9, 114.4, 94.4, 82.2, 78.5, 60.0, 58.9, 57.1, 52.6, 46.5, 45.9, 43.9, 39.2, 36.5, 33.4, 30.5, 29.2, 27.9, 22.8, 21.2, 19.0, 9.4, 4.4, 3.2 ppm; HRMS calcd for C_28_H_39_NO_4_ [M + H]^+^, 454.2952; found, 454.2967.

##### (5α,6*R*,7*R*,14α)-2′-(17-Cyclopropylmethyl-4,5-epoxy-7,8-dihydro-3-hydroxy-6-methoxy-7β-methyl-6,14-ethano-morphinan-7-yl)-propan-2′-ol (**20**)

**19** (0.448 g, 9.876 × 10^–4^ M) was dissolved in HMPA (2 mL); to this was added NaH (0.083 g, 3.457 × 10^–3^ M, 3.5 equiv) followed by the addition of propanethiol (0.313 mL, 0.263 g, 3.457 × 10^–3^ M, 3.5 equiv). The mixture was heated to 110 °C for 1–2 h, and the reaction progress was checked by TLC. When complete, the reaction was cooled to RT, and its pH was adjusted to 8.0–8.5. The mixture was extracted with diethyl ether (4 × 25 mL), which was in turn washed with deionized water (4 × 25 mL). The organic phase was dried with MgSO_4_, filtered, and concentrated under vacuum. The resultant solids were purified by flash chromatography with hexanes to EtOAc, affording 0.293 g, with a 67% yield. **20·HCl** was prepared as described above. ^1^H NMR (DMSO-*d*_6_): δ = 9.22 (s, 1H), 8.89 (br s, 1H), 6.68 (d, 1H, *J* = 8.1 Hz), 6.52 (d, 1H, *J* = 8.1 Hz), 4.90 (s, 1H), 4.42 (s, 1H), 3.77 (d, 1H, *J* = 6.7 Hz), 3.41–3.36 (m, 1H), 3.34 (s, 3H), 3.25 (d, 1H, *J* = 19.4 Hz) 3.15 (d, 1H, *J* = 9.7 Hz), 2.98–2.92 (m, 1H), 2.87–2.85 (m, 2H), 2.45–2.37 (m, 2H) 2.14–2.03 (m, 2H), 1.76 (d, 1H, *J* = 15.1 Hz), 1.62–1.54 (m, 1H), 1.36–1.23 (m, 4H), 1.14 (d, 6H, *J* = 4.5 Hz), 1.09 (t, 1H, *J* = 7.2 Hz) 0.72–0.65 (m, 1H), 0.63–0.51 (m, 3H), 0.41–0.35 (m, 1H) ppm. ^13^C NMR (DMSO-*d*_6_): δ = 203.7, 190.2, 146.0, 139.2, 135.7, 131.3, 127.4, 123.2, 119.2, 117.5, 92.8, 78.8, 76.0, 58.9, 57.6, 51.1, 46.0, 44.7, 43.4, 35.7, 29.9, 27.8, 24.1, 23.1, 21.2, 18.3, 17.6, 5.6, 4.8, 2.8 ppm; HRMS calcd for C_27_H_38_NO_4_ [M + H]^+^, 440.2795; found, 440.2812; purity: 254 nm 100.00% by HPLC, *t*_R_ = 9.3 min, 280 nm 97.85% by HPLC, *t*_R_ = 9.3 min.

### Biological Studies

#### In Vitro

##### Cell Culture

Chinese hamster ovary (CHO) cells expressing human (h) MOR, KOR, or DOR were grown in 50:50 DMEM/F12 media with 10% FBS, 0.5% penicillin/streptomycin, and 400 μg/mL G418 (all Gibco) in a 37 °C humidified incubator at 5% CO_2_. Cells were harvested at 85–90% confluency with 0.5 mM EDTA, 150 mM NaCl, 20 mM HEPES pH 7.4, resuspended in 50 mM Tris HCl pH 7.4, and homogenized using a tissue grinder on ice. Homogenates were centrifuged at 15,000*g* at 4 °C for 30 min, washed, and stored at −80 °C.

##### Radioligand Binding

CHO membrane homogenates (10–20 μg protein) expressing hMOR, hKOR, or hDOR were incubated with 0.2–0.5 nM ^3^H-diprenorphine (PerkinElmer) and varying concentrations of test ligands at 25^o^C for 1 h, followed by termination by rapid filtration through 96-well GF/B filter plates (PerkinElmer). Plates were washed with ice-cold 50 mM Tris HCl pH 7.4 buffer, dried, and 40 μL of MicroScint-PS scintillation cocktail (PerkinElmer) added. Bound radioactivity was measured using a MicroBeta2450 scintillation counter (PerkinElmer). Assays were performed on at least three separate occasions in duplicate. Data were analyzed to provide Ki values as a measure of receptor affinity using GraphPad Prism, v. 8.0.

##### [^35^S]-GTPγS Binding

CHO homogenates (10–20 μg protein) expressing hMOR, hKOR, or hDOR were incubated with 0.1 nM [^35^S]-GTPγS (PerkinElmer) in 50 mM Tris HCl pH 7.4, 100 mM NaCl, 5 mM MgCl_2_, 1 mM EDTA, and 30 μM GDP for 60 min at 25 °C. Reactions were terminated by vacuum filtration as described above. Filters were dried, and bound [^35^S]-GTPγS was measured as described above. Assays were performed on at least three separate occasions in duplicate. Data were analyzed to provide potency (EC50) values and relative efficacy values as % maximal effect compared to standard agonists DAMGO (MOR), U69593, KOR, and SNC-80 (DOR) using GraphPad Prism, v. 8.0.

#### In Vivo

##### Animals

Male and female C57/BL6 and CD1 mice bred in-house and weighing between 25 and 40 g at 6–8 weeks old were used for behavioral experiments. Mice were group-housed with a maximum of five animals per cage in clear polypropylene cages with corn cob bedding and nest-lets as enrichment. Mice had free access to food and water at all times. Animals were housed in pathogen-free rooms maintained at 71 ± 2 °F and between 30 and 0% humidity with a 12 h light/dark cycle with lights on at 7:00 AM. Experiments were conducted in a procedure room during the light cycle. Each mouse was used in only one experiment. Studies were performed in accordance with the US National Research ’Council’s Guide for the Care and Use of Laboratory Animals^18^ and the ARRIVE guidelines.^19^

##### Drug Preparation

All compounds were administered by intraperitoneal (i.p.) injection in a volume of 10 mL/kg of body weight. Fentanyl HCl (NIDA drug supply), BU72 (synthesized as previously described^20^), **14**, and naloxone HCl (NLX; Tocris, Biosciences, Minneapolis, MN, USA) were dissolved in sterile saline (0.9% NaCl w/v).

##### Antinociceptive Assay—Warm Water Tail Withdrawal Test

Experiments were performed on C57BL/6 wild-type mice (Jackson Laboratory). The distal tip of the mouse tail (∼1/3) was placed in a 50 °C warm-water bath, and the latency for the mouse to flick its tail was measured.^21^ A maximum cutoff time of 20 s was implemented to prevent tissue damage. BU72, naloxone, and **14** were administered i.p. Tail-withdrawal latencies were measured at the indicated times. Antinociception was expressed as a percentage of maximum possible effect (% MPE), where % MPE = (drug latency – baseline latency)/(cutoff latency – baseline latency) × 100. Data were analyzed by two-way ANOVA, followed by Tukey’s posthoc test.

##### Fentanyl-Induced Respiratory Depression

A MouseOx Plus system from Starr Life Sciences was used to measure pulse oximetry in awake freely moving CD1 mice. Enclosures contained corn cob bedding and access to DietGel. Mice were habituated to the enclosures for 1 h wearing a dummy collar. The dummy collar was then removed and replaced with the MouseOx collar (size small). Baseline measurements of percent oxygen saturation (spO_2_) were recorded for 1 h. At *t* = 0, mice were injected with 10 mg/kg fentanyl i.p.; at *t* = 30, either naloxone or compound **14** was administered i.p. Data was recorded until spO_2_ returned to the baseline and averaged into 5 min bins. Data were recorded at a rate of 1 Hz, with five data points collected before moving on to the next subject.

## Supporting Information Available

NMR and mass spectra for all compounds and HPLC traces for final products (PDF)

## Abbreviations

MOR: mu-opioid receptor
KOR: kappa-opioid receptor
DOR: delta-opioid receptor
CHO: Chinese hamster ovary
