# Supplementary material for: Opioid Antagonists from the Orvinol Series as Potential Reversal Agents for Opioid Overdose
Source: ACS Chem Neurosci. 2022 Oct 12;13(21):3108–17. doi: 10.1021/acschemneuro.2c00464 (PMC9634796; doi:10.1021/acschemneuro.2c00464)
Supplement: Supplementary file 1 — cn2c00464_si_001.pdf [file cn2c00464_si_001.pdf]

Supporting information:

Opioid Antagonists from the Orvinol Series as Potential Reversal Agents for Opioid Overdose

Alex Disney[1*], Keith M. Olson[2*], Amanda M. Shafer[2], Sierra C. Moore[2], Jessica P. Anand[2], John R. Traynor[2,3,] and Stephen M. Husbands[1]

[1]Medicinal Chemistry Section, Department of Life Sciences, University of Bath, Bath, BA2 7AY, United Kingdom

[2]Department of Pharmacology and Edward F Domino Research Center, University of Michigan, Ann Arbor, MI 48109 USA

[3]Department of Medicinal Chemistry, University of Michigan, Ann Arbor, MI 48109 USA

Compound 4a + 4b unseparated reaction products (Showing ratio of aldehyde protons, **4b**:**4a**, 2:1)

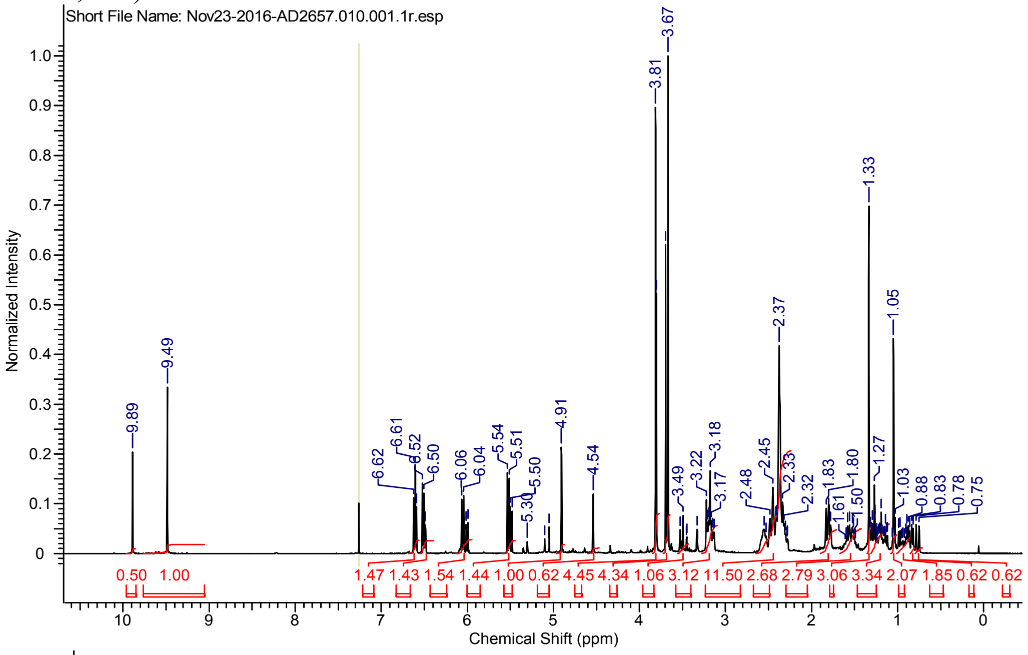

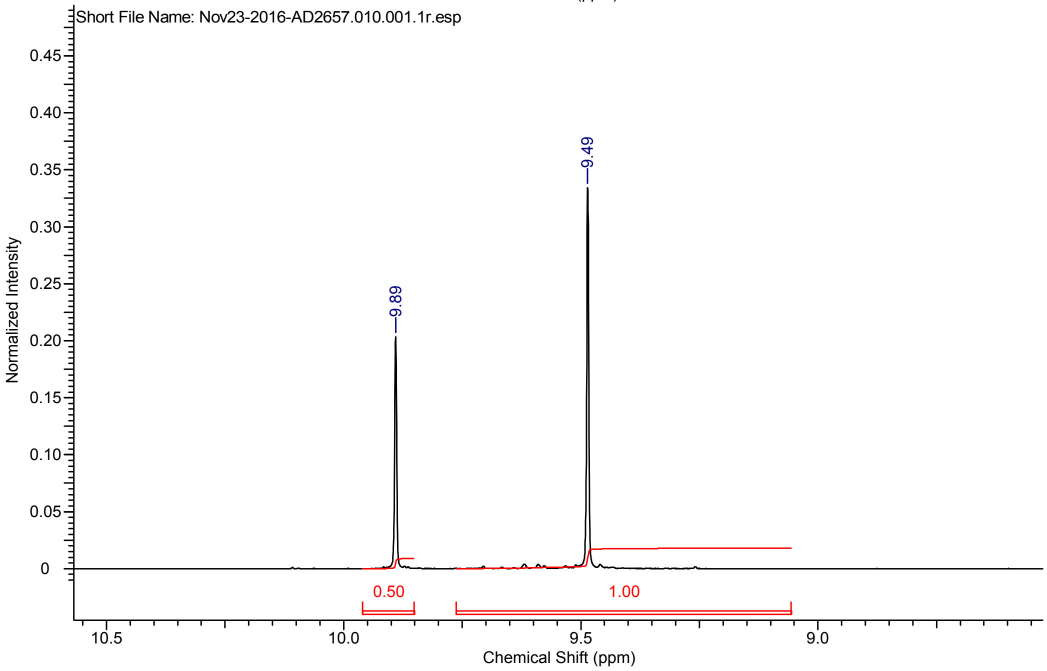

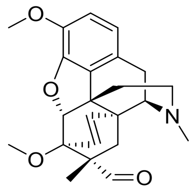

4,5-Epoxy-3,6-dimethoxy-7β,17-dimethyl-6,14-ethenomorphinan-7α-carboxaldehyde (4b)

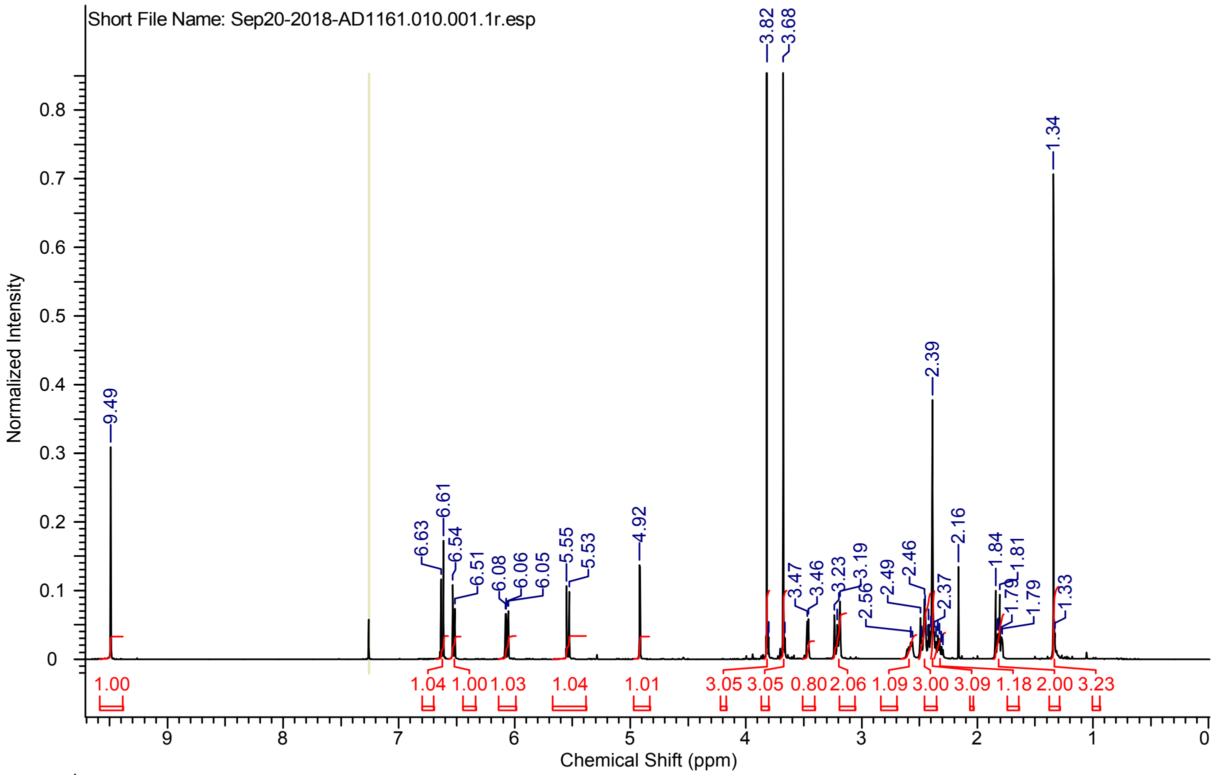

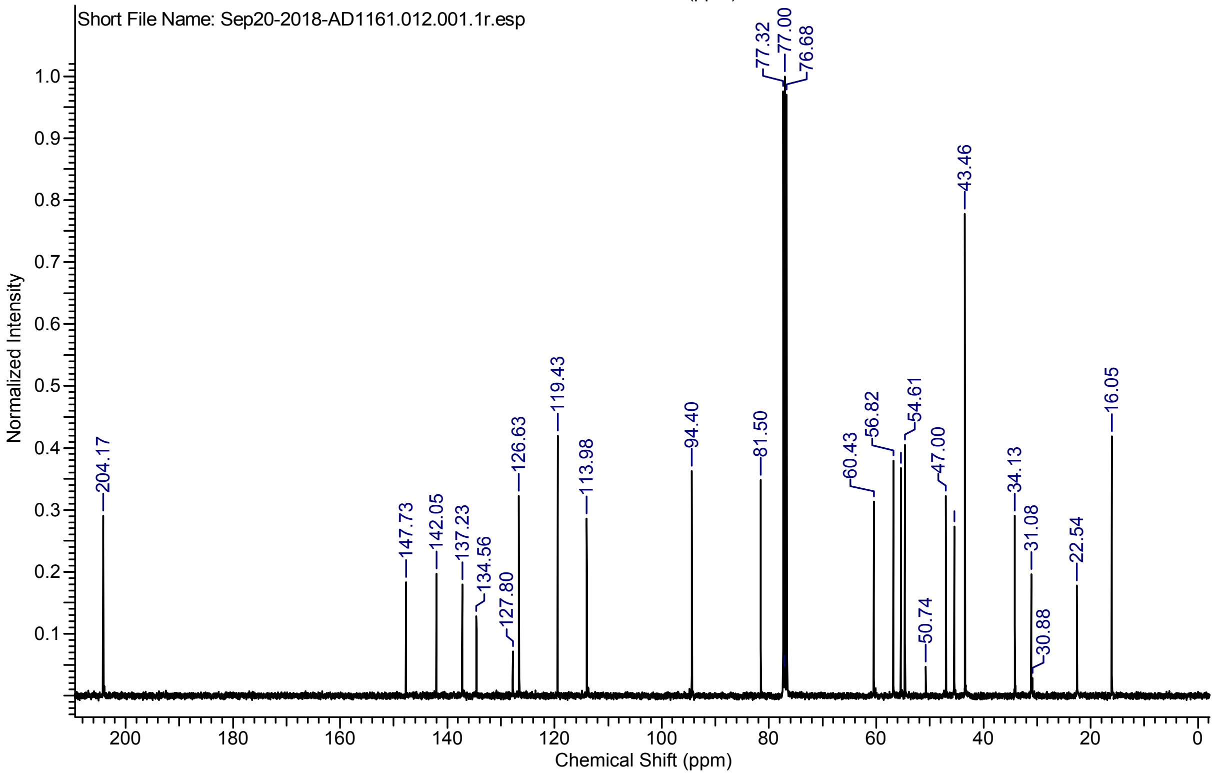

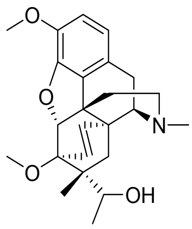

(5α,6R,7R,14α)-1′-(4,5-epoxy-7,8-dihydro-3,6-dimethoxy-7β,17-dimethyl-6,14-etheno-morphinan-7-yl)-ethan-1′-ol (5)

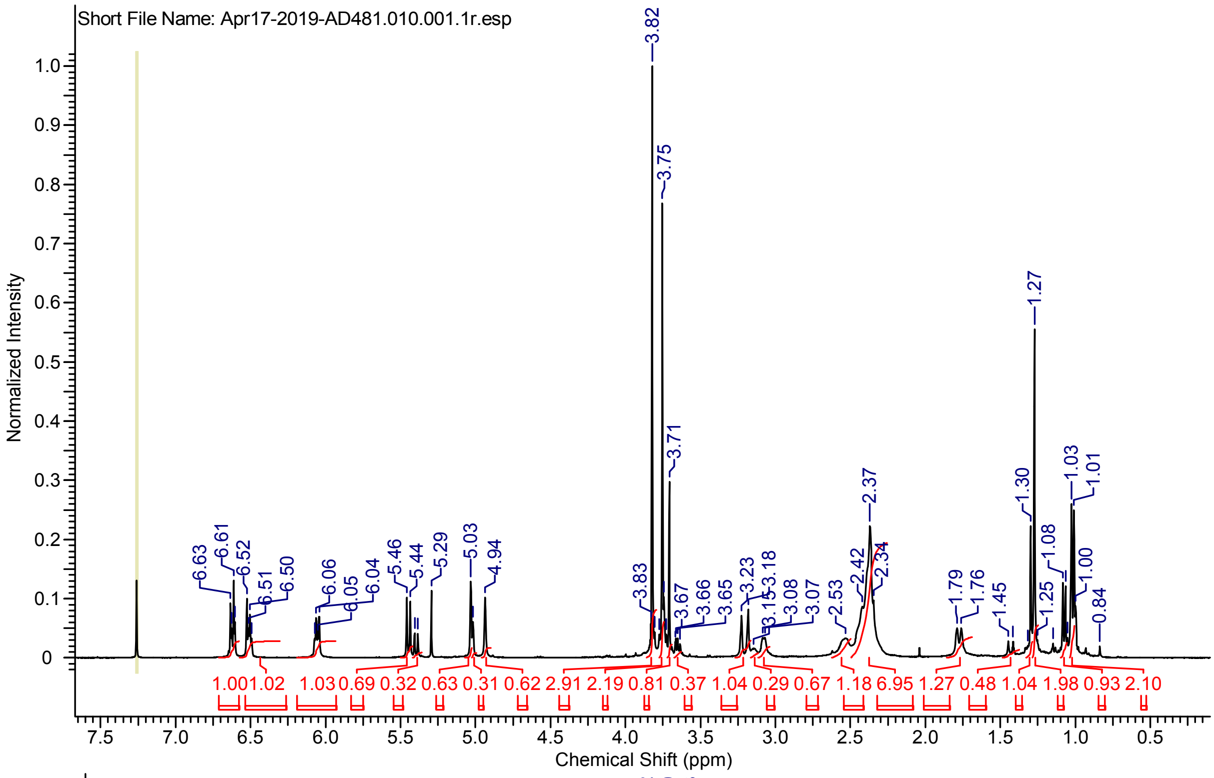

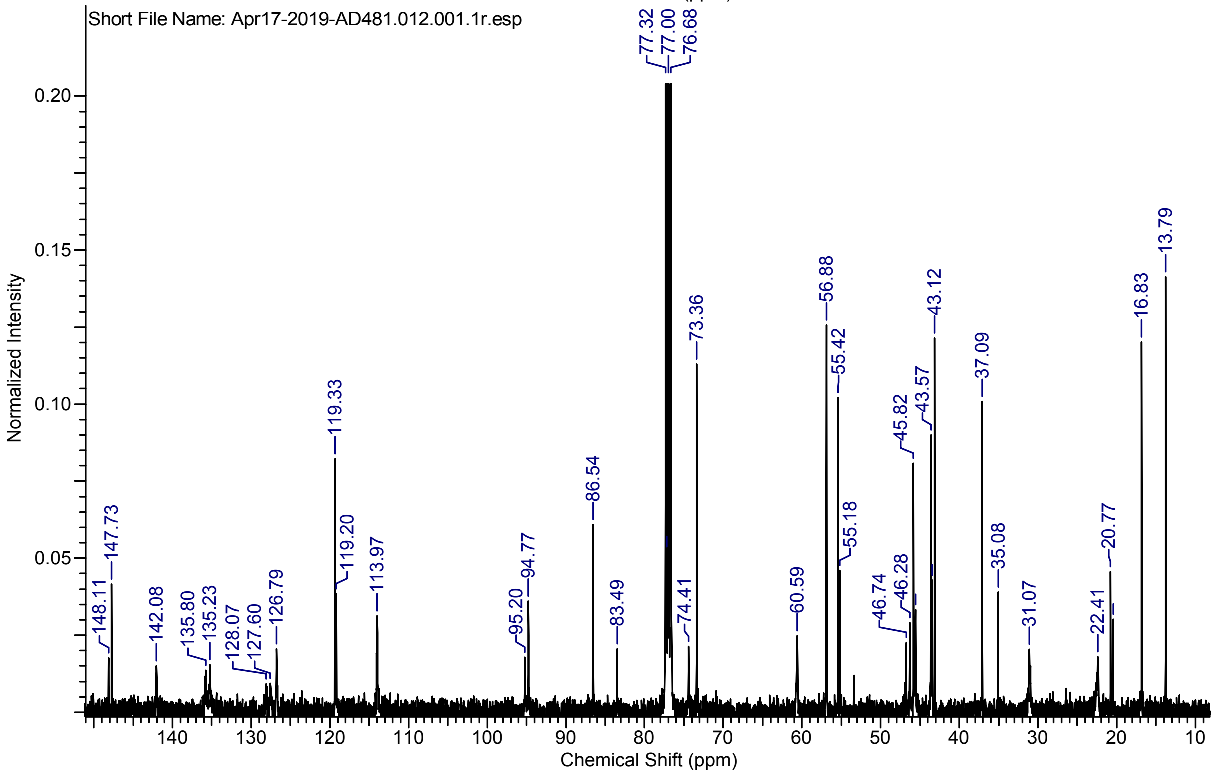

(5α,6R,7R,14α)-1′-(4,5-epoxy-7,8-dihydro-3,6-dimethoxy-7β,17-dimethyl-6,14-ethano-morphinan-7-yl)-ethan-1′-ol (6)

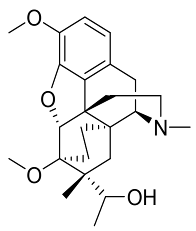

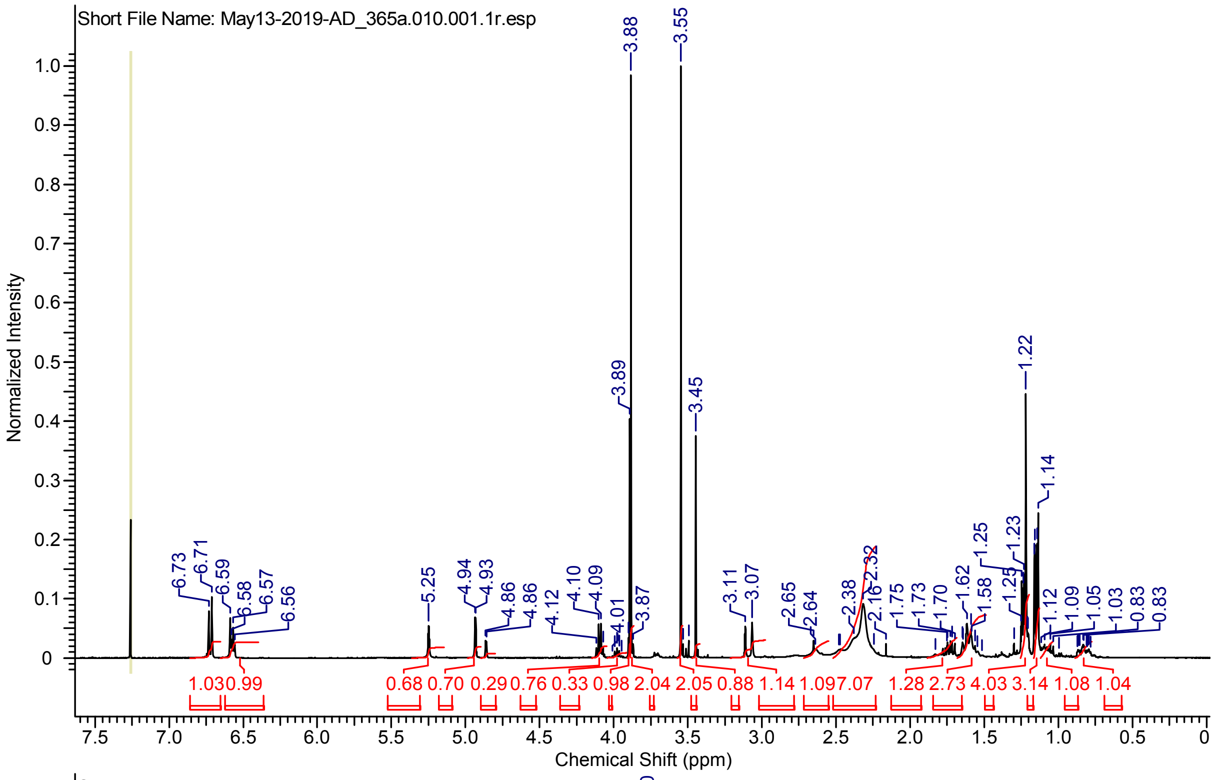

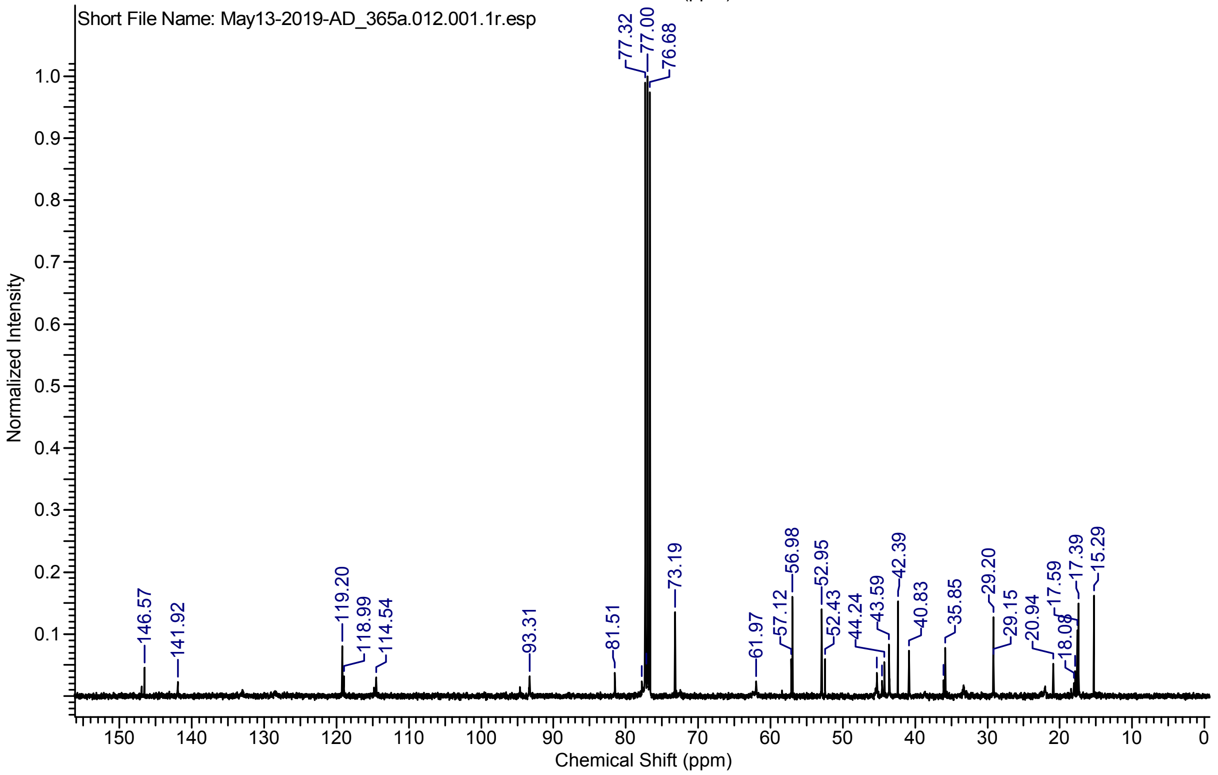

(5α,6R,7R,14α)-1′-(4,5-epoxy-7,8-dihydro-3,6-dimethoxy-7β,17-dimethyl-6,14-ethano-morphinan-7-yl)-ethanone (7)

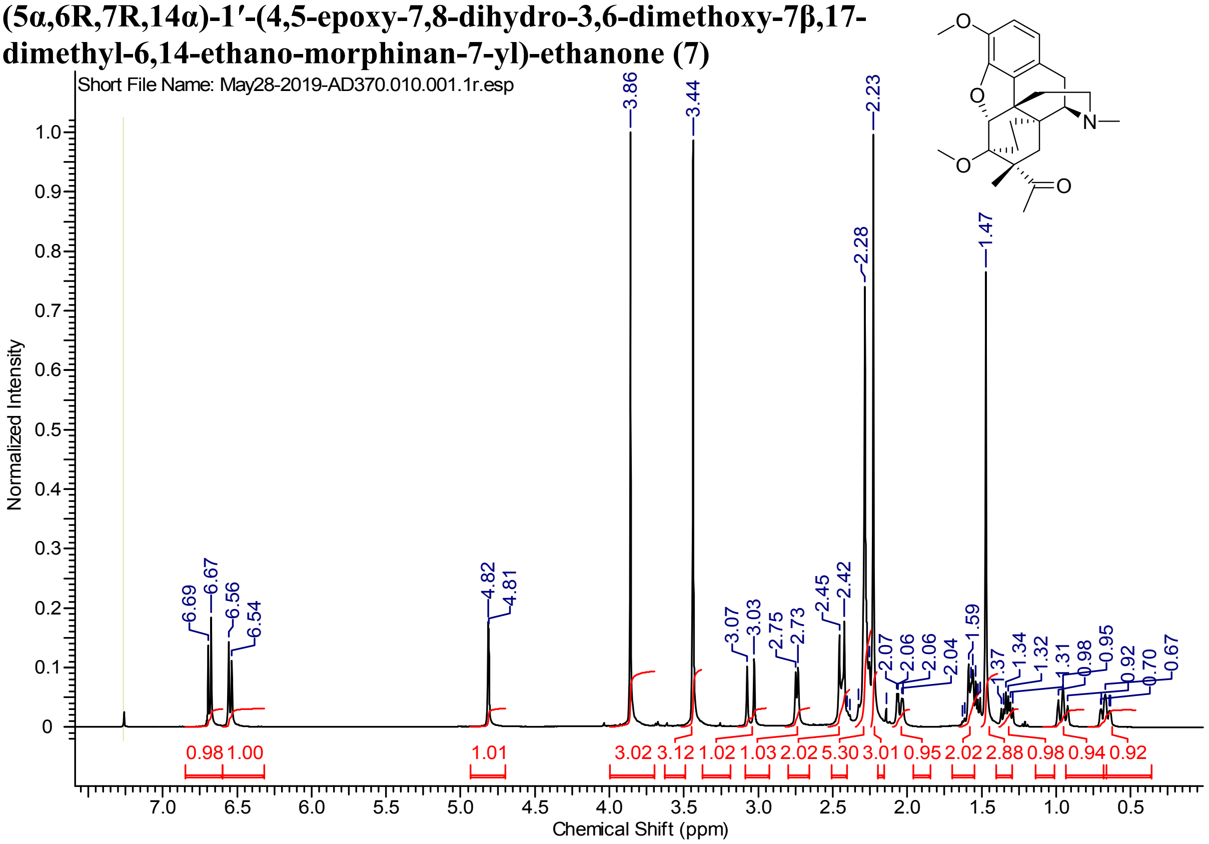

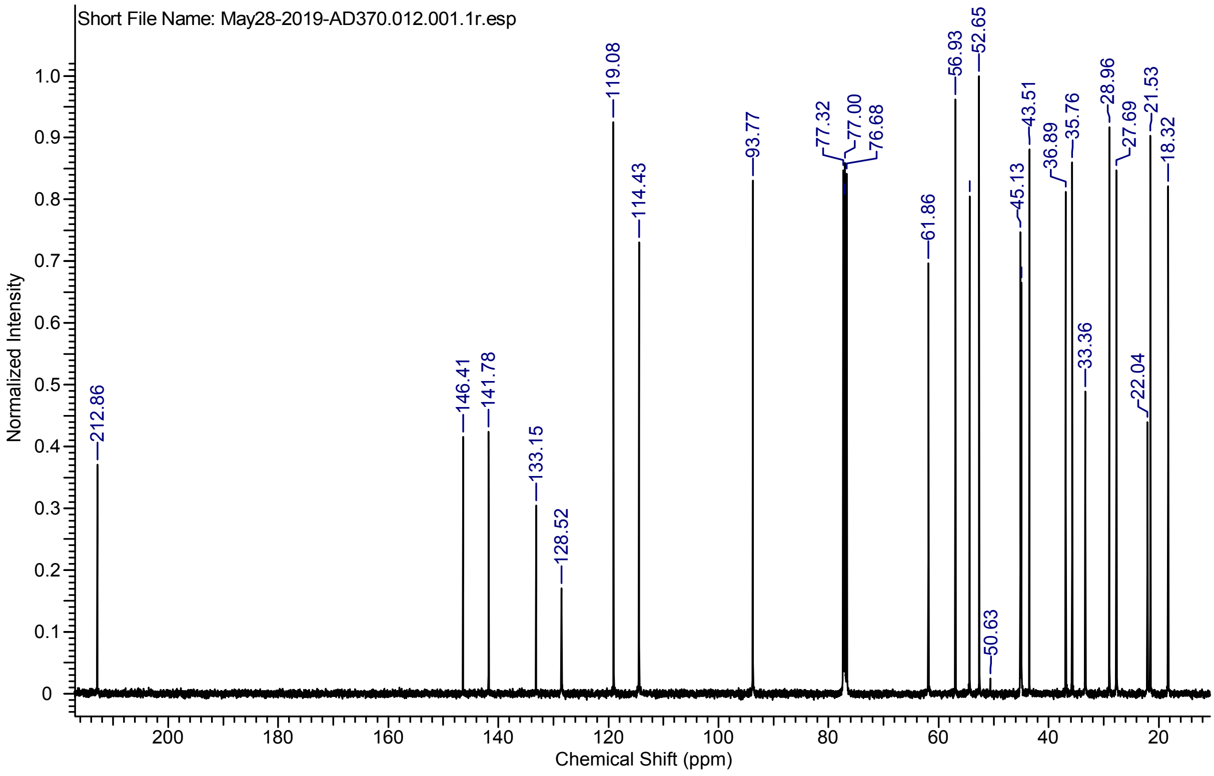

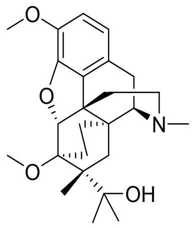

(5α,6R,7R,14α)-2′-(4,5-epoxy-7,8-dihydro-3,6-dimethoxy-7β,17-dimethyl-6,14-ethano-morphinan-7-yl)-propan-2′-ol (8)

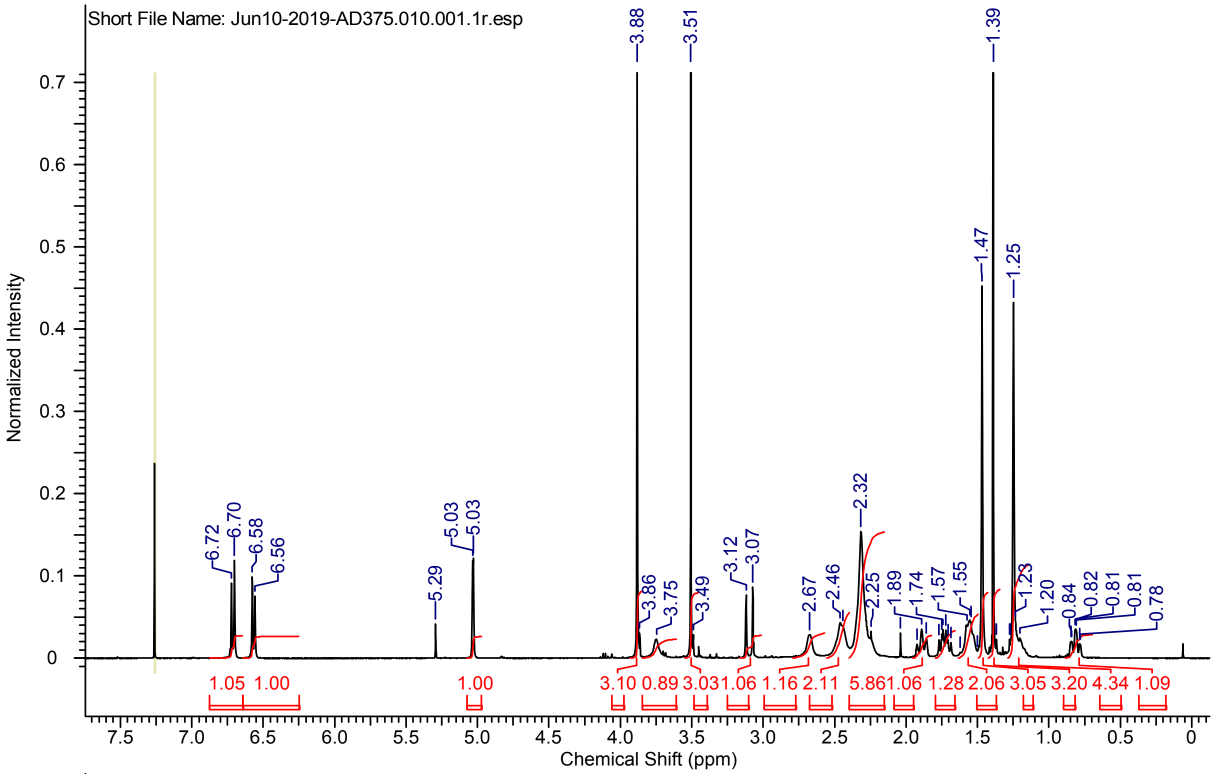

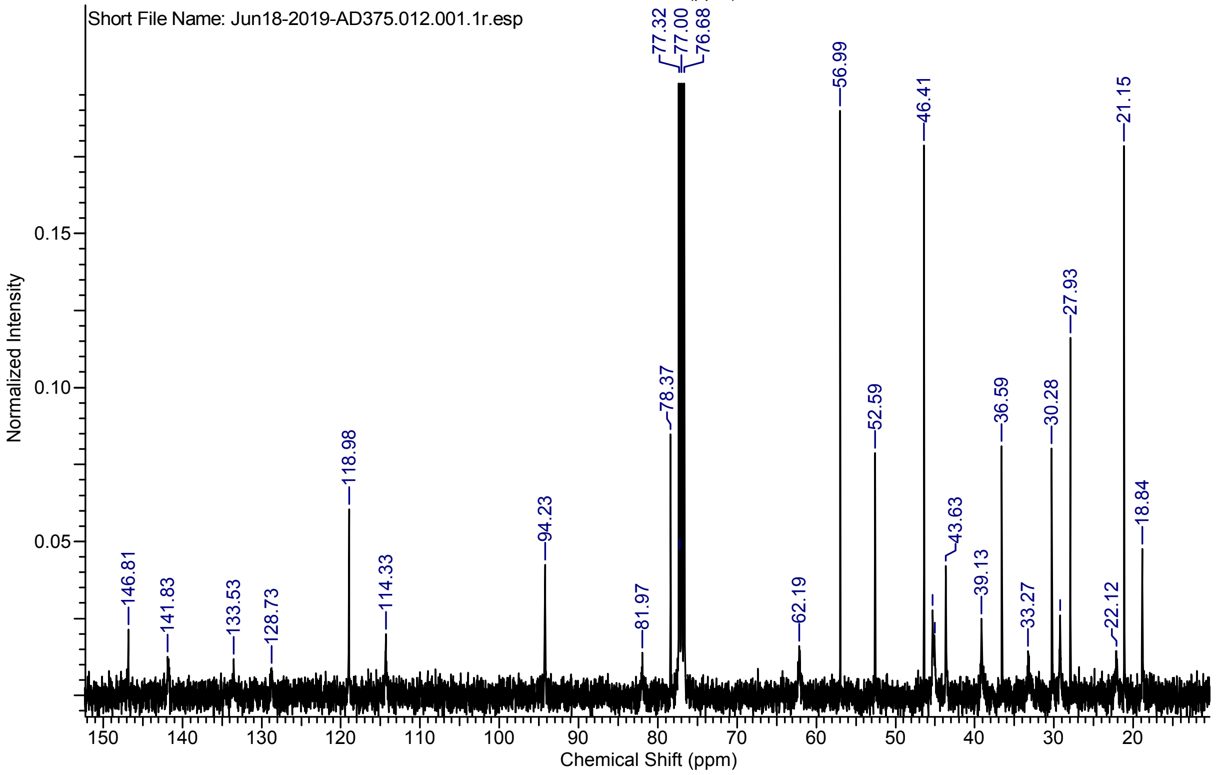

(5α,6R,7R,14α)-2′-(4,5-epoxy-7,8-dihydro-3-hydroxy-6-methoxy-7β,17-dimethyl-6,14-ethano-morphinan-7-yl)-propan-2′-ol.HCl (9.HCl)

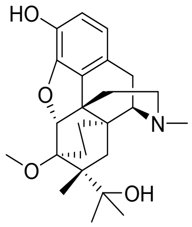

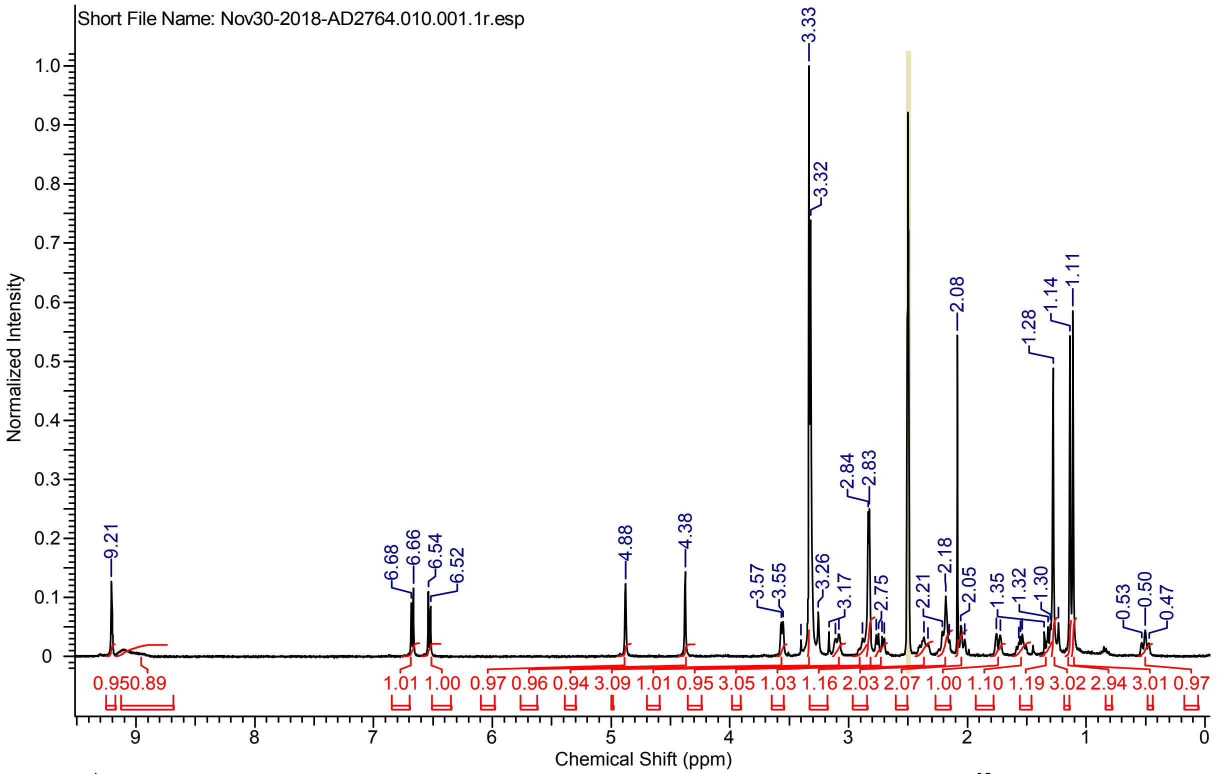

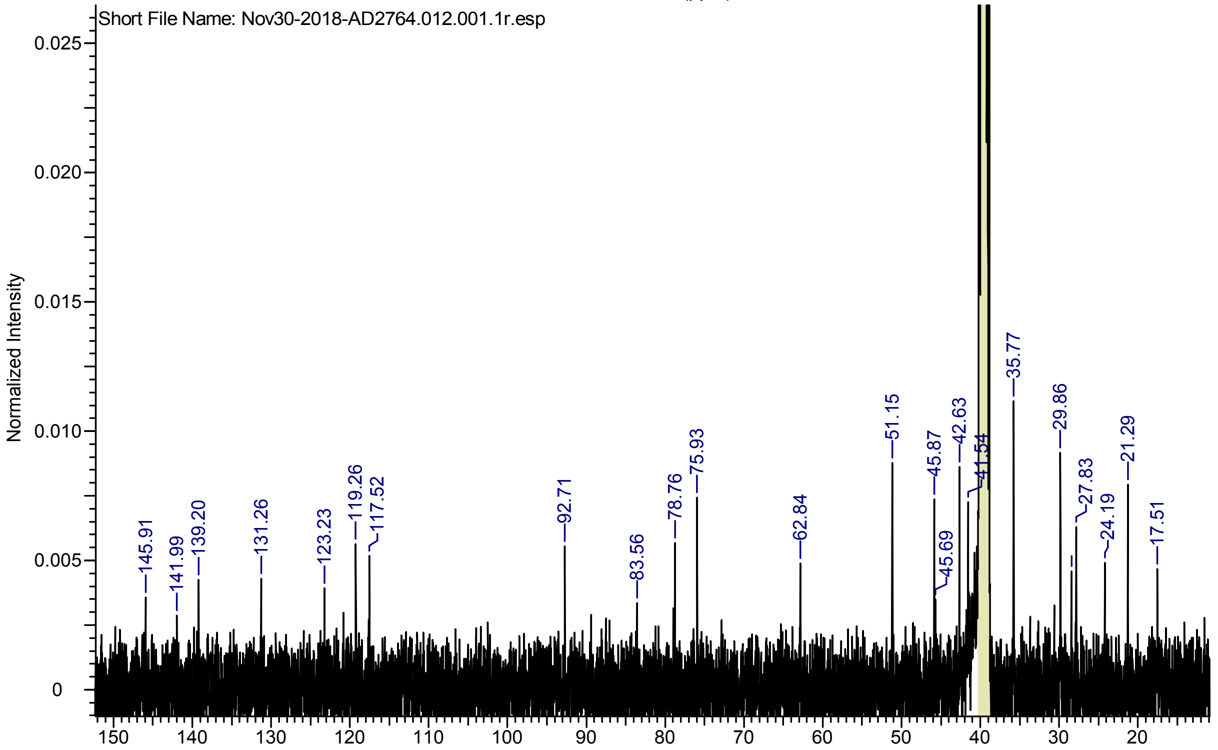

HPLC of **9** at 254 nm

Chromatogram Report

**Analysis Info**		Acquisition Date	<illegal time>
Analysis Name	C:\Data\2019-01-04 Alex RP C18\AD336 HCl_RB5_01_4181.d		
Method		Operator	
Sample Name		Instrument	
Comment			

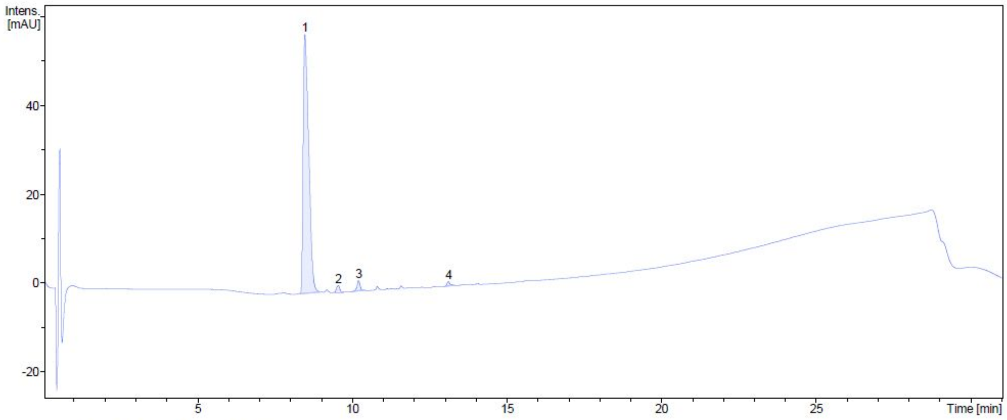

#	RT [min]	Area	Area Frac. %
1	8.5	733.1425	95.80
2	9.5	11.5746	1.51
3	10.2	14.3741	1.88
4	13.1	6.1571	0.80

Bruker Compass DataAnalysis 4.3 printed: 07-Jan-19 12:22:24 PM by: bdal 1 of 1

HPLC of **9** at 280 nm

Chromatogram Report

**Analysis Info**		Acquisition Date	<illegal time>
Analysis Name	C:\Data\2019-01-04 Alex RP C18\AD336 HCl_RB5_01_4181.d		
Method		Operator	
Sample Name		Instrument	
Comment			

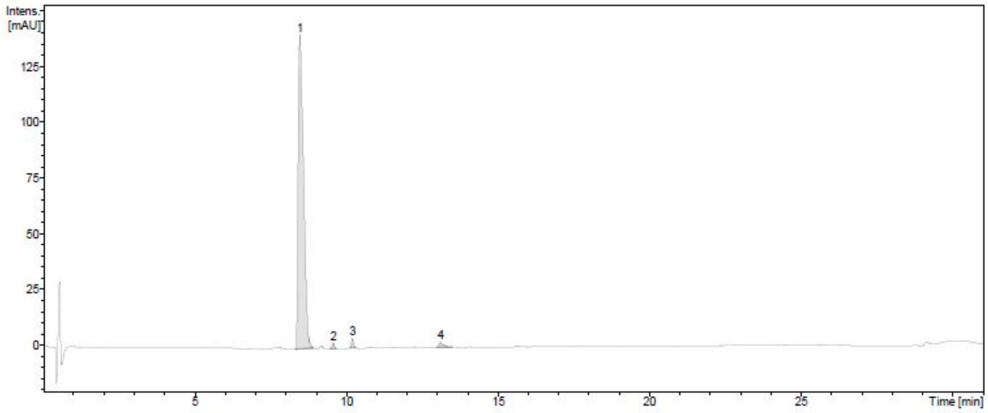

#	RT [min]	Area	Area Frac. %
1	8.5	1730.625	96.76
2	9.6	14.331	0.80
3	10.2	20.142	1.13
4	13.1	23.443	1.31

Bruker Compass DataAnalysis 4.3 printed: 07-Jan-19 12:24:32 PM by: bdal 1 of 1

4,5-Epoxy-3,6-dimethoxy-7β-methyl-6,14-ethenomorphinan-7α-carboxaldehyde.HCl (10.HCl)

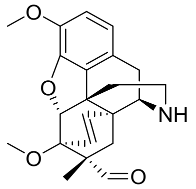

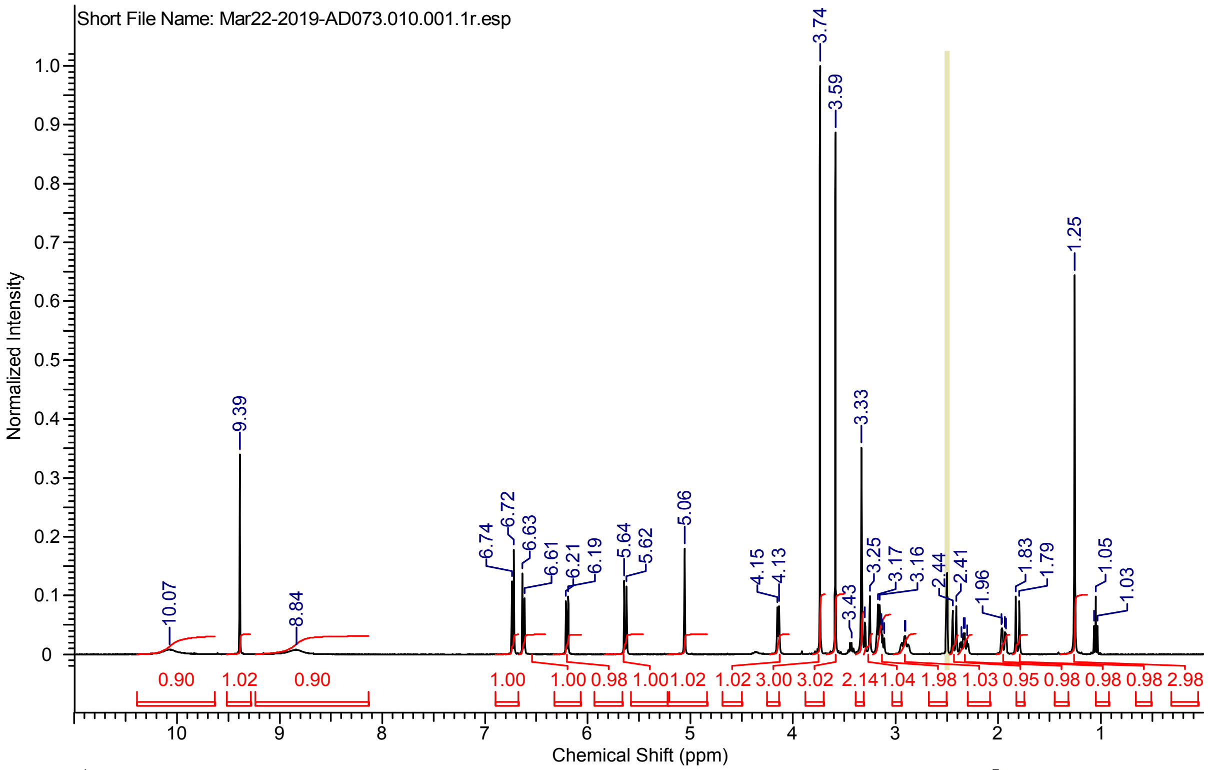

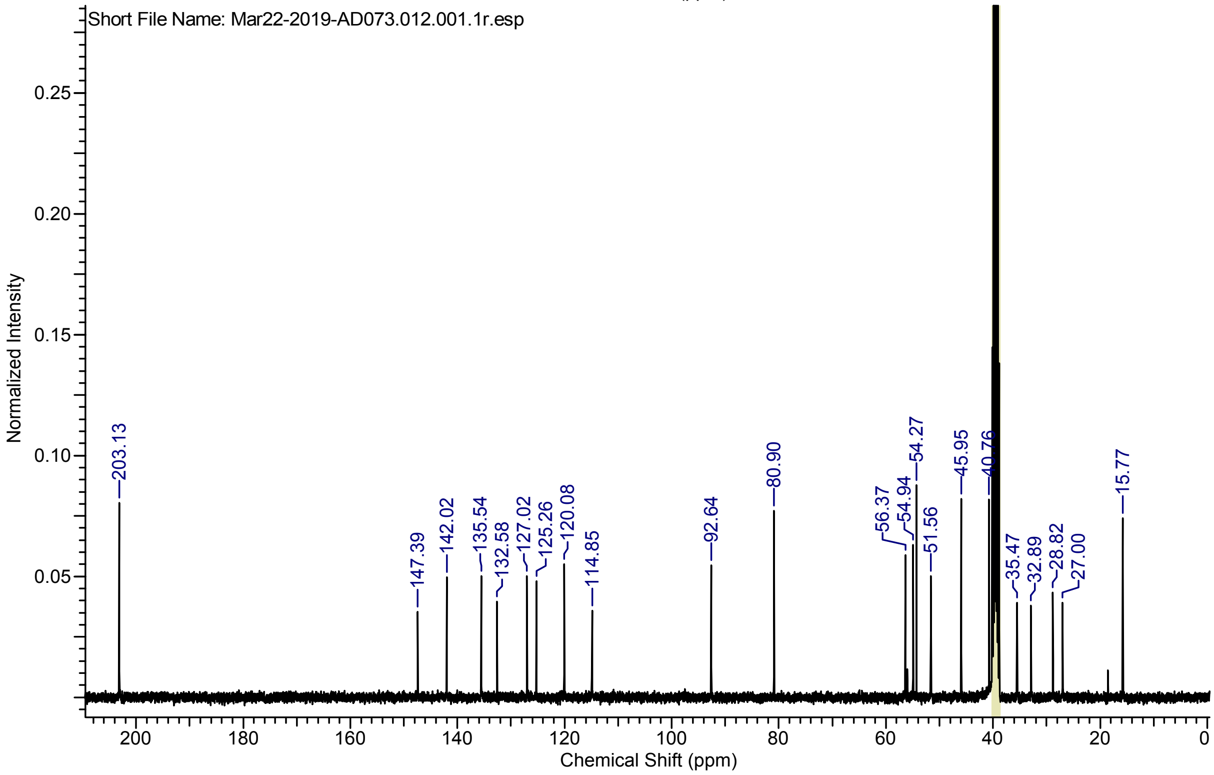

17-Cyclopropylmethyl-4,5-epoxy-3,6-dimethoxy-7β-methyl-6,14-ethenomorphinan-7α-carboxaldehyde (11)

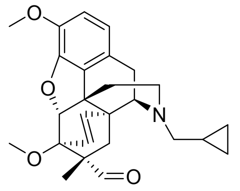

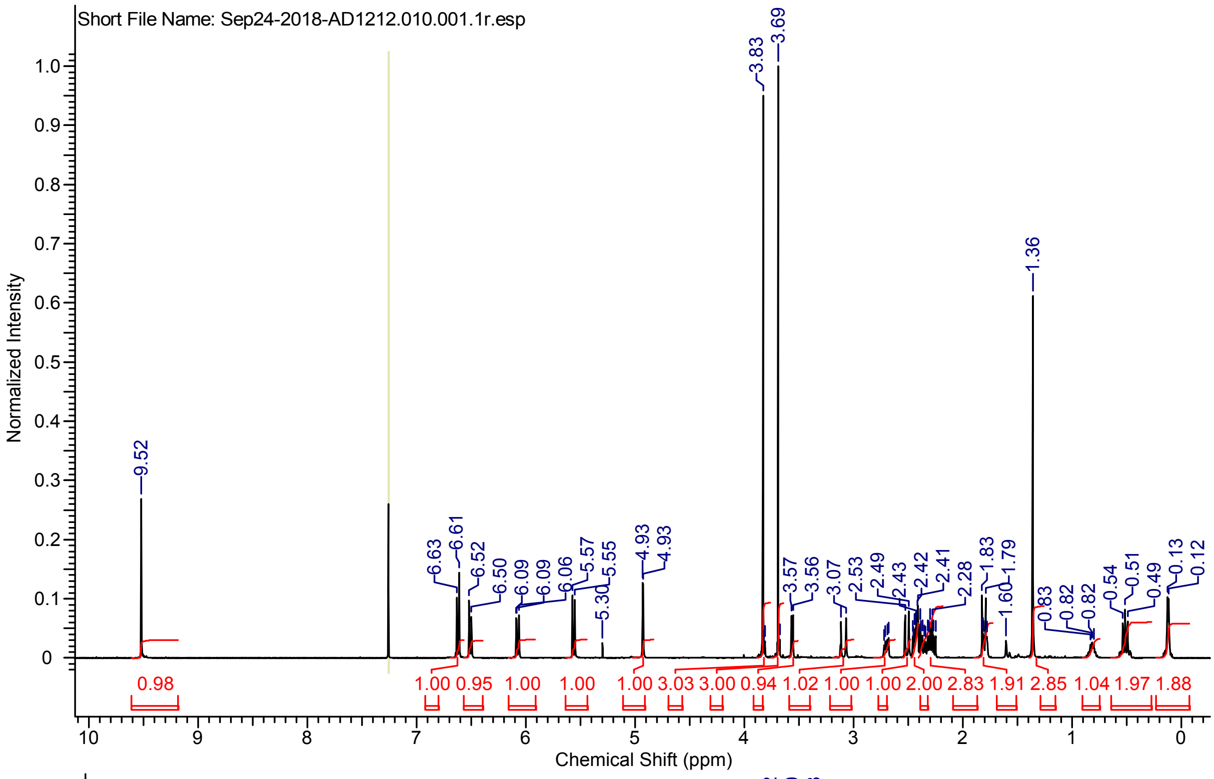

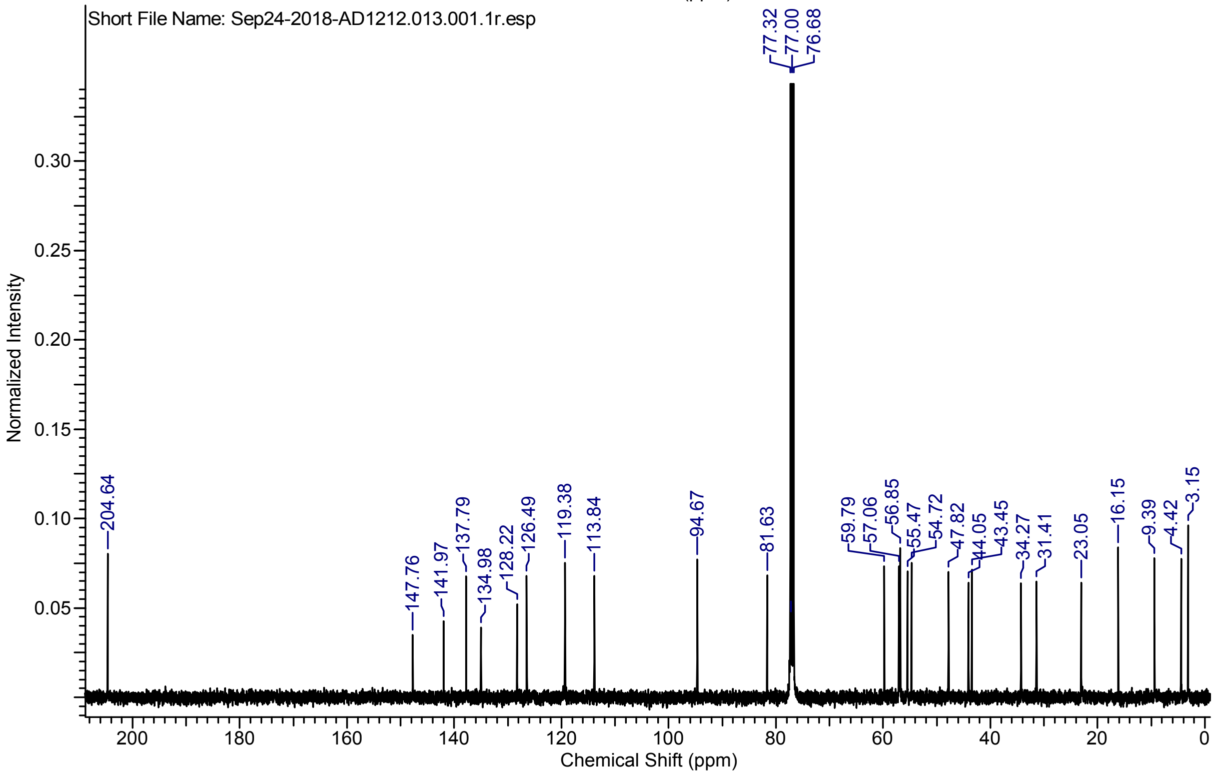

(5α,6R,7R,14α)-(17-Cyclopropylmethyl-4,5-epoxy-7,8-dihydro-3,6-dimethoxy-7β-methyl-6,14-etheno-morphinan-7-yl)-methanol (12)

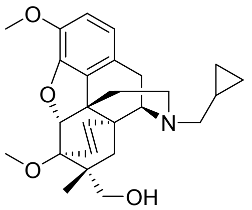

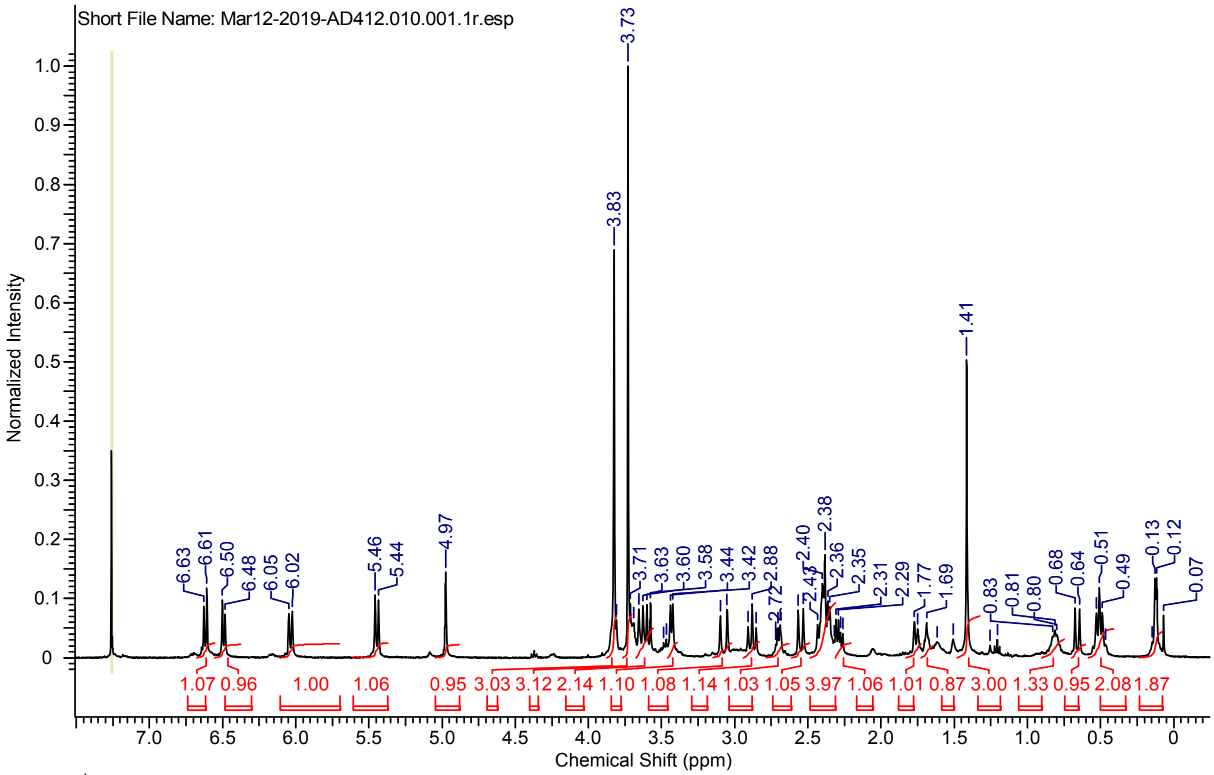

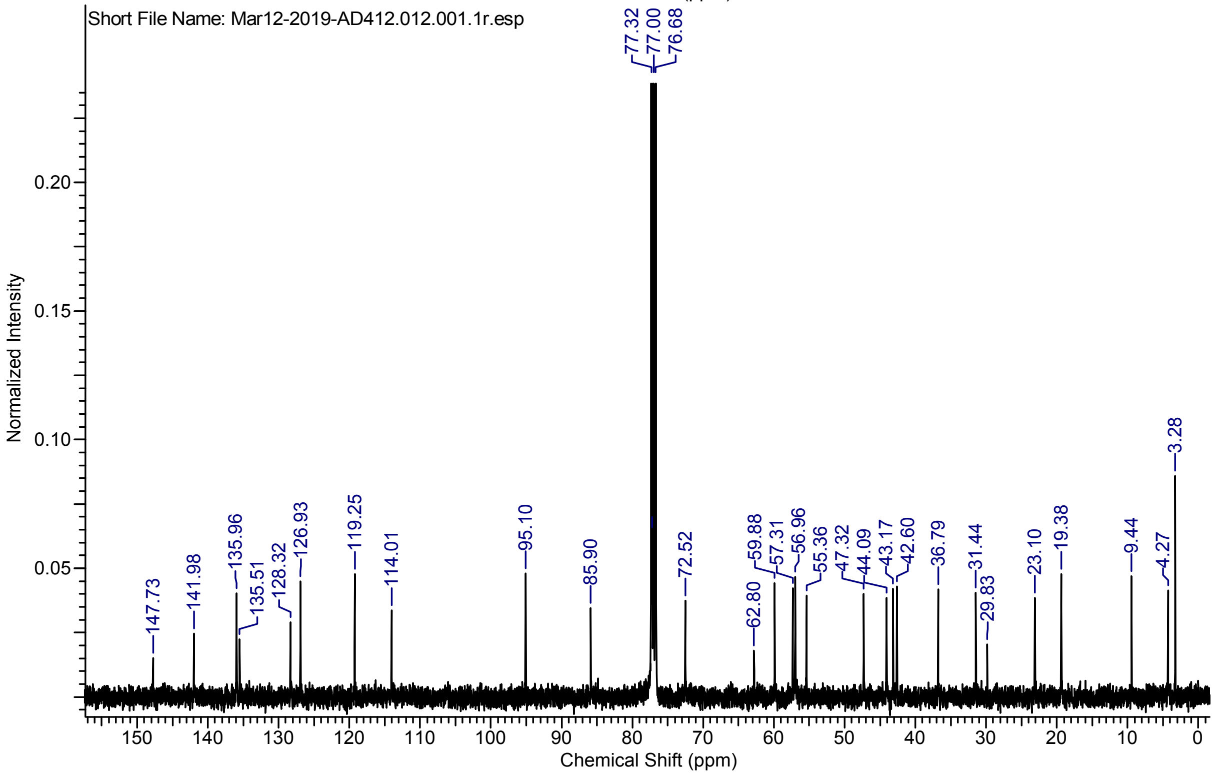

(5α,6R,7R,14α)-(17-Cyclopropylmethyl-4,5-epoxy-7,8-dihydro-3,6-dimethoxy-7β-methyl-6,14-ethano-morphinan-7-yl)-methanol (13)

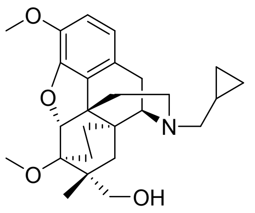

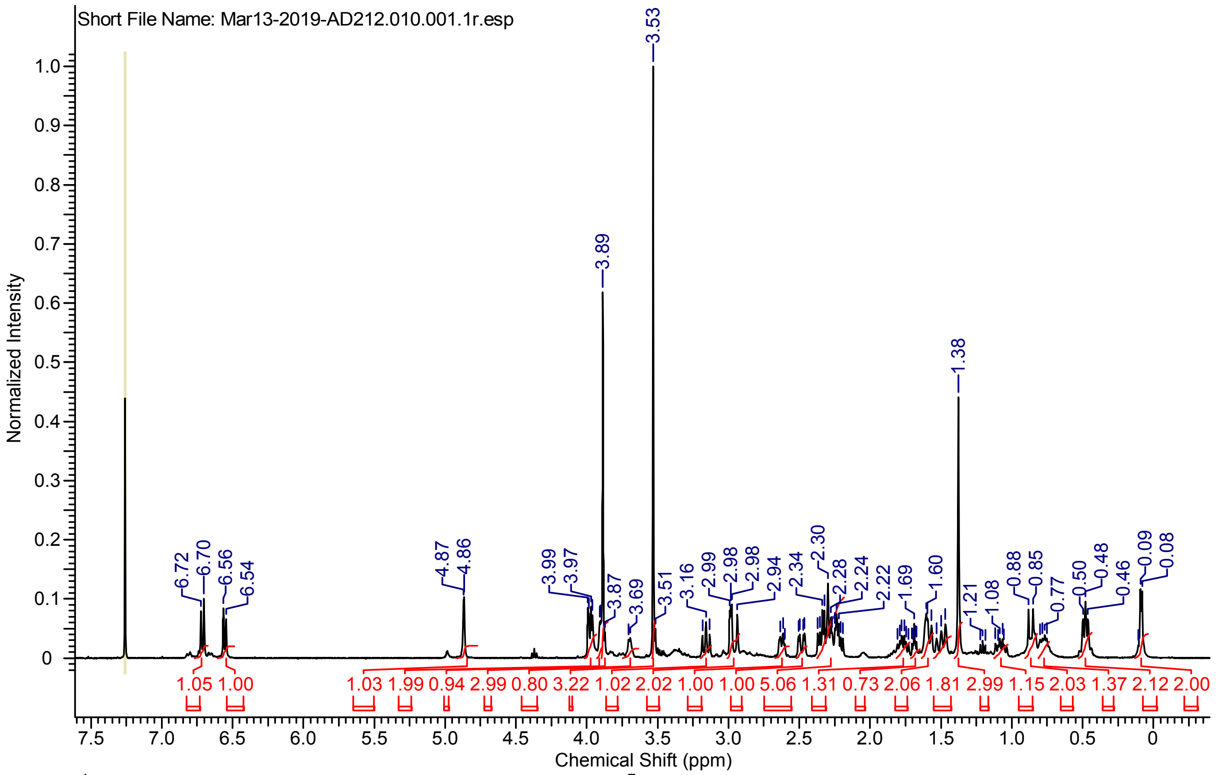

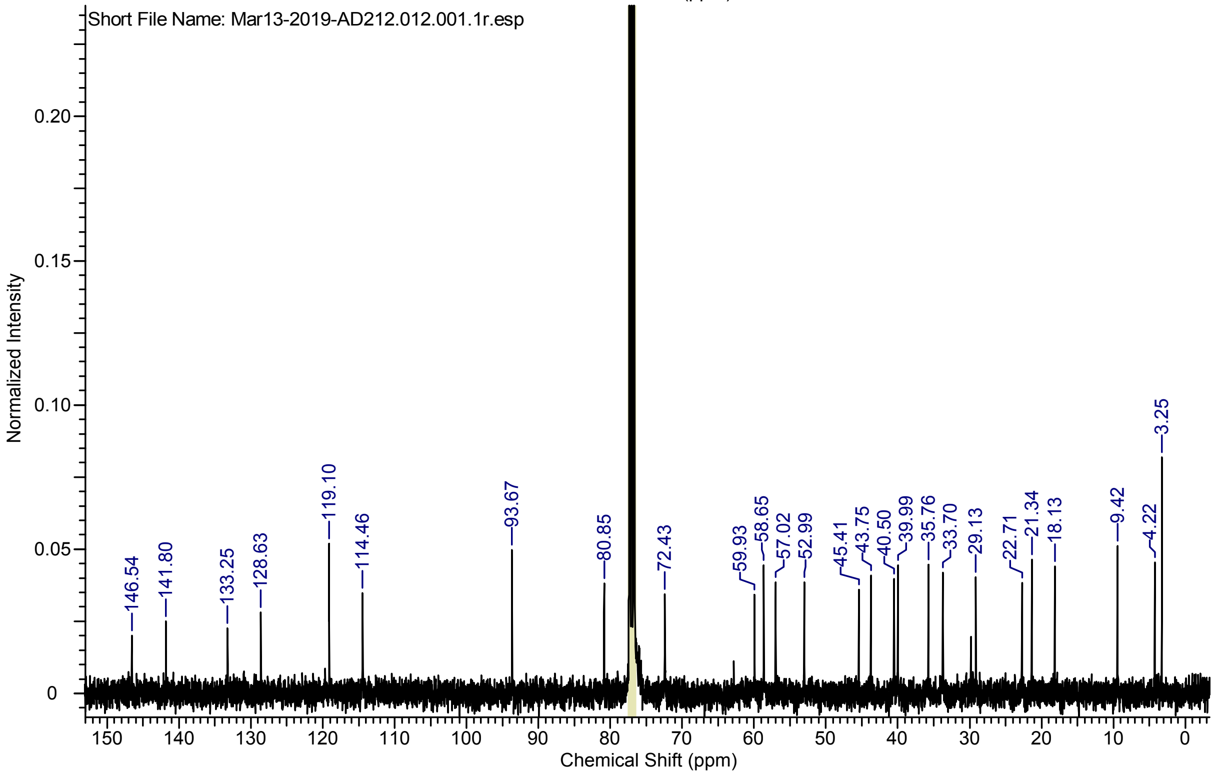

(5α,6R,7R,14α)-(17-cyclopropylmethyl-4,5-epoxy-7,8-dihydro-3-hydroxy-6-methoxy-7β-methyl-6,14-ethano-morphinan-7-yl)-methanol (14)

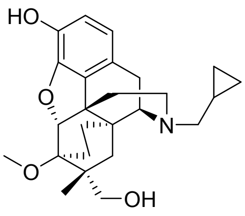

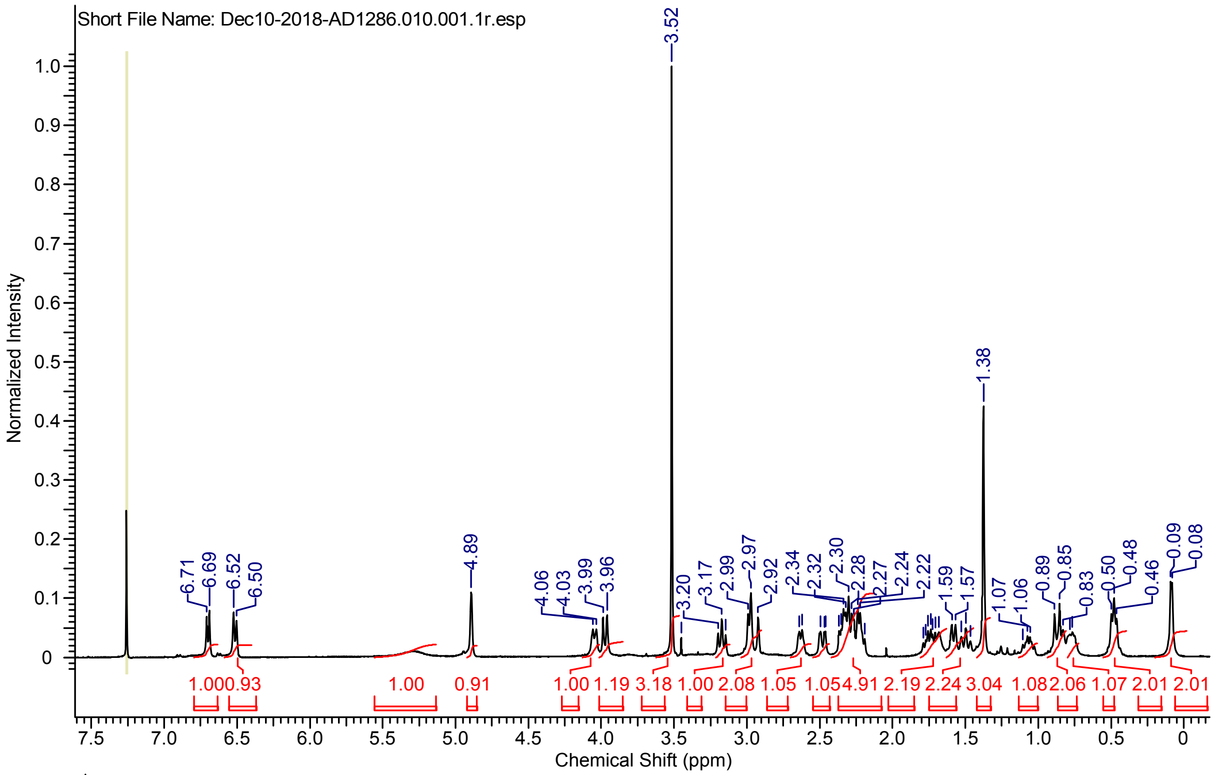

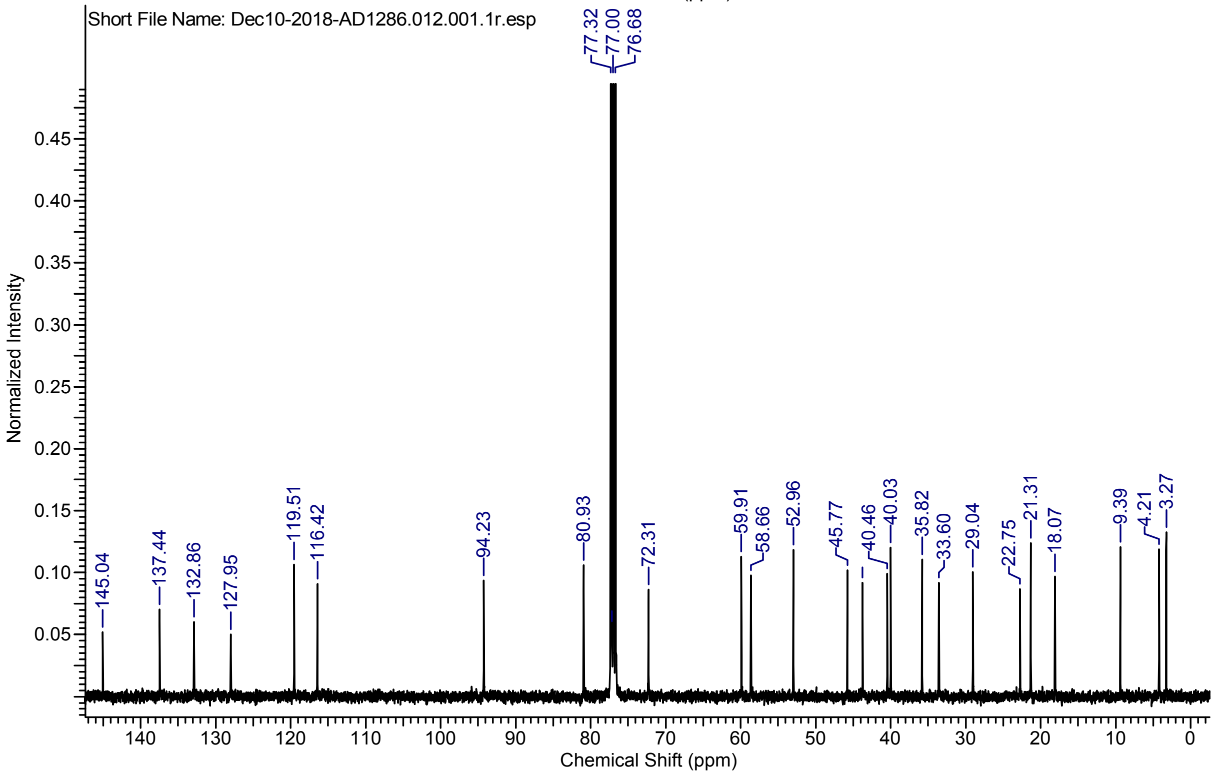

(5α,6R,7R,14α)-1′-(17-cyclopropylmethyl-4,5-epoxy-7,8-dihydro-3-hydroxy-6-methoxy-7β-methyl-6,14-ethano-morphinan-7-yl)-methanol.HCl (14.HCl)

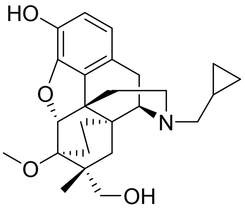

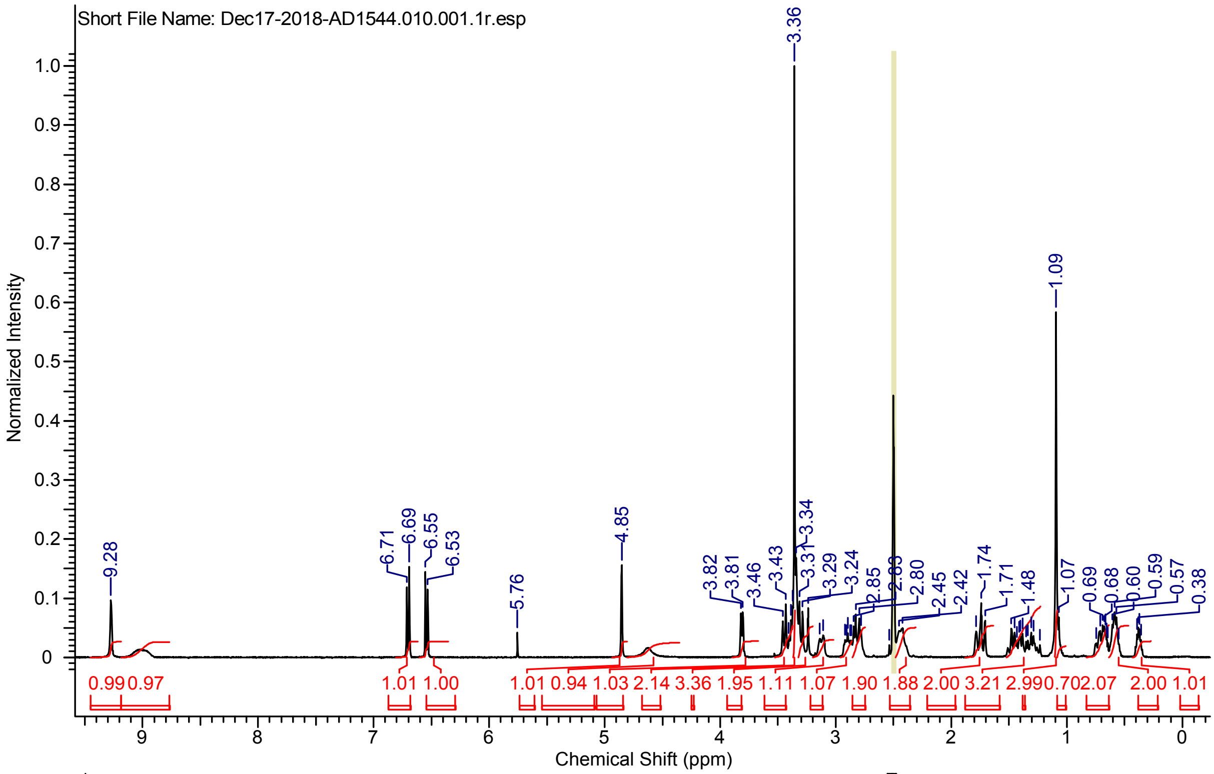

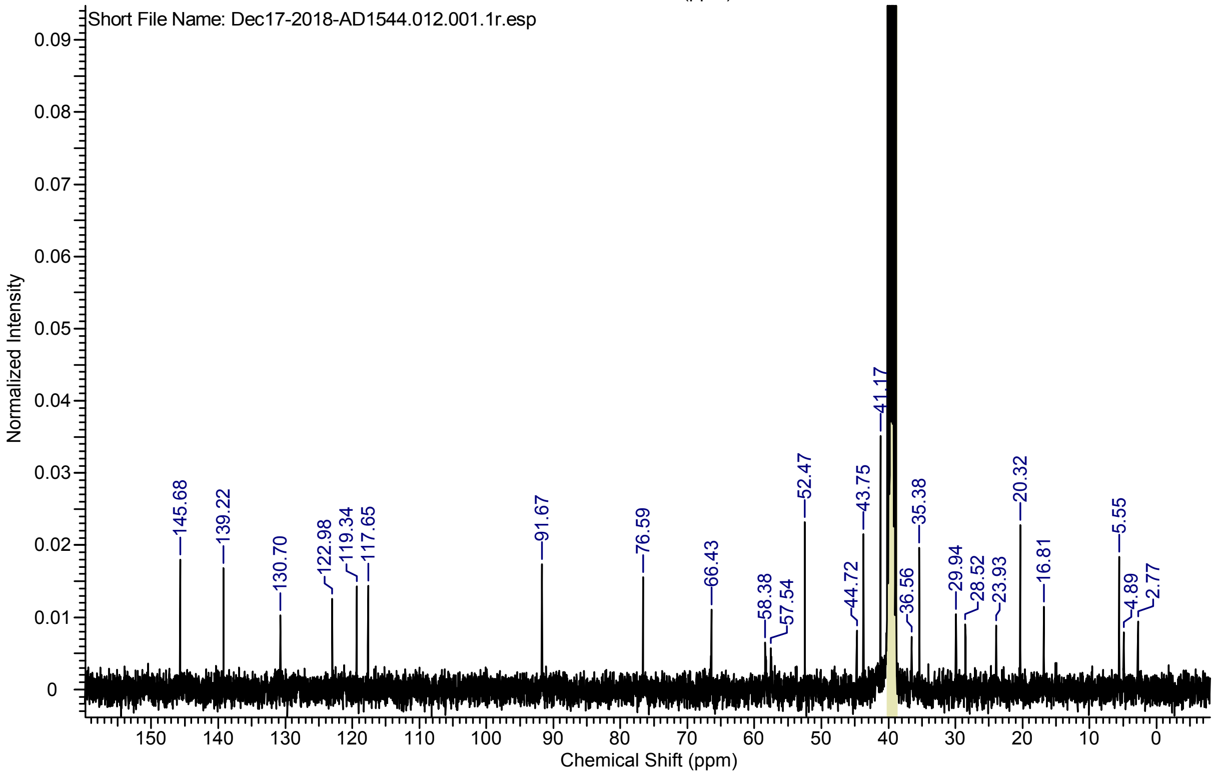

HPLC of 14 at 254nM

Chromatogram Report

**Analysis Info**		Acquisition Date	<illegal time>
Analysis Name	C:\Data\2019-01-04 Alex RP C18\AD345 HCl_RB2_01_4176.d		
Method		Operator	
Sample Name		Instrument	
Comment			

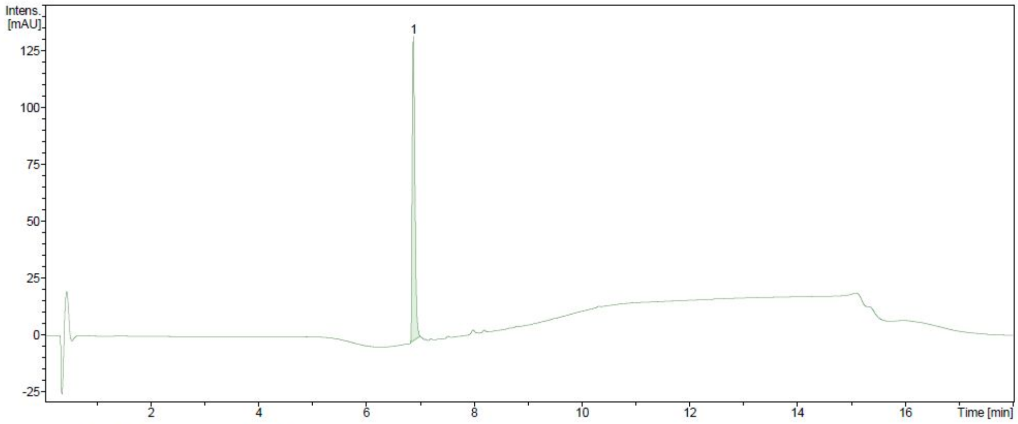

#	RT [min]	Area	Area Frac. %
1	6.9	465.07	100.00

Bruker Compass DataAnalysis 4.3 printed: 07-Jan-19 11:16:51 AM by: bdal 1 of 1

HPLC of 14 at 280nM

Chromatogram Report

**Analysis Info**		Acquisition Date	<illegal time>
Analysis Name	C:\Data\2019-01-04 Alex RP C18\AD345 HCl_RB2_01_4176.d		
Method		Operator	
Sample Name		Instrument	
Comment			

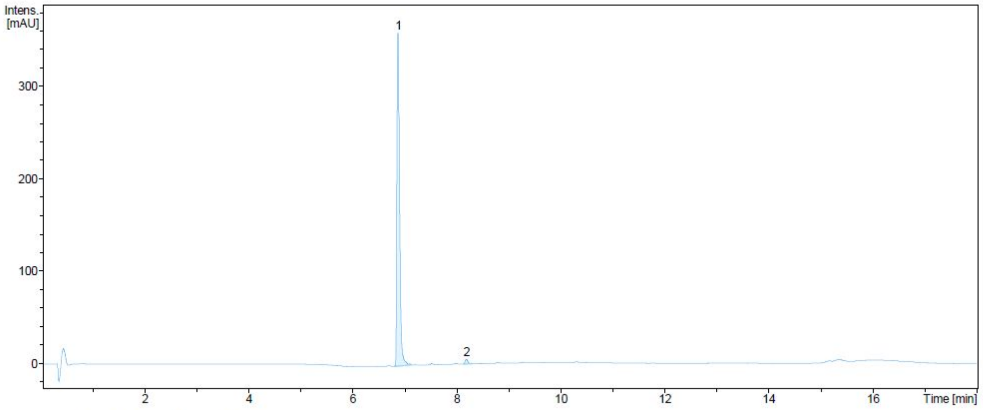

#	RT [min]	Area	Area Frac. %
1	6.9	1291.985	98.73
2	8.2	16.623	1.27

Bruker Compass DataAnalysis 4.3 printed: 07-Jan-19 11:20:15 AM by: bdal 1 of 1

(5α,6R,7R,14α)-1′-(17-Cyclopropylmethyl-4,5-epoxy-7,8-dihydro-3,6-dimethoxy-7β-methyl-6,14-etheno-morphinan-7-yl)-ethan-1′-ol (15)

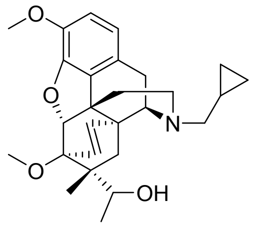

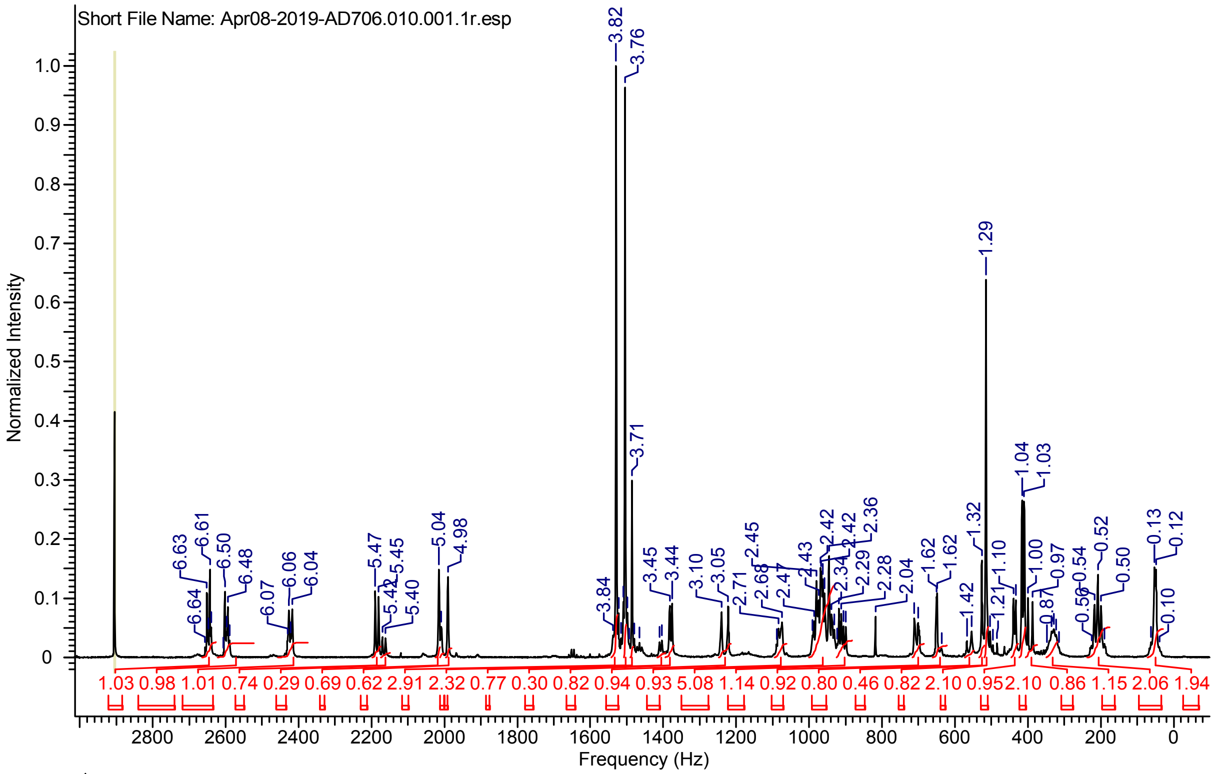

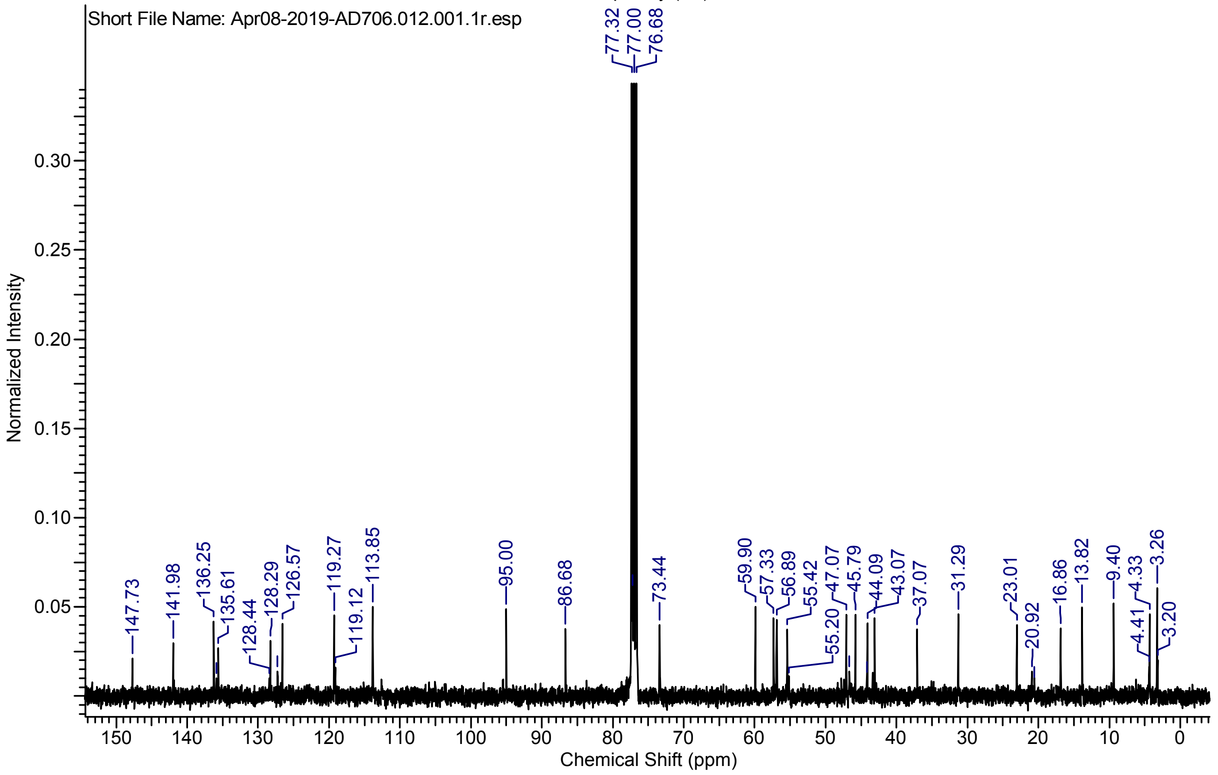

(5α,6R,7R,14α)-1′-(17-Cyclopropylmethyl-4,5-epoxy-7,8-dihydro-3,6-dimethoxy-7β-methyl-6,14-ethano-morphinan-7-yl)-ethan-1′-ol (16)

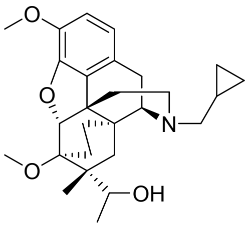

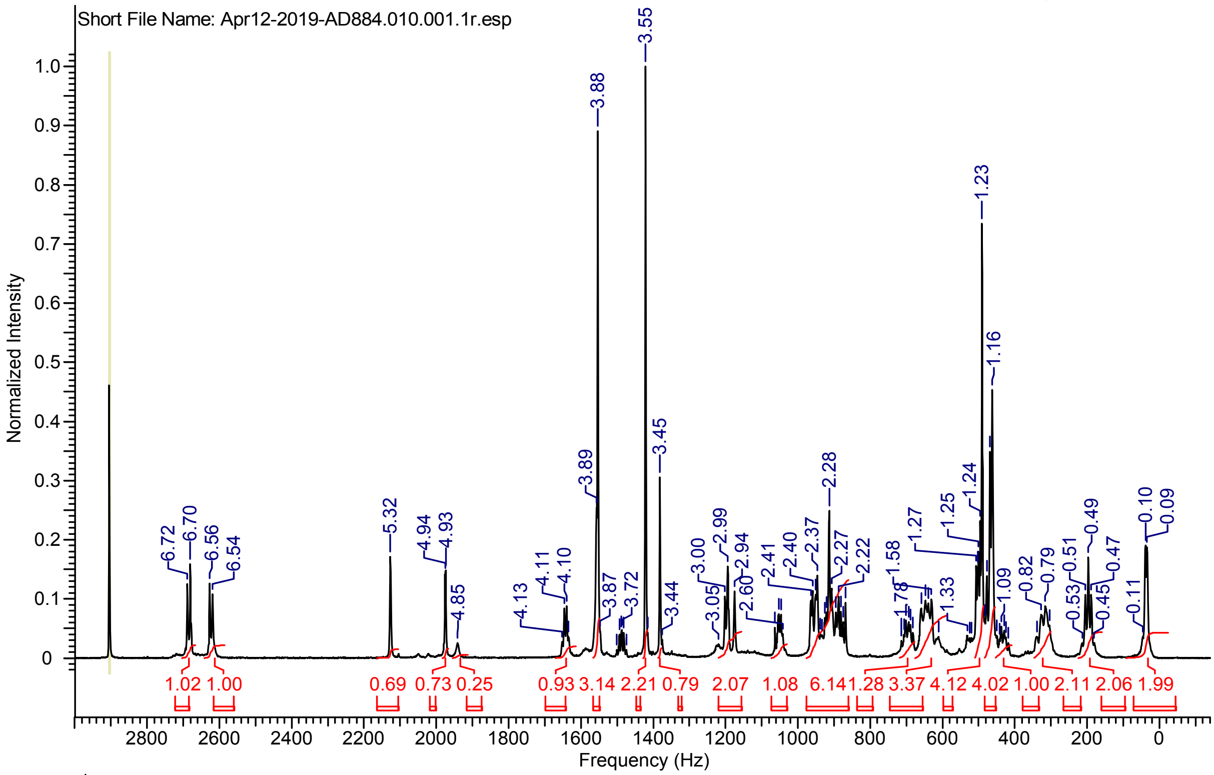

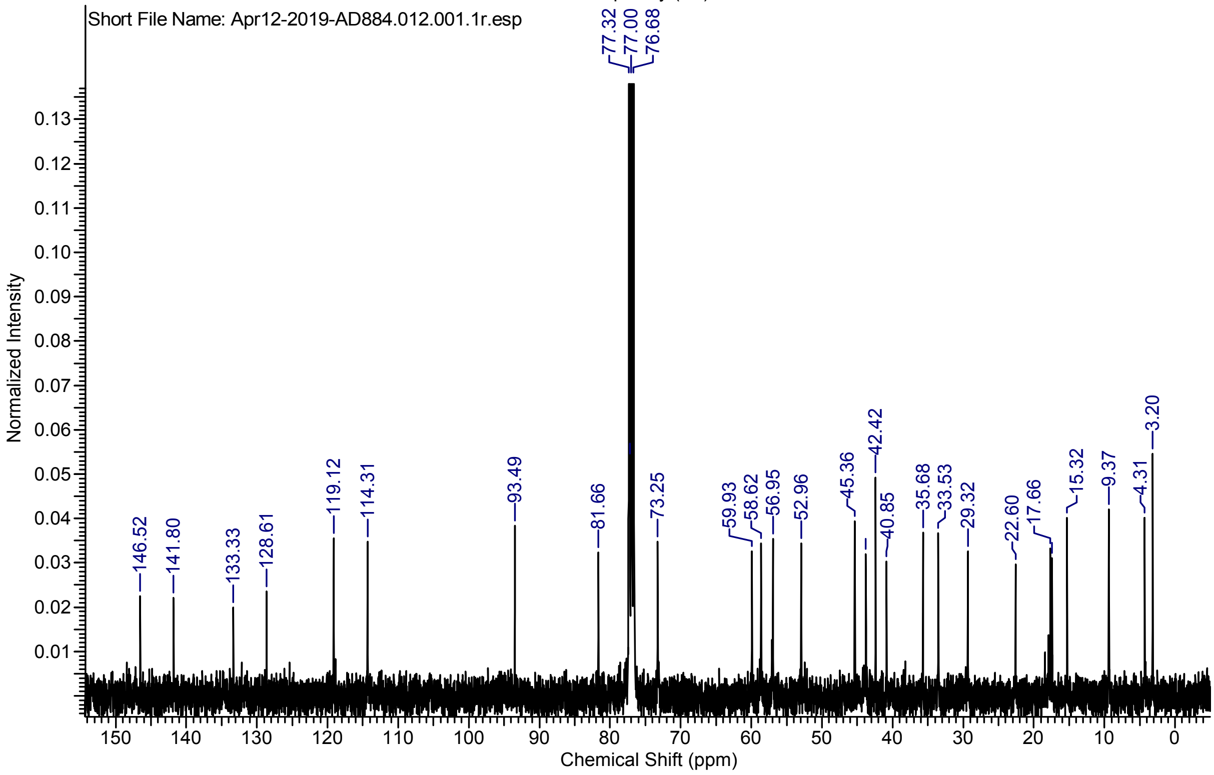

(1'R,5α,6R,7R,14α)-1′-(17-Cyclopropylmethyl-4,5-epoxy-7,8-dihydro-3,6-dimethoxy-7β-methyl-6,14-ethano-morphinan-7-yl)-ethan-1′-ol (16a)

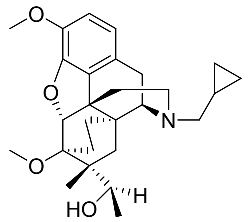

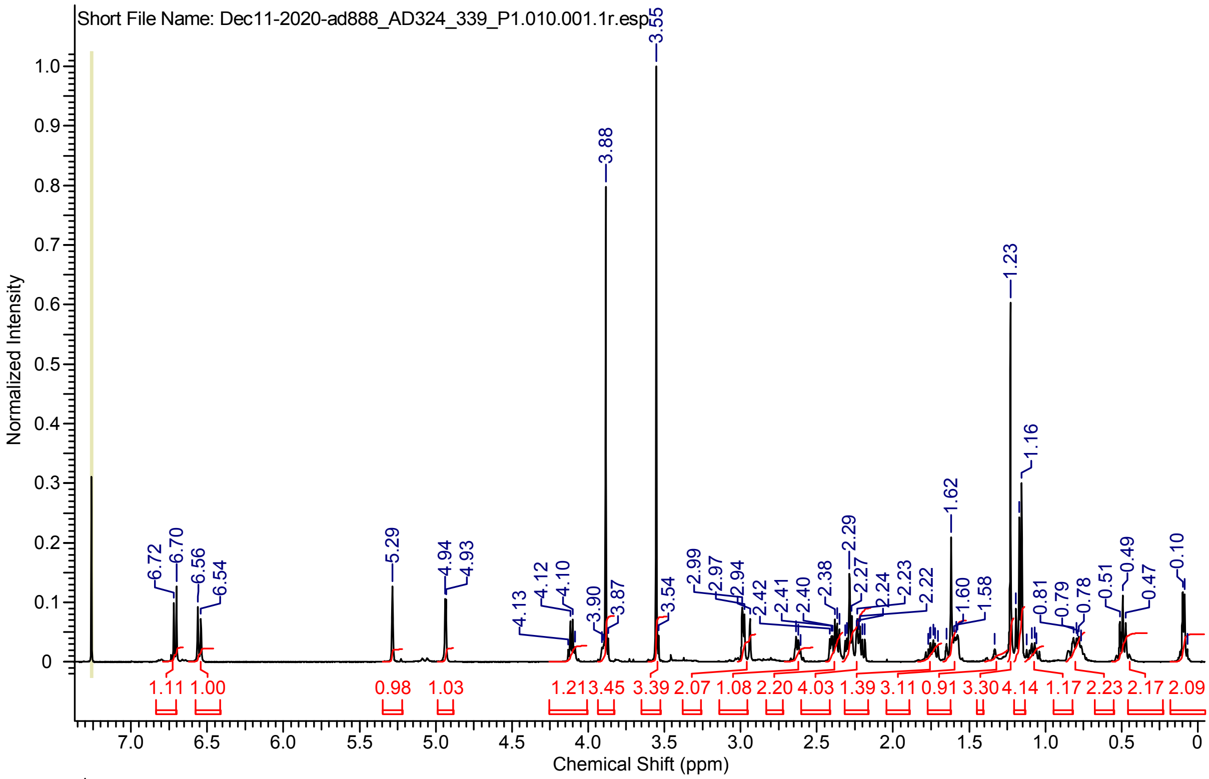

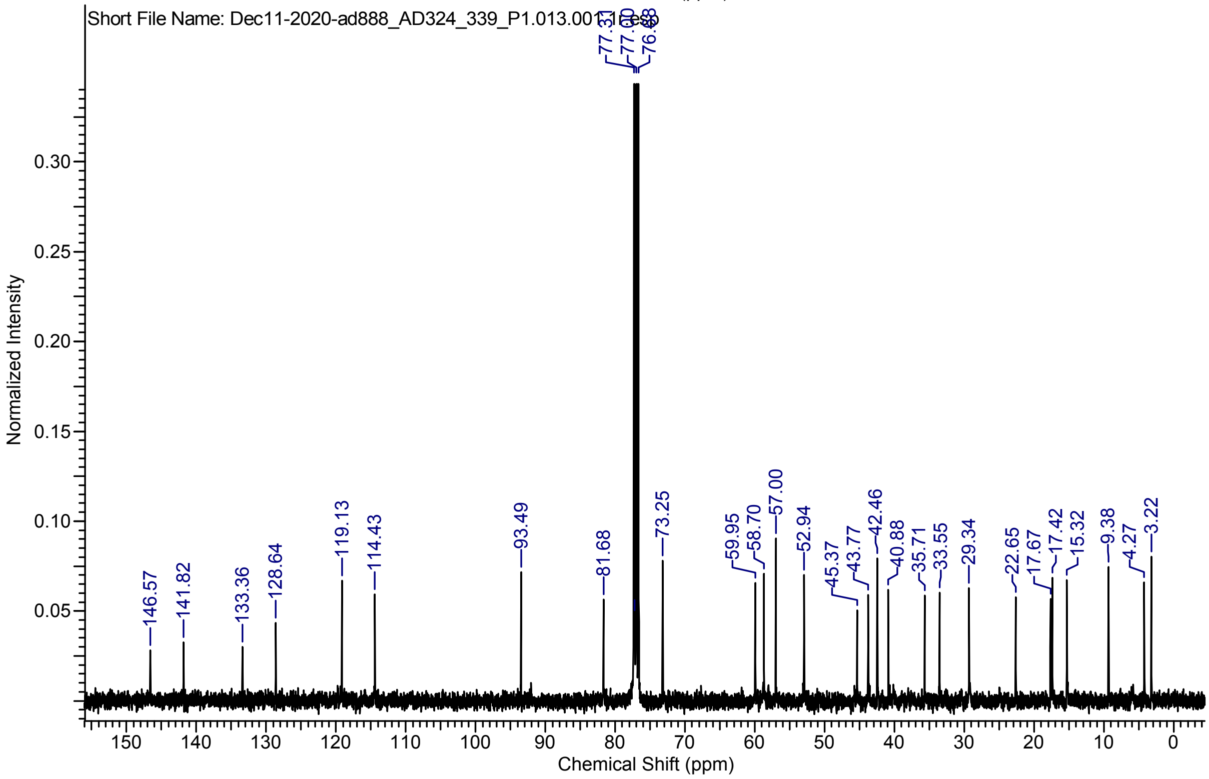

Crystal structure of 16a

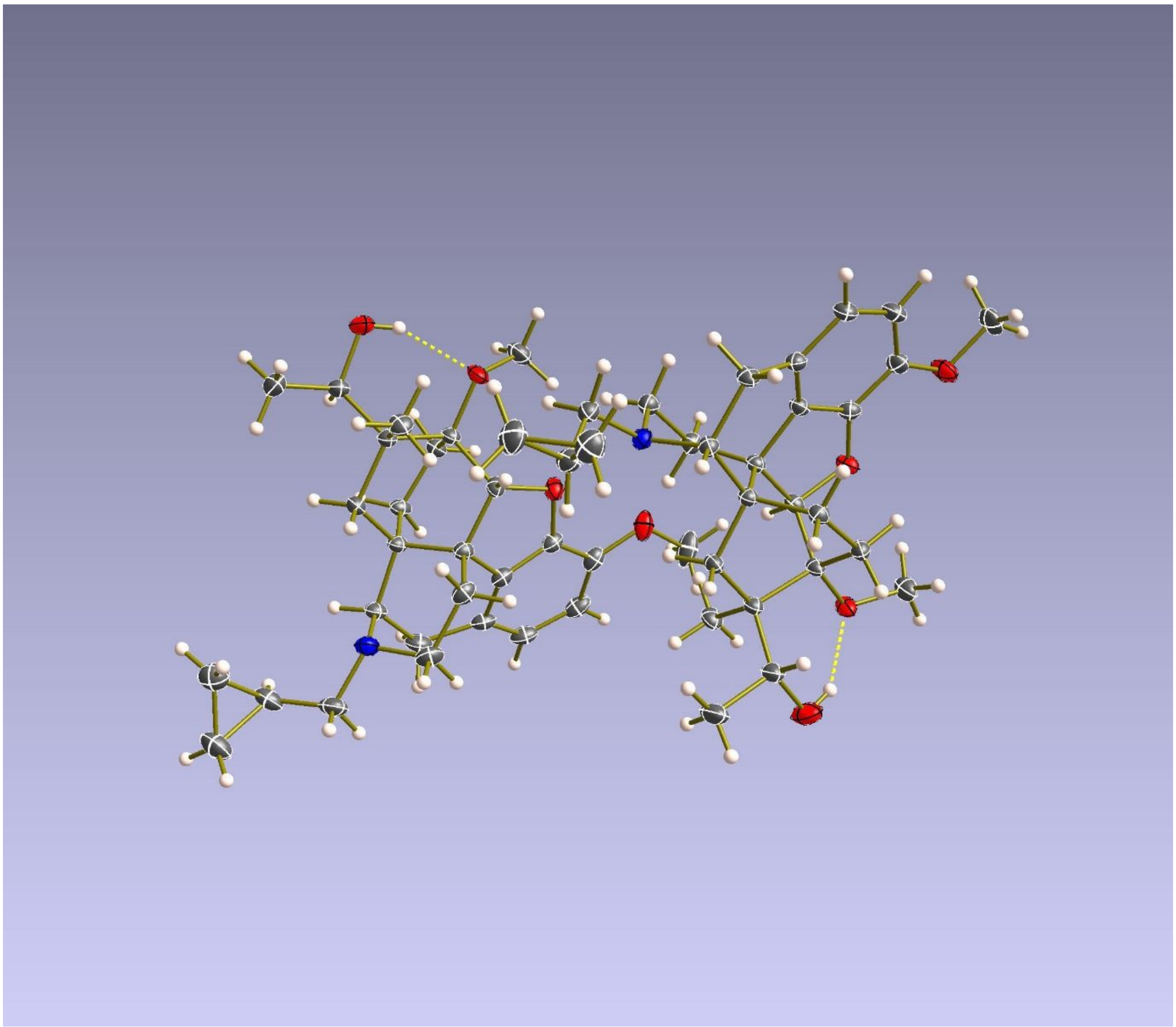

(1’S,5α,6R,7R,14α)-1′-(17-Cyclopropylmethyl-4,5-epoxy-7,8-dihydro-3,6-dimethoxy-7β-methyl-6,14-ethano-morphinan-7-yl)-ethan-1′-ol (16b)

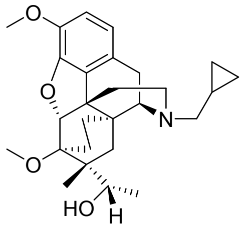

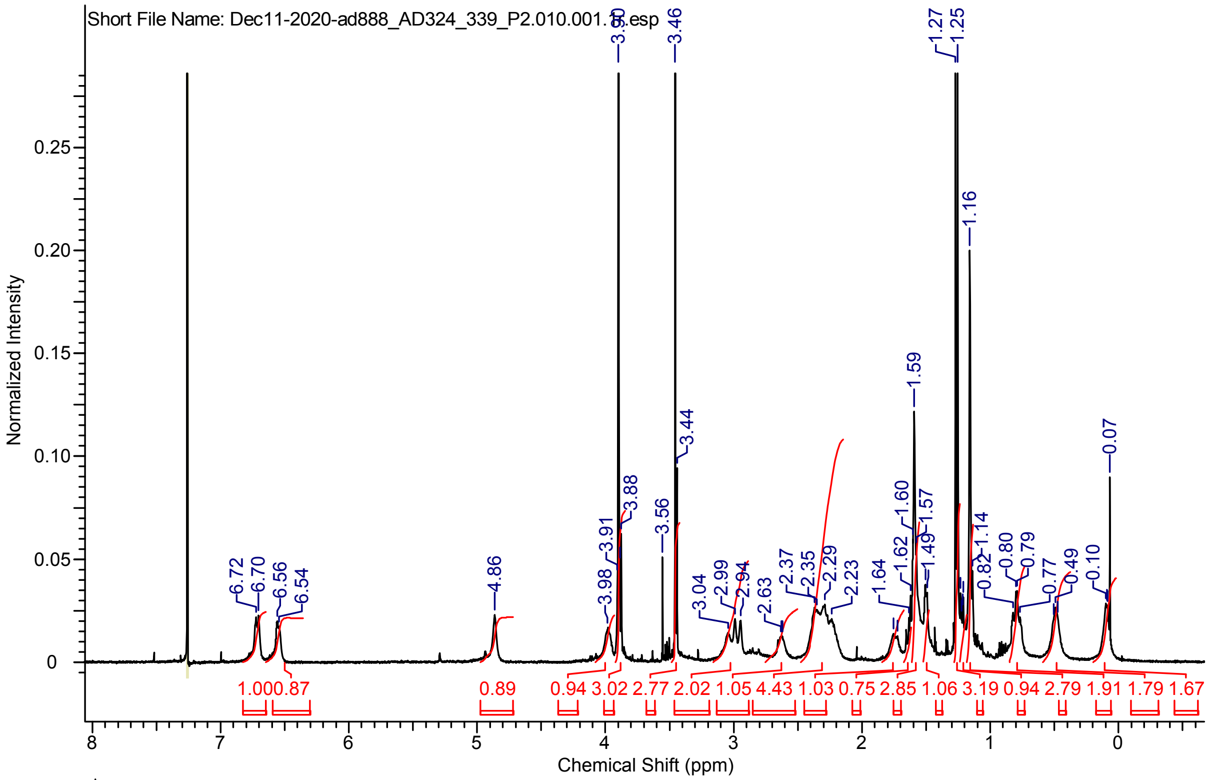

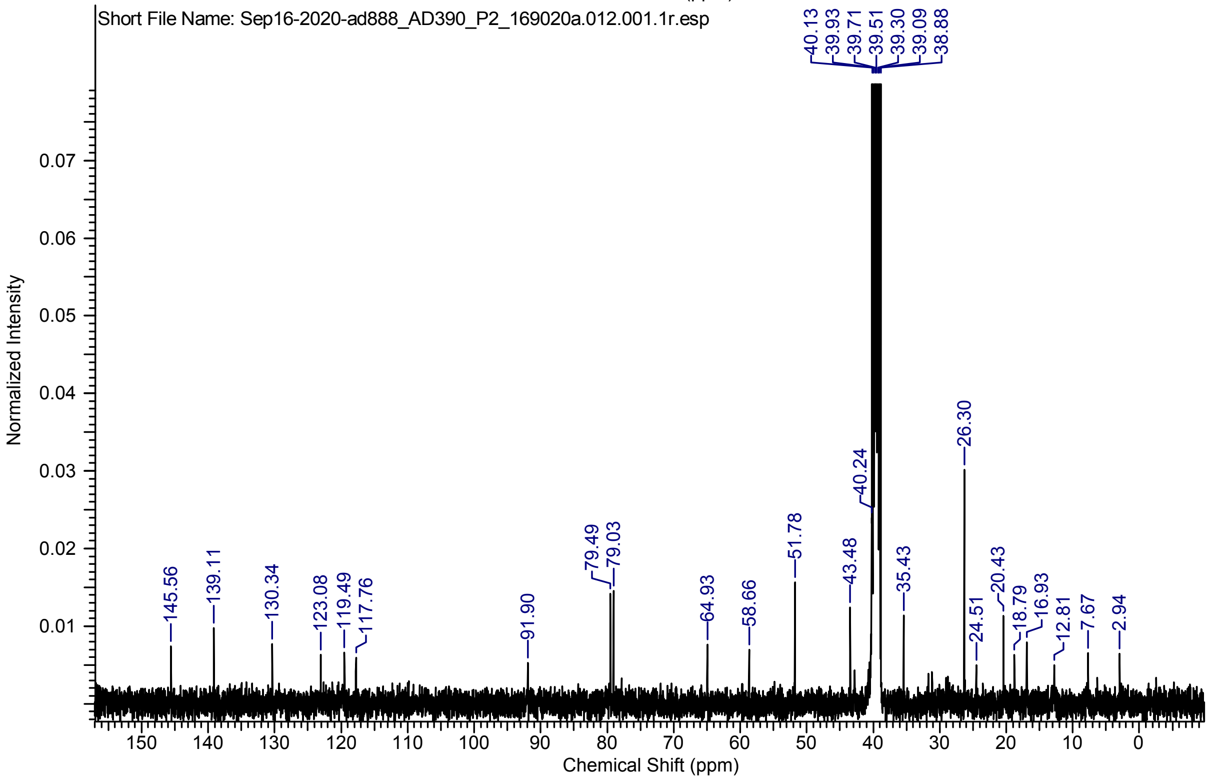

(1’R,5α,6R,7R,14α)-1′-(17-Cyclopropylmethyl-4,5-epoxy-7,8-dihydro-3-hydroxy-6-methoxy-7β-methyl-6,14-ethano-morphinan-7-yl)-ethan-1′-ol (17a)

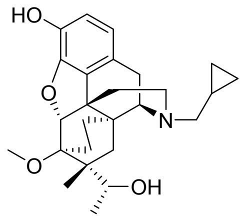

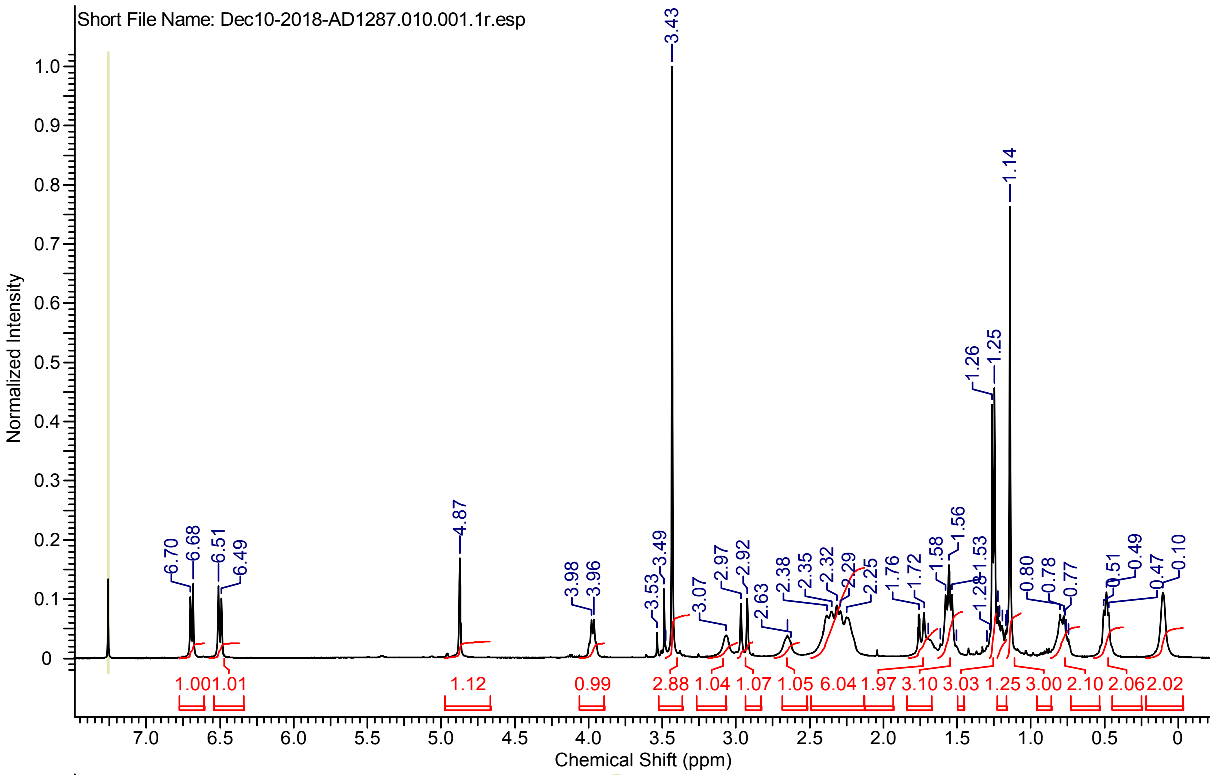

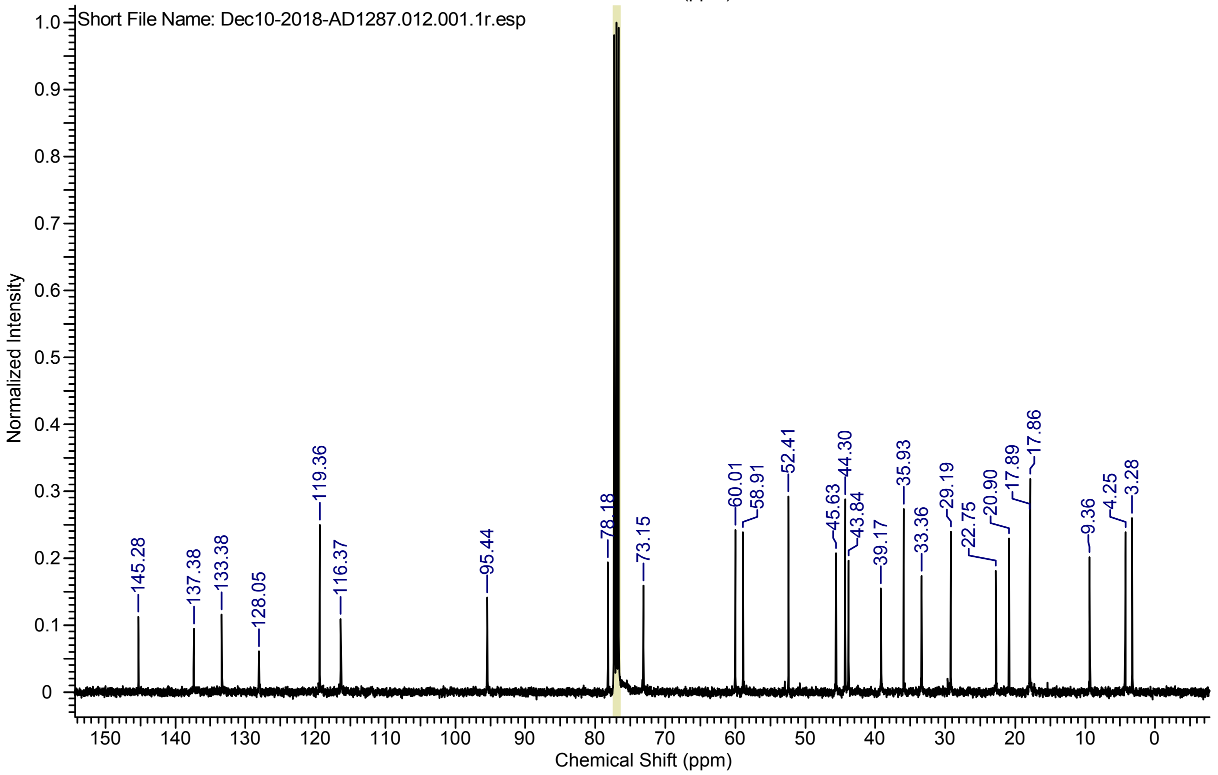

(1'R,5α,6R,7R,14α)-1′-(17-Cyclopropylmethyl-4,5-epoxy-7,8-dihydro-3-hydroxy-6-methoxy-7β-methyl-6,14-ethano-morphinan-7-yl)-ethan-1′-ol.HCl (17a.HCl)

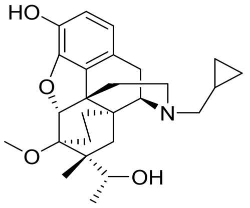

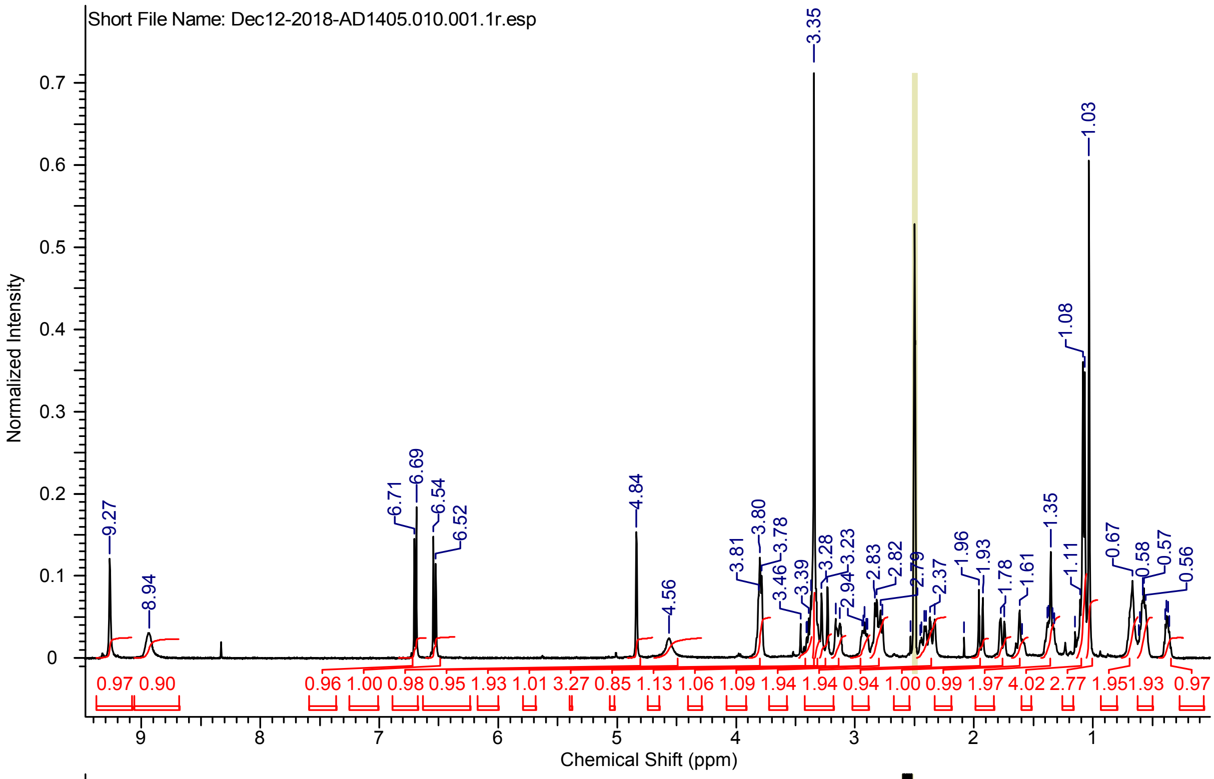

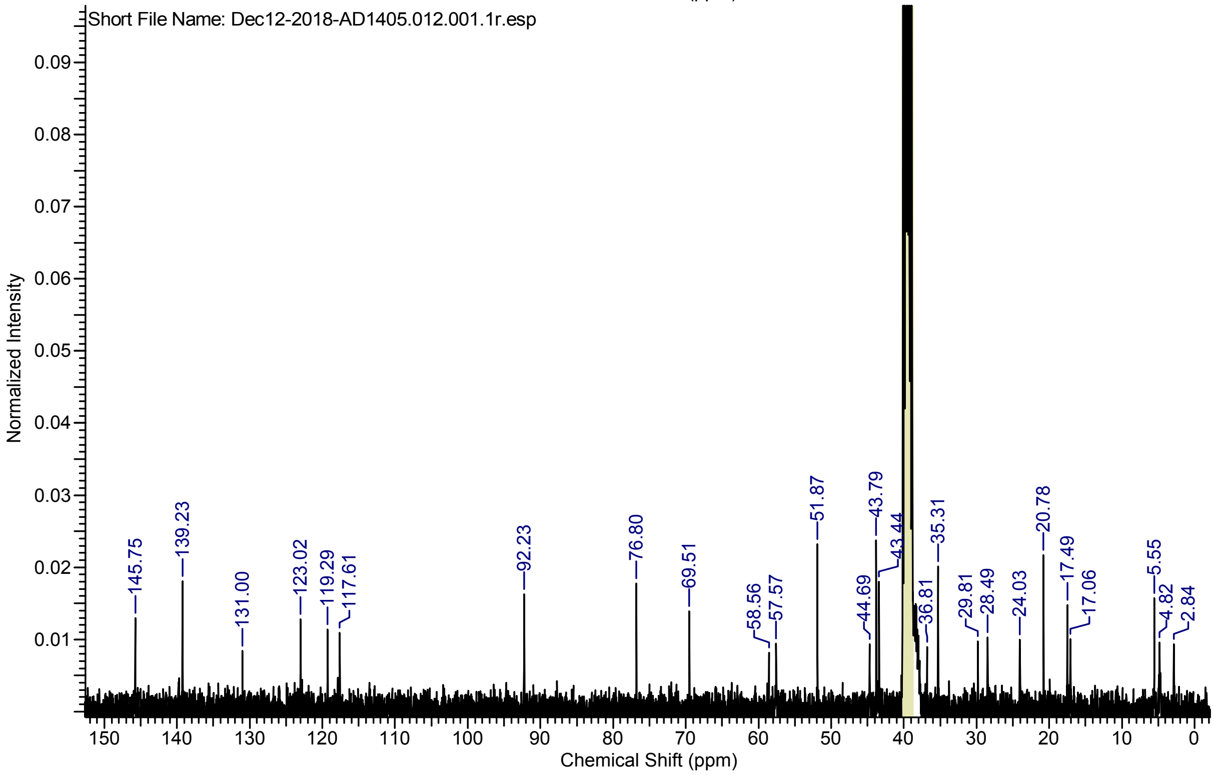

HPLC of 17a at 254 nm

Chromatogram Report

Analysis Info

Analysis Name	C:\Data\2019-01-04 Alex RP C18\AD346 HCl_RB3_01_4178.d	Acquisition Date	<illegal time>
Method		Operator	
Sample Name		Instrument	
Comment			

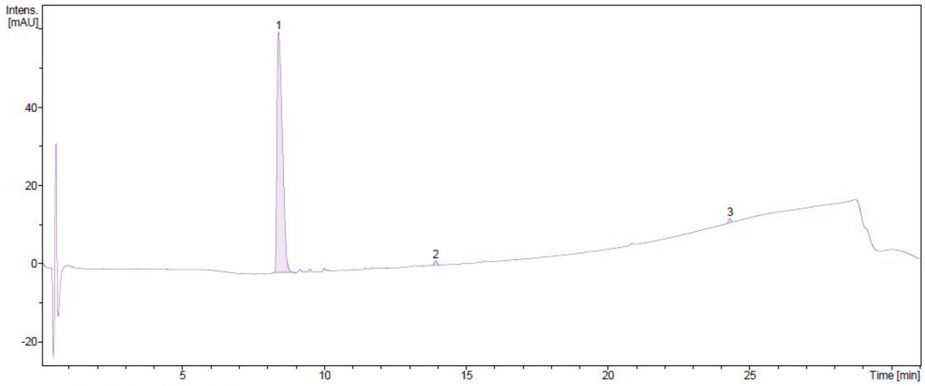

#	RT [min]	Area	Area Frac. %
1	8.4	814.1971	98.30
2	13.9	7.7840	0.94
3	24.3	6.2666	0.76

Bruker Compass DataAnalysis 4.3 printed: 07-Jan-19 11:33:08 AM by: bdal 1 of 1

HPLC of 17a at 280 nm

Chromatogram Report

Analysis Info

Analysis Name	C:\Data\2019-01-04 Alex RP C18\AD346 HCl_RB3_01_4178.d	Acquisition Date	<illegal time>
Method		Operator	
Sample Name		Instrument	
Comment			

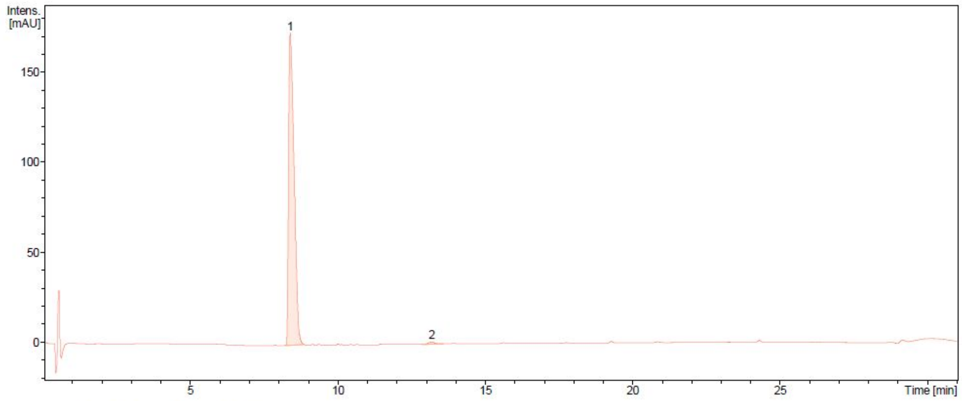

#	RT [min]	Area	Area Frac. %
1	8.4	2258.163	99.24
2	13.2	17.209	0.76

Bruker Compass DataAnalysis 4.3 printed: 07-Jan-19 11:35:47 AM by: bdal 1 of 1

(1'S,5α,6R,7R,14α)-1′-(17-Cyclopropylmethyl-4,5-epoxy-7,8-dihydro-3-hydroxy-6-methoxy-7β-methyl-6,14-ethano-morphinan-7-yl)-ethan-1′-ol (17b)

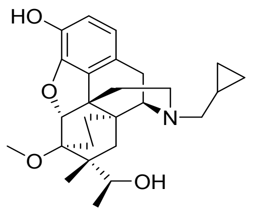

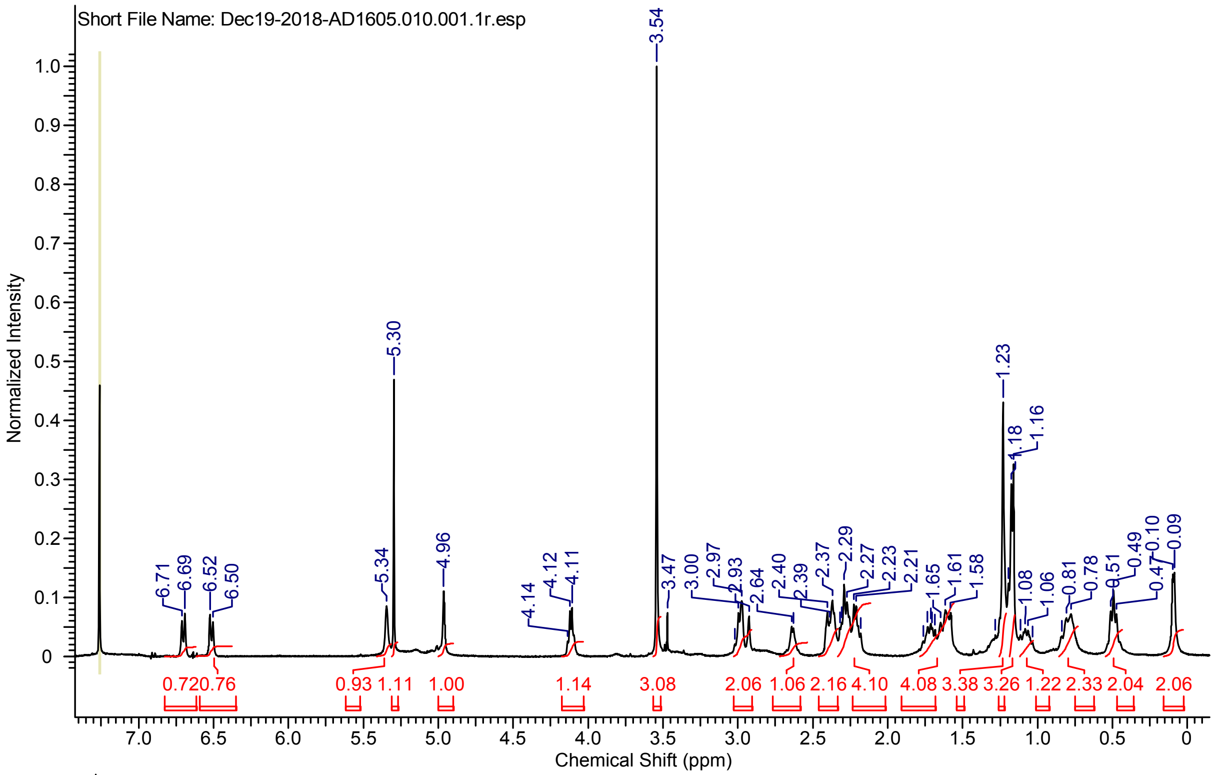

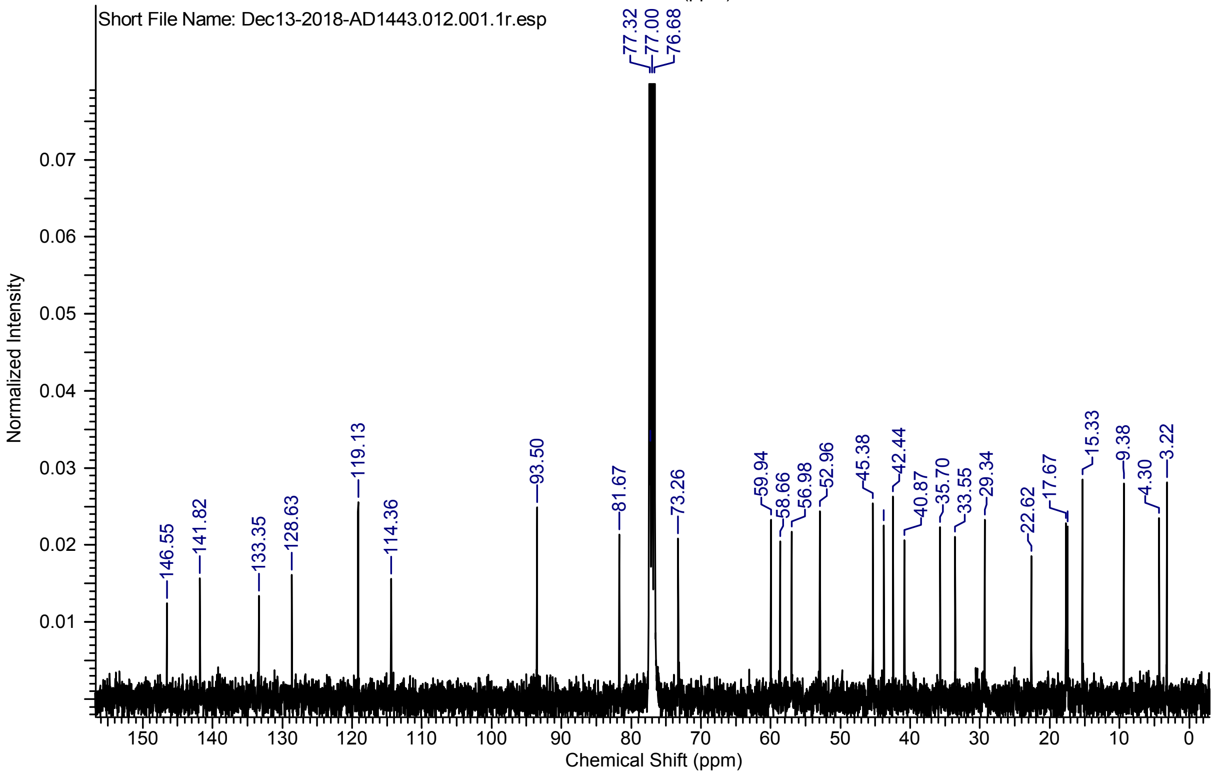

(1’R,5α,6R,7R,14α)-1′-(17-Cyclopropylmethyl-4,5-epoxy-7,8-dihydro-3-hydroxy-6-methoxy-7β-methyl-6,14-ethano-morphinan-7-yl)-ethan-1′-ol.HCl (17b.HCl)

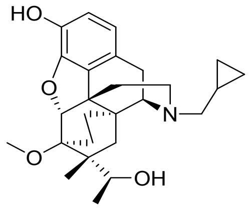

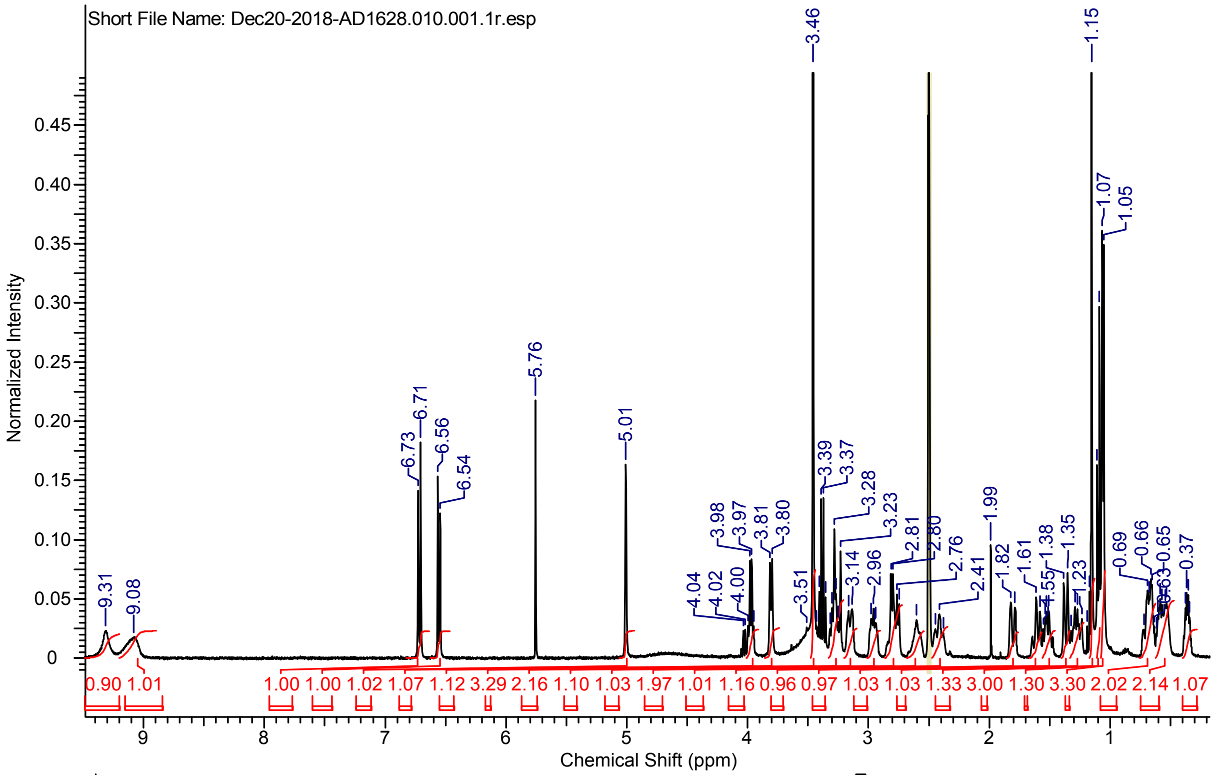

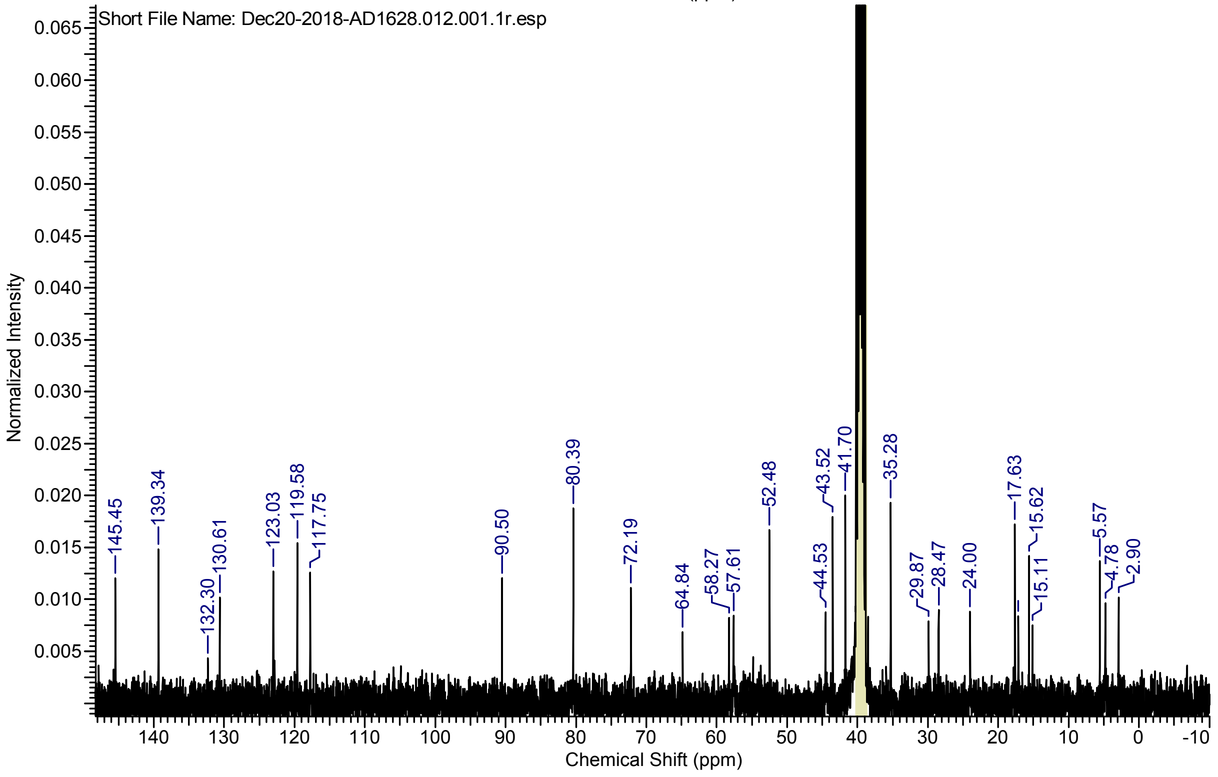

HPLC of 17b at 254 nm

Chromatogram Report

Analysis Info

Analysis Name C:\Data\2019-01-04 Alex RP C18\AD344 HCl_RB5_01_4180.d

Method

Sample Name

Comment

Acquisition Date <illegal time>

Operator

Instrument

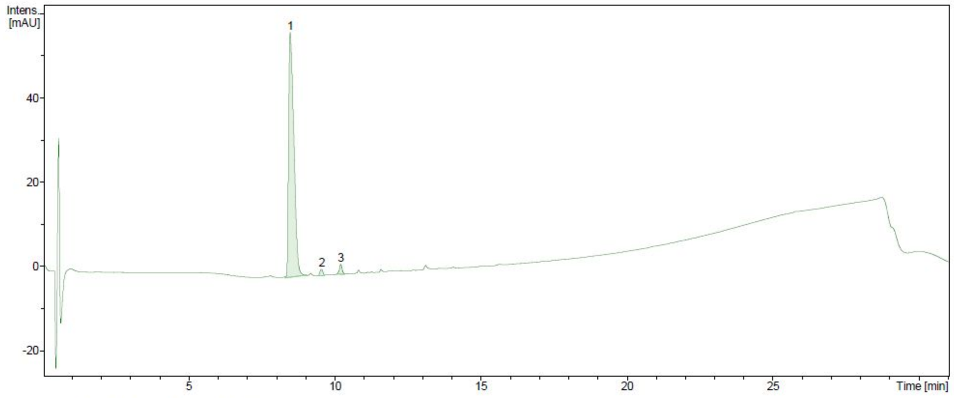

#	RT [min]	Area	Area Frac. %
1	8.5	720.7558	96.70
2	9.5	9.7405	1.31
3	10.2	14.8669	1.99

Bruker Compass DataAnalysis 4.3 printed: 07-Jan-19 12:16:47 PM by: bdal 1 of 1

HPLC of 17b at 280 nm

Chromatogram Report

Analysis Info

Analysis Name C:\Data\2019-01-04 Alex RP C18\AD344 HCl_RB5_01_4180.d

Method

Sample Name

Comment

Acquisition Date <illegal time>

Operator

Instrument

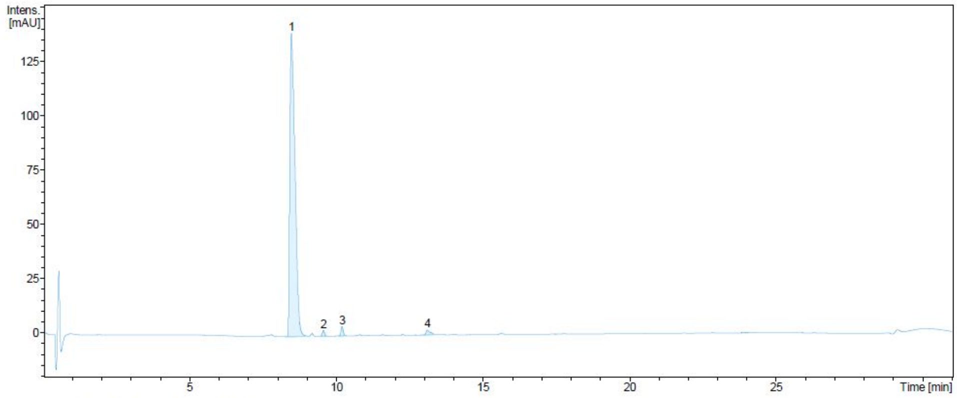

#	RT [min]	Area	Area Frac. %
1	8.5	1704.409	96.90
2	9.6	13.066	0.74
3	10.2	21.302	1.21
4	13.1	20.105	1.14

Bruker Compass DataAnalysis 4.3 printed: 07-Jan-19 12:18:45 PM by: bdal 1 of 1

(5α,6R,7R,14α)-1′-(1-Cyclopropylmethyl-4,5-epoxy-7,8-dihydro-3,6-dimethoxy-7β-methyl-6,14-ethano-morphinan-7-yl)-ethanone (18)

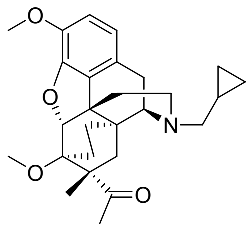

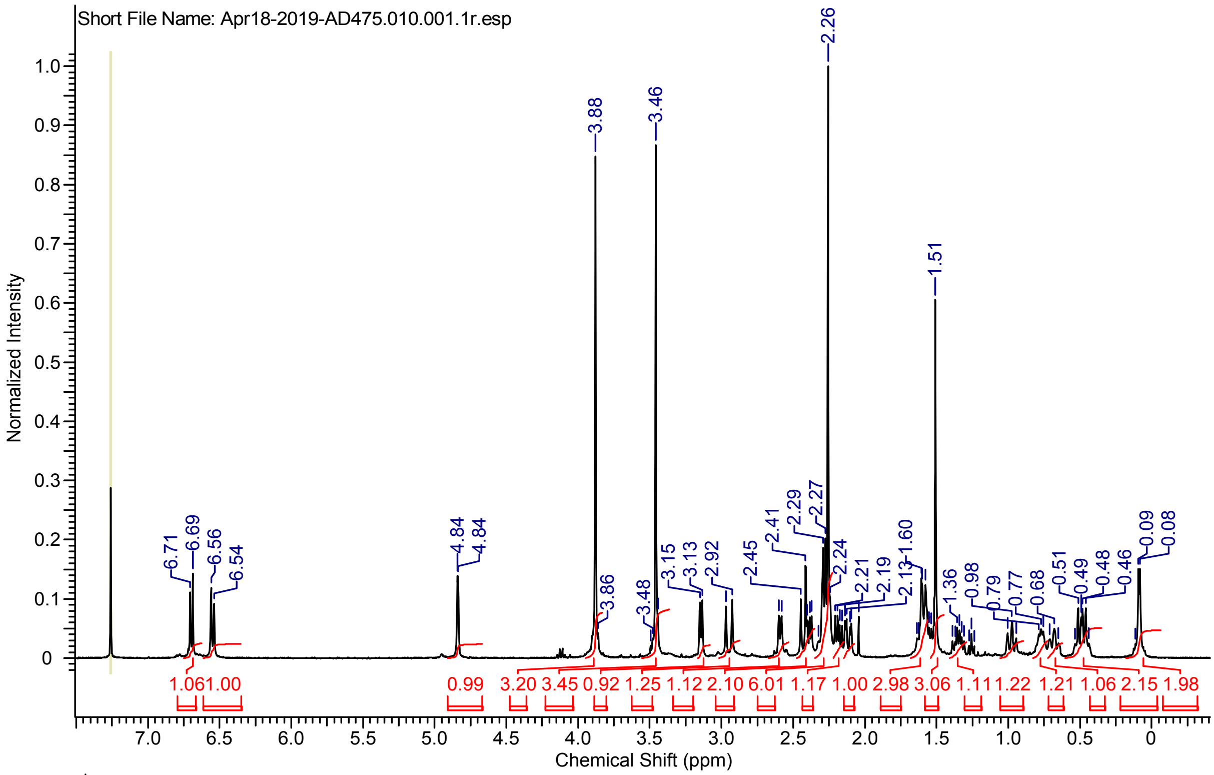

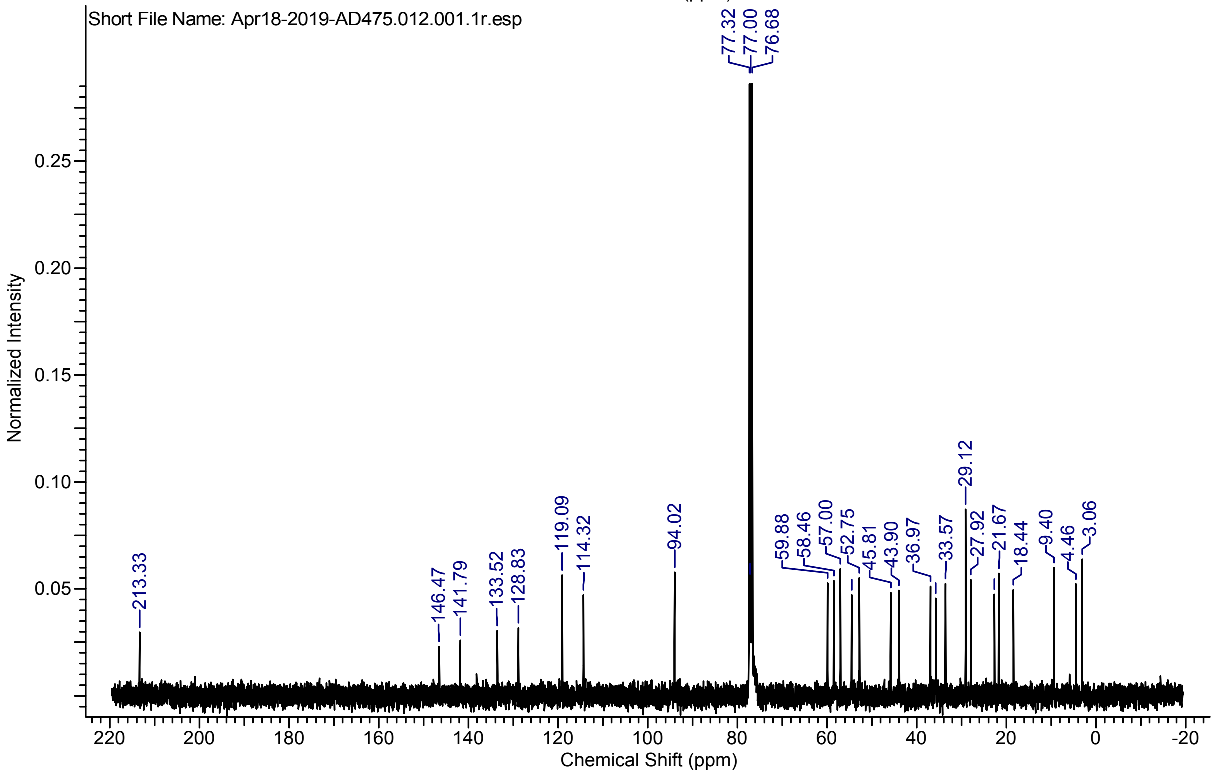

(5α,6R,7R,14α)-2′-(17-Cyclopropylmethyl-4,5-epoxy-7,8-dihydro-3,6-dimethoxy-7β-methyl-6,14-ethano-morphinan-7-yl)-propan-2′-ol (19)

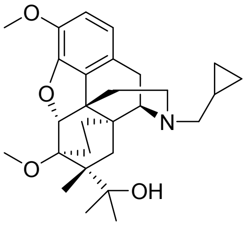

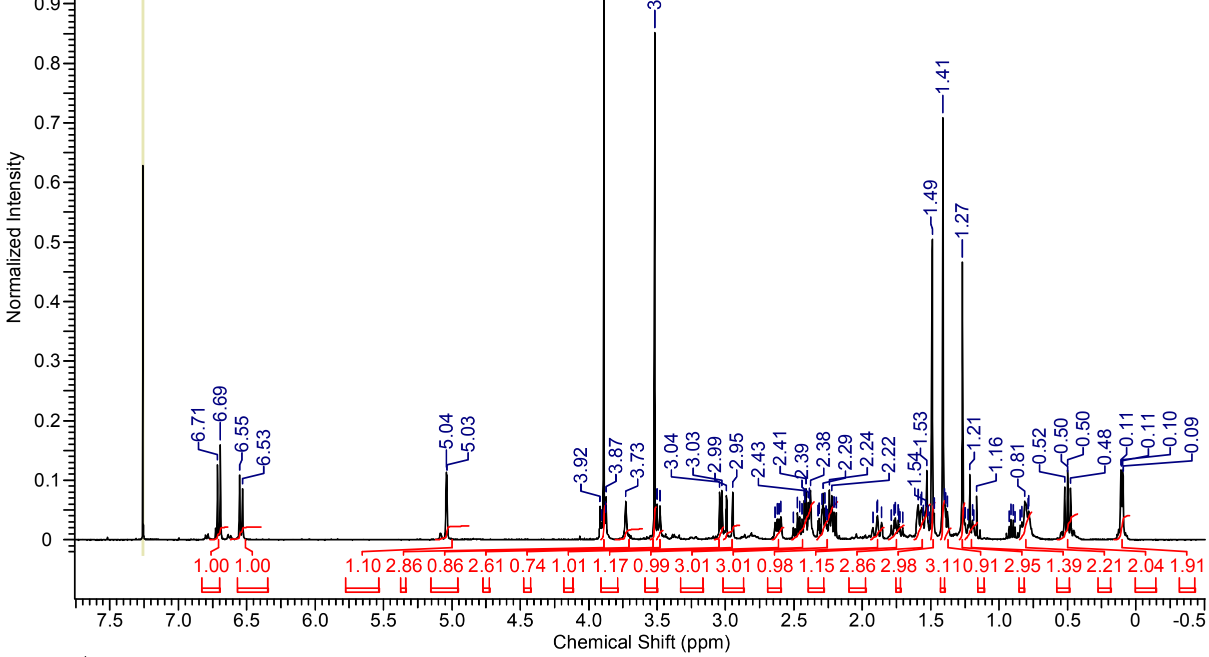

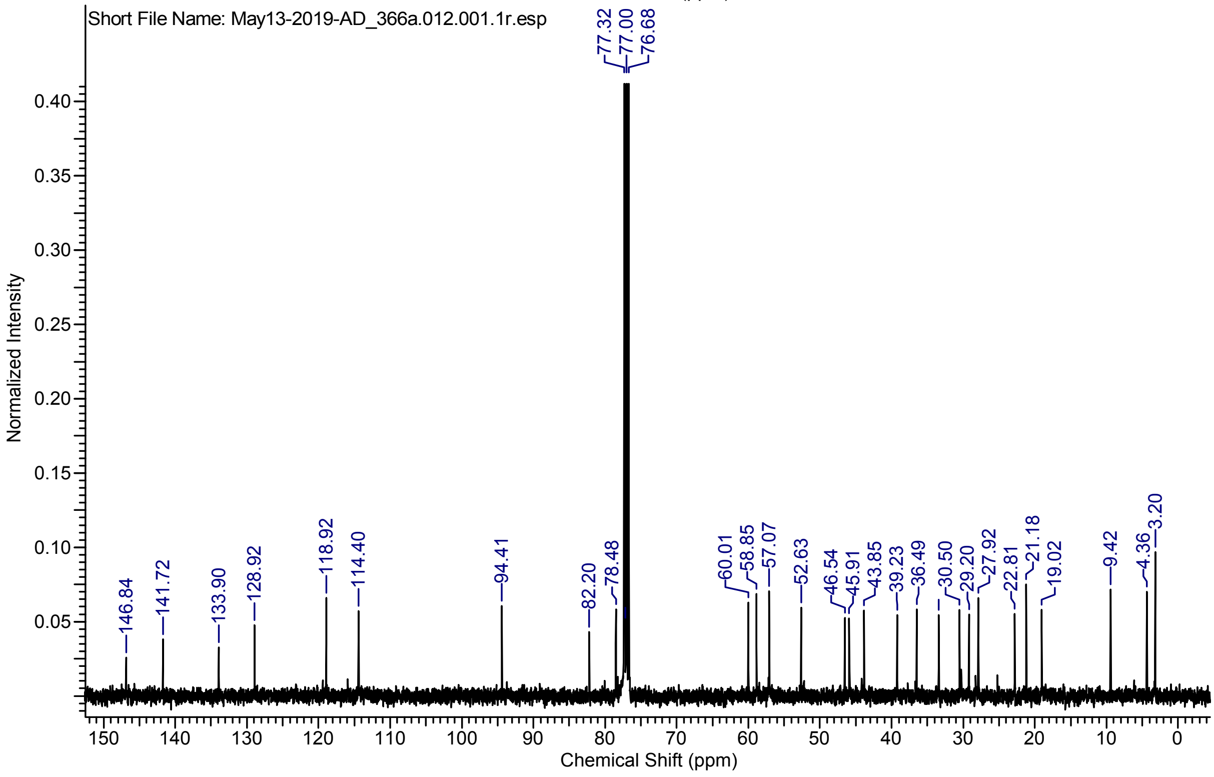

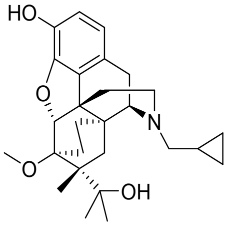

(5α,6R,7R,14α)-2′-(17-Cyclopropylmethyl-4,5-epoxy-7,8-dihydro-3-hydroxy-6-methoxy-7β-methyl-6,14-ethano-morphinan-7-yl)-propan-2′-ol.HCl (20.HCl)

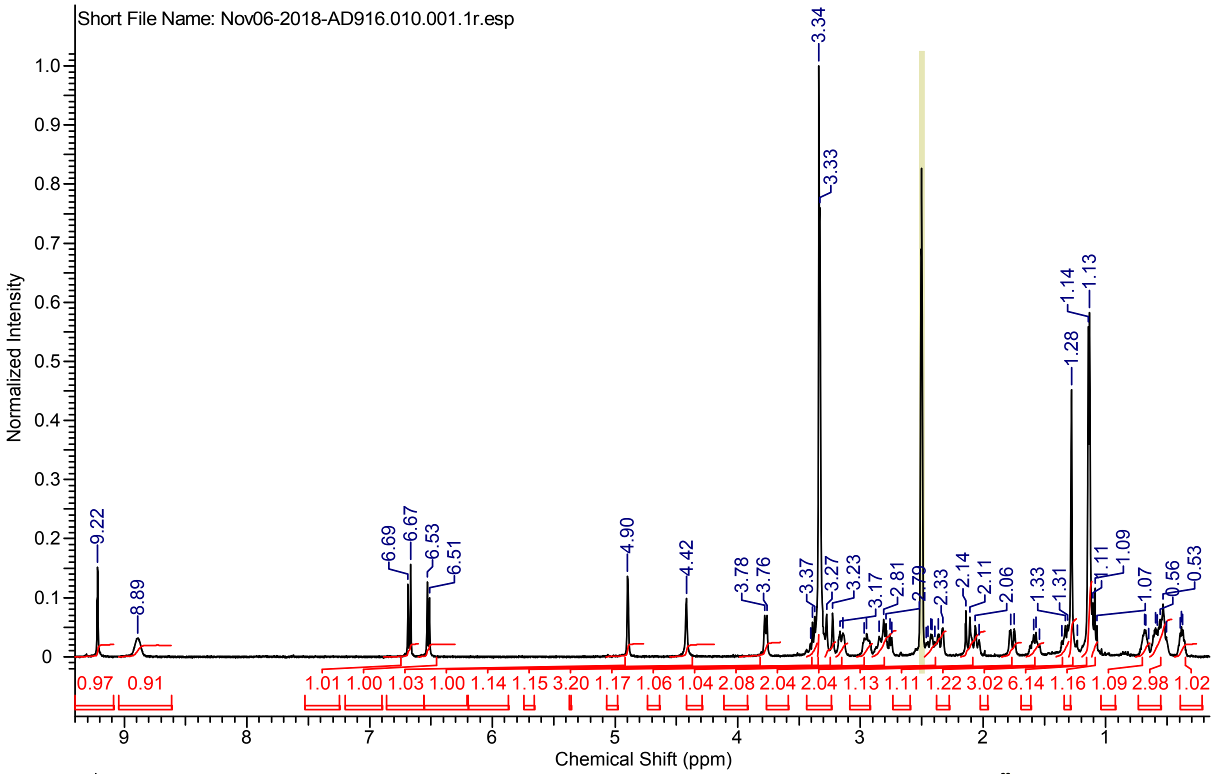

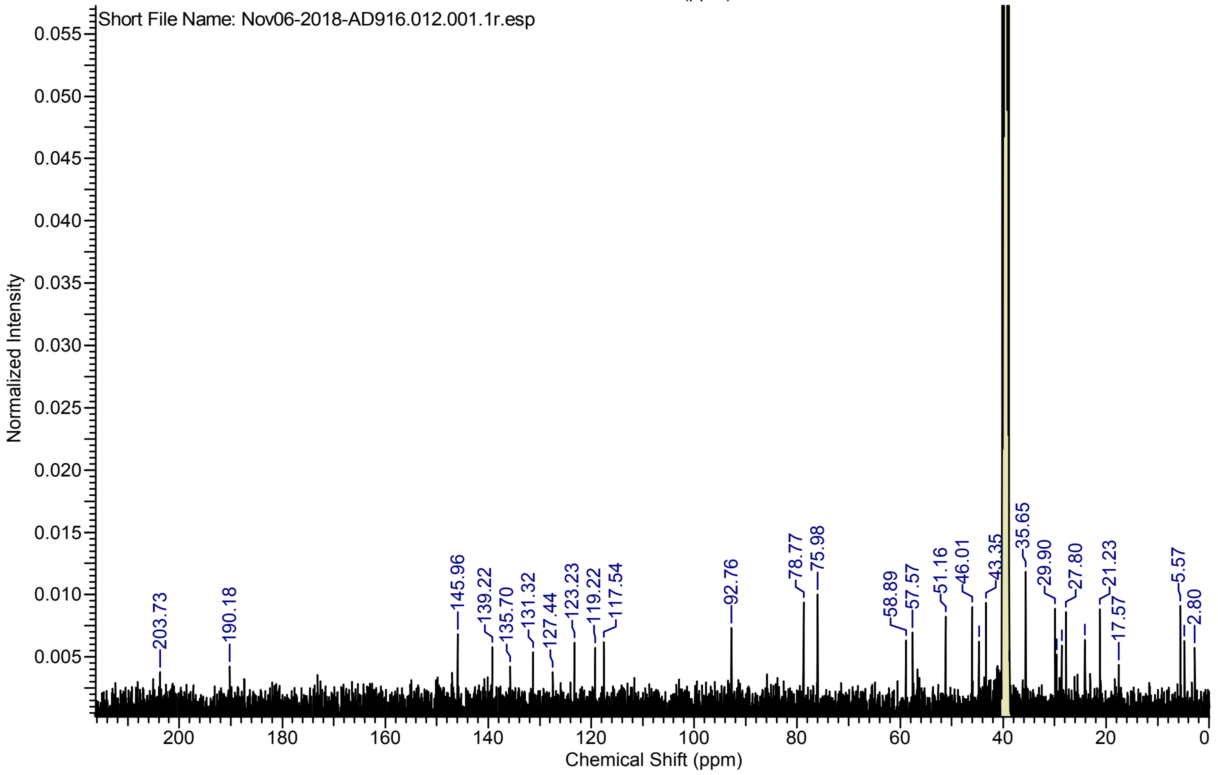

HPLC of 20 at 254 nm

Chromatogram Report

Analysis Info

Analysis Name	C:\Data\2019-01-04 Alex RP C18\AD333 HCl_RC2_01_4262.d	Acquisition Date	<illegal time>
Method		Operator	
Sample Name		Instrument	
Comment			

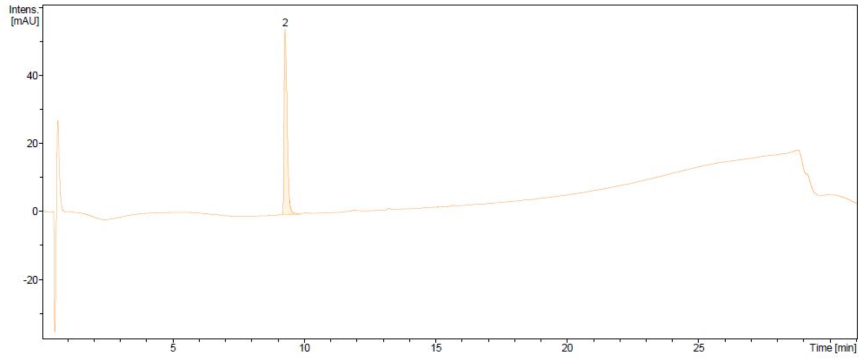

#	RT [min]	Area	Area Frac. %
2	9.3	444.822	100.00

Bruker Compass DataAnalysis 4.3 printed: 15-Jan-19 10:47:35 AM by: bdal 1 of 1

HPLC of 20 at 280 nm

Chromatogram Report

Analysis Info

Analysis Name	C:\Data\2019-01-04 Alex RP C18\AD333 HCl_RC2_01_4262.d	Acquisition Date	<illegal time>
Method		Operator	
Sample Name		Instrument	
Comment			

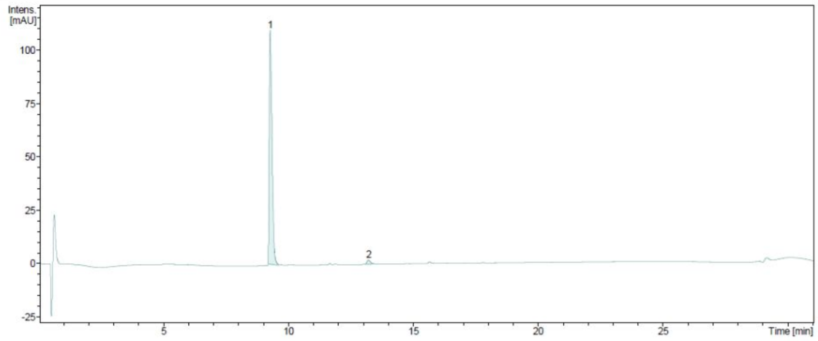

#	RT [min]	Area	Area Frac. %
1	9.3	870.745	97.85
2	13.2	19.143	2.15

Bruker Compass DataAnalysis 4.3 printed: 15-Jan-19 10:44:49 AM by: bdal 1 of 1
